# Aryl carbonyls and carbinols as proelectrophiles for Friedel–Crafts benzylation and alkylation†

**DOI:** 10.1039/d4ra02213k

**Published:** 2024-05-13

**Authors:** P. Veeraraghavan Ramachandran, Randy Lin, Abdulkhaliq A. Alawaed, Henry J. Hamann

**Affiliations:** a Department of Chemistry, Herbert C. Brown Center for Borane Research, Purdue University West Lafayette Indiana 47907 USA chandran@purdue.edu

## Abstract

Friedel–Crafts benzylation/alkylation using benzylic, tertiary, and homobenzylic alcohols; aryl aldehydes, aryl ketones, and the highly challenging aryl carboxylic acids and esters as proelectrophiles has been achieved using borane-ammonia and TiCl_4_, greatly broadening the scope of useable substrates. Incorporation of deactivated aromatic proelectrophiles and specificity for substitution at the benzylic position are demonstrated in the synthesis of various di- and triarylalkane products. Dual protocols allow for the use of standard nucleophilic solvents (benzene, toluene, *etc.*) or for stoichiometric addition of more valuable nucleophiles including furans, thiophenes, and benzodioxoles.

## Abstract 2

Friedel–Crafts benzylation/alkylation using benzylic alcohols and aryl carbonyls as proelectrophiles is achieved using borane-ammonia and TiCl_4_.

## Graphical abstract

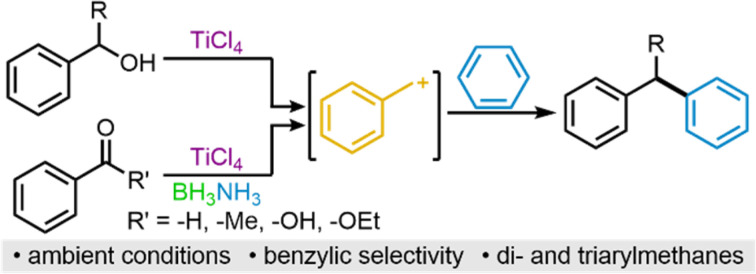

## Introduction

Friedel–Crafts alkylation and acylation, discovered nearly 150 years ago,^1^ are among the most highly studied reactions in organic chemistry, each having been utilized in numerous chemical industries. A specialized variant, benzylation, is often used for the production of 1,1-diarylalkanes^2^ and 1,1,1-triarylalkanes^3^ which play important roles in pharmaceuticals,^4^ electroactive polymers,^5^ and as dyes.^6^ Other protocols frequently used to access these polyaryl alkane species typically call for transition metal-catalyzed^7–15^ or base promoted^16^ cross coupling between appropriate benzyl/aryl halides and benzyl/aryl nucleophiles.

**Scheme 1:**
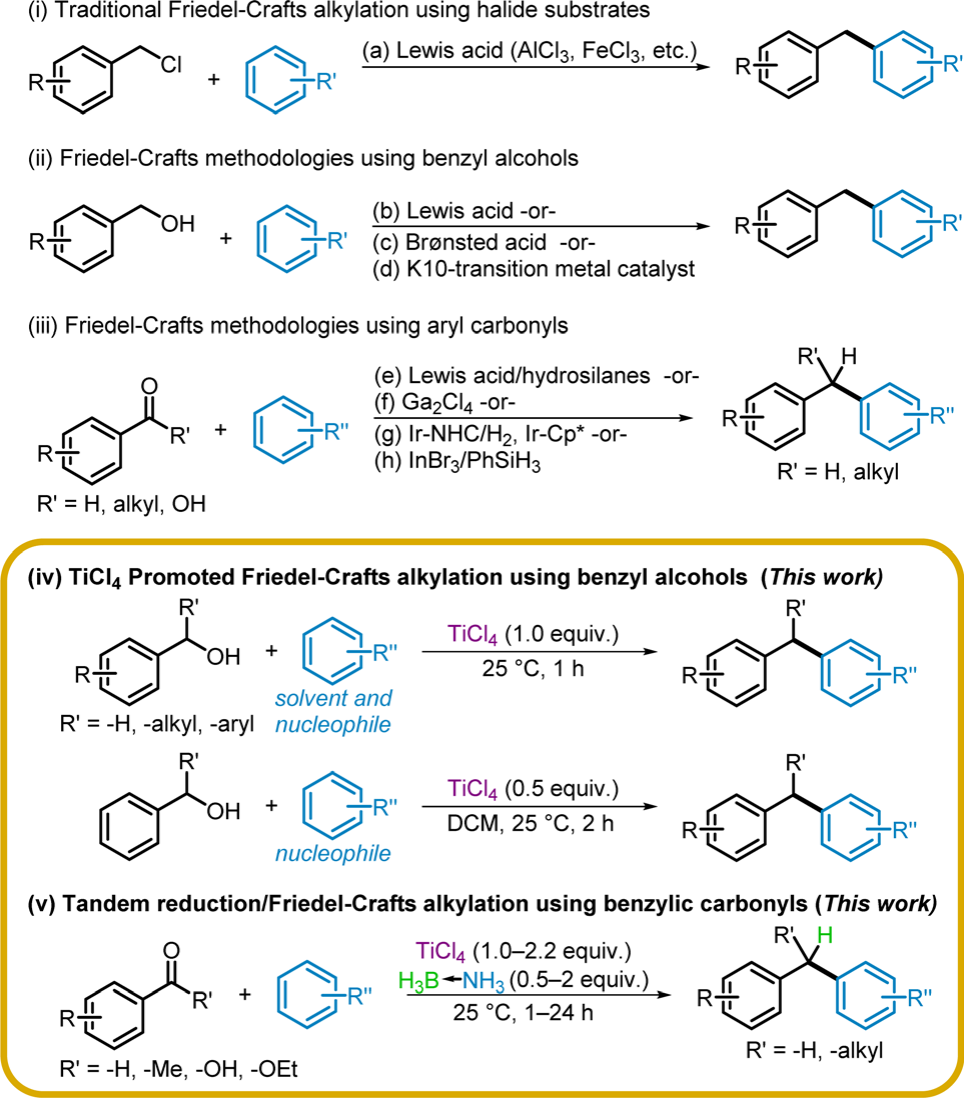
Approaches to Friedel–Crafts alkylation.

Since the initial report, numerous attempts have been made to expand the class of proelectrophiles for Friedel–Crafts alkylation. Initially developed using alkyl halides (Scheme 1i),^17^ the alkylation reaction has since expanded to include benzyl alcohols by utilizing Lewis acids, such as TeCl_4_,^18^ Sc(OTf)_3_,^19^ Mo(CO)_6_,^20^ FeCl_3_,^21^ TfOH,^22^ and NbCl_5_ (Scheme 1ii).^23^ Brønsted acids such as *p*-toluenesulfonic acid monohydrate^24^ and H_2_SO_4_,^25^ as well as transition-metal doped Montmorillonite clay,^26^ have also been reported as alternate catalysts.

Procedures for tandem reduction and alkylation using aryl carbonyls, such as benzaldehydes and acetophenones, have been developed using various transition metal catalysts including Ga_2_Cl_4_,^27^ Sc(OTf)_3_,^28^ InCl_3_,^29^ FeCl_3_,^30^ and Ir-complex catalysts (Scheme 1iii).^31,32^ To the best of our knowledge, there has only been one method, reported by the Sakai group, for tandem reduction and alkylation starting from the corresponding aryl acids using InBr_3_ and phenylsilane (Scheme 1iii).^33^ Although aryl esters have been used as reagents in Friedel–Crafts type alkylations, typically cleavage of the ester results in alkylation of the aryl solvent solely by the corresponding alcohol,^34^ or by both alcohol and carboxylic acid, forming a mixture of products.^35^

Despite the advancements made with regard to suitable electrophiles for Friedel–Crafts alkylation/benzylation, the aforementioned protocols suffer from significant drawbacks. Frequently, expensive, transition metal catalysts, and/or sensitive silane reducing agents are necessary. Furthermore, these methods are carried out under elevated temperatures. Due to the long-standing interest in the Friedel–Crafts reactions and the increasing interest in 1,1-diarylalkane functionality, the expansion of suitable electrophiles is of great benefit.

Borane-amines,^36^ and borane-ammonia^37^ specifically, are gaining prominence as air and moisture stable alternatives to traditional hydride sources, including borohydrides and silanes. Our long-time interest in borane-ammonia has recently led to its application in protocols for the safe reduction of carboxylic acids,^38^ deoxygenation of esters to ethers,^39^ and the deoxyhalogenation of aryl carbonyls and carbinols,^40^ each method being promoted by TiCl_4_. In continuation, we report herein the extension of the TiCl_4_ and borane-ammonia system to Friedel–Crafts alkylation. The multitude of advantages of this process include the incorporation of a broad range of proelectrophiles, such as benzylic alcohols, aryl aldehydes, ketones, carboxylic acids, and, for the first time, esters, and further extension to tertiary and homobenzylic alcohol substrates. This has been accomplished, unlike prior methodologies, by utilizing a single set of reagents, TiCl_4_ and borane-ammonia, under ambient conditions. These protocols permit the synthesis of a diverse range of di- and triarylalkane products, including those incorporating sulfur and oxygen containing heteroaromatic groups, and enable chemoselective substitution at the benzylic position of the substrate proelectrophiles, even in the presence of other haloalkyls.

## Results and discussion

**Scheme 2:**
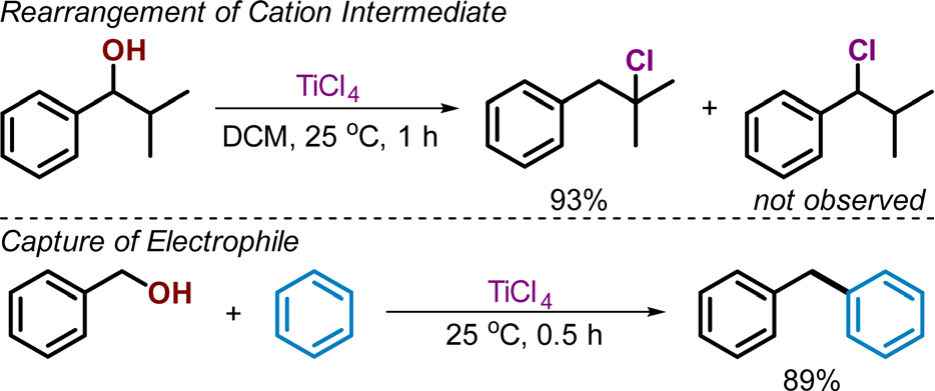
Initial observation of carbocation formation.

During our investigation of the mechanism of the deoxyhalogenation of aryl carbonyls and carbinols,^40^ we reacted 2-methyl-1-phenylpropan-1-ol with TiCl_4_ in dichloromethane to form the corresponding chloride. However, a rearranged tertiary chloride was observed (Scheme 2), indicating the intermediacy of a carbocation. To validate the formation of this intermediate, benzyl alcohol was reacted with 1 equiv. TiCl_4_ in benzene. This provided diphenylmethane in 89% yield confirming the suspected cation formation (Scheme 2).

**Scheme 3:**
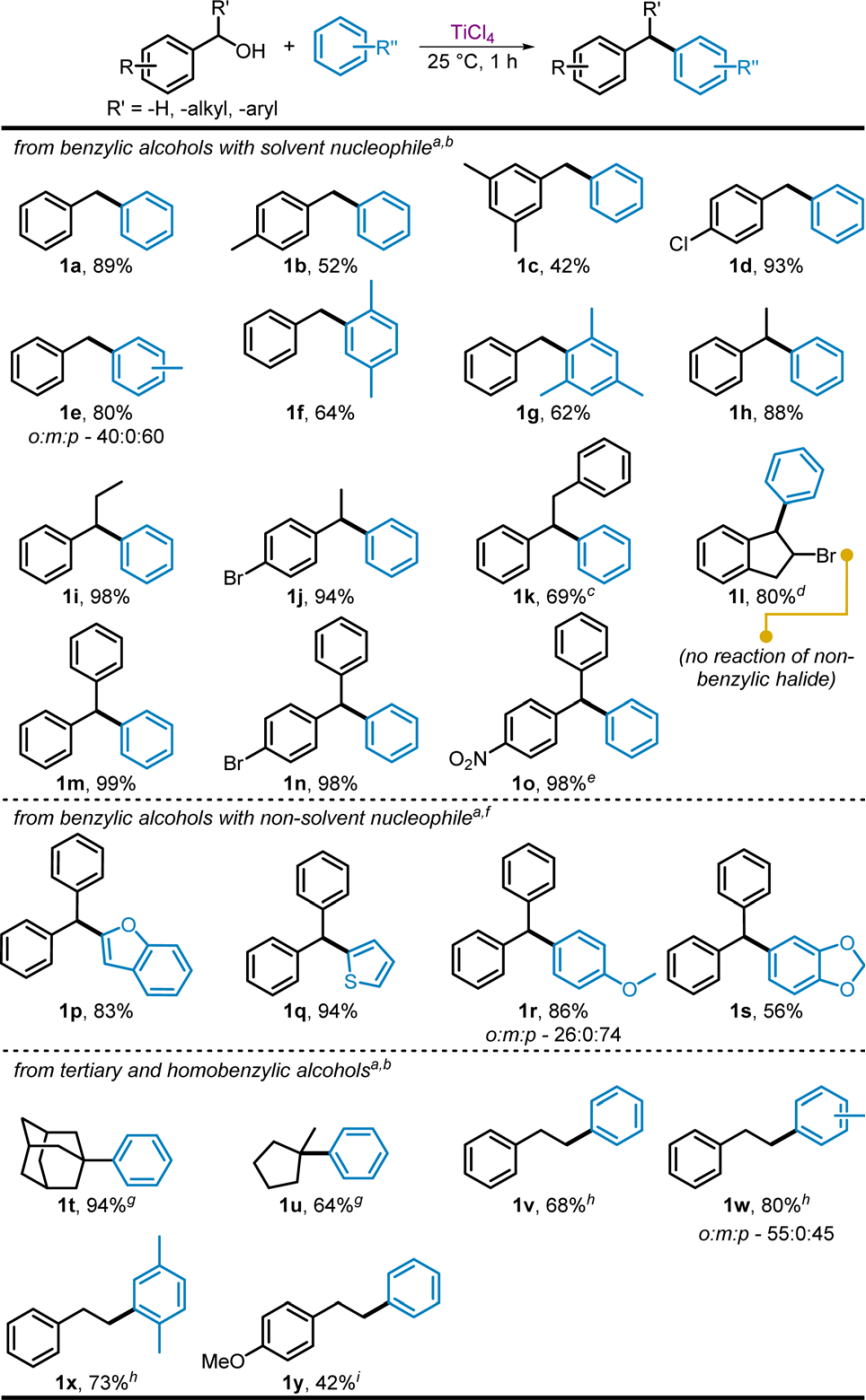
TiCl_4_-mediated Friedel–Crafts benzylation using alcohol proelectrophiles. ^*a*^Isolated yields, ^*b*^1 equiv. of benzylic alcohol 0.33 M in benzene (or other aromatic solvent), 1 equiv. TiCl_4_, RT for 1 h, ^*c*^12 h, ^*d*^3 h, ^*e*^24 h, ^*f*^1 equiv. of benzylic alcohol 0.33 M in dichloromethane, 1 equiv. nucleophile, 0.5 equiv. TiCl_4_, RT for 1 h, ^*g*^20 h, ^*h*^24 h at reflux, ^*i*^48 h at reflux.

Seeking to expand on this observed Friedel–Crafts alkylation, we applied these conditions (benzylic alcohol 0.5 M in nucleophilic solvent, with 1 equiv. of TiCl_4_) to produce a variety of di- and tri-arylmethanes from benzylic alcohols (Scheme 3). Primary benzyl alcohol examples containing either electron-donating or -withdrawing substituents were also used to generate the corresponding diarylmethanes (1b–1d). 4-Chlorodiphenylmethane 1d (93%) displayed a yield comparable to 1a (89%), however, aryl alcohols bearing electron donating methyl groups, particularly 1b and 1c, provided lower than expected yields, 52% and 42% respectively. This is attributed to the potential for oligomerization of the respective activated substituents, requiring separation of product from byproduct *via* chromatography. This effect was even more pronounced for highly activated methoxy-containing benzylic alcohols, where none of the desired products were obtained. This oligomerization is common among Friedel–Crafts alkylations. 4-Hydroxy- and 4-dimethylaminobenzyl alcohol likewise did not provide the corresponding Friedel–Crafts products. This incompatibility with certain functional groups is due to undesired coordination/reaction with the Lewis acid. A reaction of 4-nitrobenzyl alcohol was also attempted, although only starting material was obtained due to destabilization of the ostensible carbocation intermediate. Literature precedence and repeat attempts showed that even elevated temperatures and extended reaction times (>48 h) provided very low yields of the expected product.^41^

Substituted arene solvents including toluene, *p*-xylene, and mesitylene were also reacted with benzyl alcohols to provide the benzylated diarylmethane products 1e–1g in moderate yields (62–80%). The lower yields for the *p*-xylene and mesitylene containing products (1f and 1g) may be explained by the bulk of the nucleophile. The ratio of *ortho*- and *para*-substitution in the reaction of toluene was 40 : 60, with *meta*-substitution product not detected, similar to previously reported Friedel–Crafts reactions of benzyl chloride with TiCl_4_ in toluene.^42^

Various secondary benzylic alcohols were also tested and found to provide good to excellent yields with benzene when the α-substituent of the alcohol was a methyl (1h and 1j), ethyl (1i), or phenylmethyl (1k) group. The reaction of 2-bromo-1-indanol in benzene provided 1l in 80% yield within 3 h. Importantly for this substrate; none of the preexisting bromo group reacted to form a secondary Friedel–Crafts product, substitution was observed only at the benzylic position containing the alcohol.

Triarylmethane products 1m and 1n were produced from the corresponding diphenylmethanols and benzene, each in nearly quantitative yield. Notable, the reaction of the highly deactivated (4-nitrophenyl)(phenyl)methanol provided a 55 : 45 ratio of the corresponding benzhydryl chloride product to the desired 1o after 1 h. Increasing the reaction time to 24 h, provided 1o in 98% yield.

Extending the reaction to include non-solvent nucleophiles (Scheme 3), a modified procedure reacting diphenylmethanol (1 equiv.) with the desired nucleophile (1 equiv.) and TiCl_4_ (0.5 equiv.) in dichloromethane was utilized. A reaction of diphenylmethanol and thiophene with a lower 0.1 equiv. TiCl_4_ was also performed to determine if TiCl_4_ was acting catalytically. However, no observable product was obtained from this reaction which provided a mixture of unreacted starting material along with a small portion of the corresponding benzhydryl chloride from diphenylmethanol.

The conditions using 0.5 equiv. TiCl_4_ gave products 1p–1s in 56% to 94% yields. This included various oxygen (1p and 1s) and sulfur (1q) containing heterocycles, which were each obtained as a single substitution product. It is noteworthy that product 1q has been highlighted previously^43^ as a useful antitubercular analog and is produced rapidly under ambient conditions with our protocol. Surprisingly, methoxy groups were tolerated on the nucleophile, and the reaction of highly activated anisole gave an 86% yield of (1r) in a 26 : 74 ratio of the *ortho*- to *para*-substituted products. Attempted reactions of nitrogen containing heterocyclic nucleophiles were unsuccessful. *N*,*N*-Dimethylaniline and *N*-phenyl-3,3,3-trifluoropropanamide, an amide protected nitrogen, were also examined as potential activated nucleophiles in reactions with diphenylmethanol. In both cases unreacted nucleophile was recovered along with the corresponding benzhydryl chloride from diphenylmethanol. Phenol was found to provide *ortho*- and *para*-substituted products (identified by ^1^H NMR) with diphenylmethanol but they were not able to be separated from the proposed over addition and oligomeric products also present in the crude reaction mixture. Reactions of naphthalene and anthracene with benzyl alcohol likewise provided complicated mixture of over addition and oligomeric products, results which were again observed in the reactions of polyaromatic nucleophiles with aldehyde and ester substrates.

Aliphatic alcohols, 1-adamantol and 1-methycyclopentanol, were able to undergo arylation in benzene to provide 1t and 1u in 94% and 64% yields respectively, although longer reaction times of 20 h were required (Scheme 3). 1-Adamantol did not provide the arylated product when reacted with bulkier *p*-xylene. Homobenzylic alcohol, 2-phenylethanol, was successfully reacted in benzene, toluene, and *p*-xylene, providing 68% to 80% yields of bibenzyls 1v–1x. Refluxing conditions were necessary for these substrates, reflecting the further instability of the intermediate cations. Substituted 2-(4-methoxyphenyl)ethan-1-ol required 48 h of reflux to obtain 42% of 1y, but the nitro analogue was unreactive even under these conditions.

**Scheme 4:**
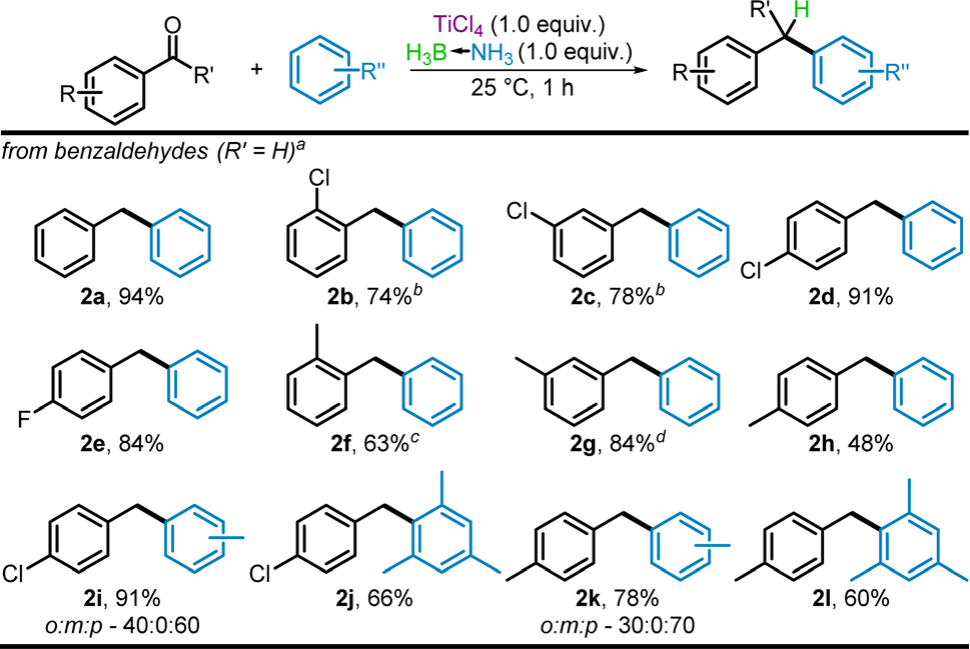
TiCl_4_-mediated Friedel–Crafts benzylation using benzaldehyde proelectrophiles. ^*a*^Isolated yields, ^*b*^20 h and 2 equiv. TiCl_4_, ^*c*^20 h, ^*d*^2 h.

Following the success of the Friedel–Crafts alkylations with benzylic alcohol, we next focused on incorporating carbonyls as possible substrates. During optimization of the reactions conditions (summarized in the ESI†), adapted from our prior deoxyhalogenation protocol,^40^ an equimolar stoichiometry of aldehyde, BH_3_NH_3_, and TiCl_4_ was determined. Based on our prior proven superiority of BH_3_NH_3_ for carbonyl reduction,^44^ other borane-amine adducts were not tested. For aryl aldehydes, a one-pot reduction/benzylation was possible utilizing TiCl_4_ and borane-ammonia in the desired nucleophilic solvent. Applying these conditions to benzaldehyde as a baseline substrate, diphenylmethane, 2a, was obtained in 94% yield (Scheme 4).

Evaluation of the substituent effect using mildly deactivated chlorobenzaldehydes revealed that the *para*-isomer gave diarylmethane 2d in 91% yield, but yields were decreased for the *ortho*- (2b) and *meta*-substituted (2c) products, 74% and 78% respectively. These results were reversed for the mildly activated tolualdehyde isomers where the *ortho*- (2f) and *para*-substituted (2h) products were obtained in 63% and 48% yields respectively, along with oligomerized byproducts. The reaction with *meta*-tolualdehyde gave 2g is 84% yield with no byproduct formation.

Other nucleophilic solvents, toluene and *p*-xylene, were used in the benzylation reaction with *p*-methyl- and *p*-chlorobenzaldehyde providing products 2i–2l in 60–91% yields. The reactions with toluene gave isomeric mixtures of 2i and 2k, with ratios of 40 : 0 : 60 and 30 : 0 : 70 respectively, for the *ortho*-, *meta*-, and *para*-products (determined by ^1^H NMR), indicating a decrease of *para*-specificity in the presence of even a mildly deactivating group. No oligomerized byproduct was detected in the reaction of 2k as further addition at the *meta*-position is typically unfavorable for such Friedel–Crafts reactions.^42^

When expanding the scope of the reaction to include aryl ketones, it was found that while aryl aldehydes underwent rapid reduction and electrophile formation, aryl ketones required a tandem reduction of the ketone (20% TiCl_4_ with 0.5 equiv. BH_3_NH_3_), followed by an additional 1.0 equiv. of TiCl_4_ to enable C–C bond formation (optimization details in ESI†).

**Scheme 5:**
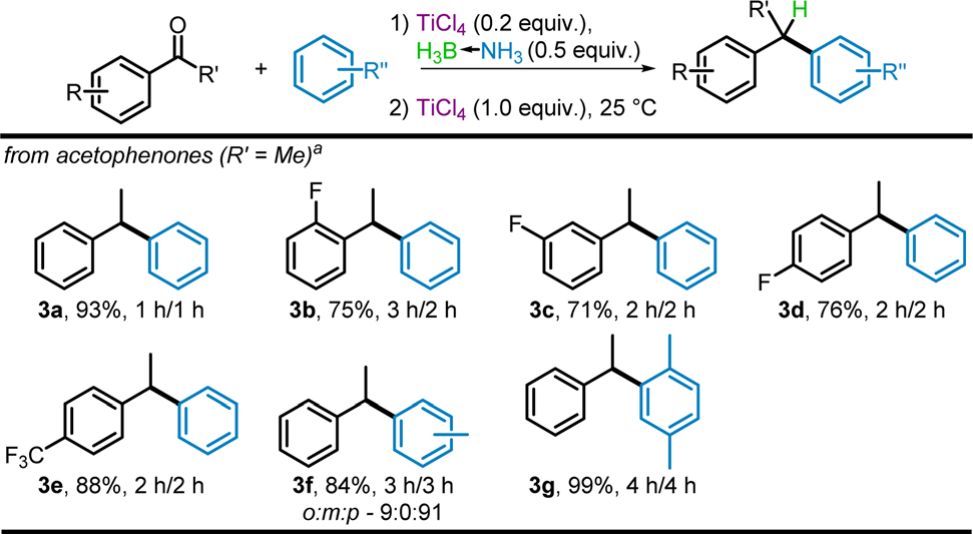
TiCl_4_-mediated Friedel–Crafts benzylation using substituted acetophenone proelectrophiles. ^*a*^Isolated yields.

The tandem reduction-benzylation using acetophenone in benzene provided diaryl 3a in 93% yield (Scheme 5). Extension to *ortho*-, *meta*-, and *para*-fluoro acetophenones provided 3b–3d in 71% to 76% yield, though a slight increase in reaction time was required. Despite the strongly withdrawing nature of *para*-trifluoromethyl substituted acetophenone, product 3e was obtained in higher yields (88%) than the products 3b–3d from mono-fluorinated ketones. Utilizing toluene and *p*-xylene as solvents for the tandem reduction–benzylation using acetophenone gave the corresponding products (3f and 3g) in 84% and 99% yield respectively. *Para*-substitution of toluene gave the predominant 3f isomer (9 : 0 : 91 for *ortho* : *meta* : *para*).

A reaction with *para*-methylacetophenone was attempted, but this provided a substantial portion (14%) of the corresponding deoxygenation product. The electron-donating methyl group present and increased stability of the benzylic, secondary carbocation formed as a reaction intermediate allow the formation of such deoxygenated products. These deoxygenation products were also observed for highly stabilized cations in our deoxyhalogenation work,^40^ as well as in a recent deoxygenation protocol based on our reports.^45^

Interested in the potential of a functional group selective Friedel–Crafts benzylation, a reaction using 4-acetylbenzoic acid (1 equiv.) with 20% TiCl_4_, 0.5 equiv. BH_3_NH_3_, and benzene (2 equiv.) as the nucleophile was performed to test the intramolecular competition between a ketone and carboxylic acid. Only starting material was recovered from this reaction however. Suspecting a reaction between the carboxylic acid and TiCl_4_, a second reaction using the corresponding ester (ethyl 4-acetylbenzoate) was undertaken. The material obtained from this reaction showed that the ketone functionality had been reduced and converted to the chloride, but no Friedel–Crafts product was detected. The selective deoxyhalogenation of the ketone in the presence of the ester indicates that the potential functional group selective Friedel–Crafts benzylation warrants further study.

**Scheme 6:**
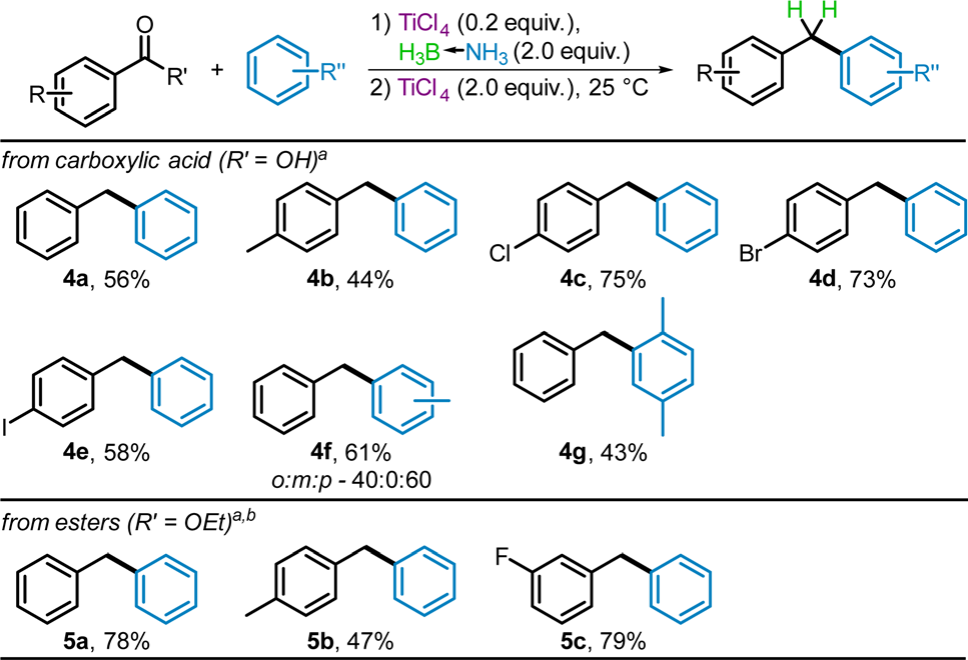
TiCl_4_-mediated Friedel–Crafts benzylation using benzoic acid and benzoate ester proelectrophiles. ^*a*^Isolated yields, ^*b*^1.5 equiv. of BH_3_NH_3_ used.

Very few reports^33^ have been made for the reduction and subsequent Fridel Crafts reaction using carboxylic acids and no reports were found for the corresponding reaction using esters. On the basis of our earlier reductions of carboxylic acids^38^ and esters,^39^ as well as their deoxyhalogenation,^40^ we examined the suitability of these substrates for the Friedel–Crafts benzylation using BH_3_NH_3_ and TiCl_4_. As seen in Scheme 6, reactions with both aryl carboxylic acids and aryl esters were successful. Compound 4a, from benzoic acid and benzene, was obtained in moderate yield, 56%; due to unreacted starting acid. The *para*-substituted methyl, chloro, bromo, and iodo benzoic acids gave products 4b–4e with benzene in 44% to 75% yields. As with other methyl substituted aromatic substrates, 4b displayed signs of oligomerization and required chromatographic separation. Electron-rich arenes (toluene and *p*-xylenes) gave diarylmethanes 4f and 4g in 61% and 43% yields respectively in their reactions with benzoic acid. The ratio of *ortho* to *para* substituted isomers of 4f (40 : 60) is identical to the ratio obtained for the same product made using benzyl alcohols (1e).

Benzylation products 5a–5c were produced from the reaction of the corresponding ethyl esters with benzene in 47% to 79% yields. As expected, the 4-methyl substituted 5b formed oligomerization byproducts and as such, had lower yields than the other diarylmethanes produced from esters.

## Conclusions

In conclusion, this work has demonstrated that Friedel–Crafts alkylation is achievable using benzylic alcohols as well as aryl aldehydes, ketones, carboxylic acids and esters as proelectrophilic substrates by adjusting the TiCl_4_ and BH_3_NH_3_ stoichiometry. Using nucleophilic solvents (benzene, toluene, *p*-xylene, mesitylene) the reaction provides di- and triarylmethane products in moderate to high yields at ambient temperature. An alternate protocol using dichloromethane as solvent allows for the use of other nucleophiles, including heteroaromatic furans and thiophenes, in a stoichiometry equimolar to the electrophile. These methods are particularly well suited to mildly deactivated aromatic substrates, and nitro, trifluoromethyl, fluoro, chloro, bromo, and iodo substituents are all well tolerated. Especially noteworthy is that absence of reaction of non-benzylic halogens, allowing for the selective benzylic attack by the nucleophile. The reaction has been further extended to include tertiary and homobenzylic alcohols as electrophile forming substrates. This novel protocol greatly broadens the scope of useable substrates for Friedel–Crafts benzylation/alkylation. The TiCl_4_/BH_3_NH_3_ reagent system efficiently promotes the reaction of each alcohol and carbonyl substrate, removing the need for the conversion of these functional groups into more reactive intermediates.

## Author contributions

P. V. Ramachandran: funding acquisition, project administration, writing – review and editing; R. Lin: data curation, investigation, methodology, writing – original draft; A. A. Alawaed: data curation, investigation, validation; H. J. Hamann: conceptualization, data curation, investigation, methodology, validation, visualization, writing – review and editing.

## Conflicts of interest

There are no conflicts to declare.

## Supplementary Material

RA-014-D4RA02213K-s001
