# Supplementary material for: Aryl carbonyls and carbinols as proelectrophiles for Friedel–Crafts benzylation and alkylation
Source: RSC Adv. 2024 May 13;14(22):15554–9. doi: 10.1039/d4ra02213k (PMC11090089; doi:10.1039/d4ra02213k)
Supplement: RA-014-D4RA02213K-s001 [file RA-014-D4RA02213K-s001.pdf]

Electronic Supplementary Material (ESI) for RSC Advances.
This journal is © The Royal Society of Chemistry 2024

SUPPORTING INFORMATION:

Aryl Carbonyls and Carbinols as Proelectrophiles for Friedel-Crafts Benzylation and Alkylation

P. Veeraraghavan Ramachandran,* Randy Lin, Abdulkhaliq A. Alawaed, and Henry J. Hamann

Herbert C. Brown Center for Borane Research, Department of Chemistry, Purdue University, West Lafayette, Indiana 47907, United States

**E-mail:* chandran@purdue.edu

Contents:

Page

General information S2

Reaction optimization........ S3-S5

General procedures for Friedel-Crafts reactions........ S6-S8

Characterization of products S9-S20

References S21-S22

NMR spectra of products S23-S69

General information:

All reagents and starting materials were purchased from Sigma-Aldrich (St. Louis, USA), Oakwood (Estill, USA) or Fisher Scientific (Waltham, USA) and used as received. Ammonium chloride, sodium borohydride, sodium bicarbonate, and reagent-grade tetrahydrofuran (75 to 400 ppm BHT) were used as received for preparation of ammonia-borane, which was prepared using a previously reported procedure. Solvents used for all reactions were distilled from sodium/benzophenone (diethyl ether), calcium hydride (dichloromethane), and stored under nitrogen, or used as received (hexanes, ethyl acetate, benzene, *p*-xylenes, mesitylene, chloroform). Thin-layer chromatography (TLC) was performed on F60 silica gel plates purchased from Macherey-Nagel (Allentown, USA) and visualized under UV light or ceric ammonium molybdate solution. Column chromatography was performed using 60 M Kieselgel silica gel. The identities of the products were confirmed by nuclear magnetic resonance (NMR) spectroscopy and measured in δ values in parts per million (ppm). Spectra of products were recorded on a Bruker (Billerica, USA) 400 MHz or a Bruker (Billerica, USA) 300 MHz.The ^{1}H NMR (400 MHz) spectra were recorded at ambient temperature and calibrated against the residual solvent peak of $CDCl_3$ (δ = 7.26 ppm) as an internal standard. Coupling constants (J) are given in hertz (Hz), and signal multiplicities are described of NMR data as s = singlet, d = doublet, dd = doublet of doublets, dt = doublet of triplets, ddd = doublet of doublet of doublets, ddt = doublet of doublet of triplets, dqd = doublet of quartet of doublets, dqt = doublet of quartet of triplets, dtd = doublet of triplet of doublets, dddd = doublet of doublet of doublet of doublets, t = triplet, td = triplet of doublets, tt = triplet of triplets, tdd = triplet of doublet of doublets, tdt = triplet of doublet of triplets, q = quartet, p = pentet, h = hextet, m = multiplet, and br = broad. The ^{13}C NMR (101 MHz or 75 MHz) spectra were recorded at ambient temperature and calibrated using CDCl3 (δ = 77.0 ppm) as an internal standard. ^{19}F NMR (376 MHz or 282 MHz) spectra were recorded at ambient temperature and chemical shifts are reported relative to the external standard $CFCl_3$ (δ = 0 ppm).

Reaction optimization

One-pot optimization of ketones using 4-bromoacetophenone

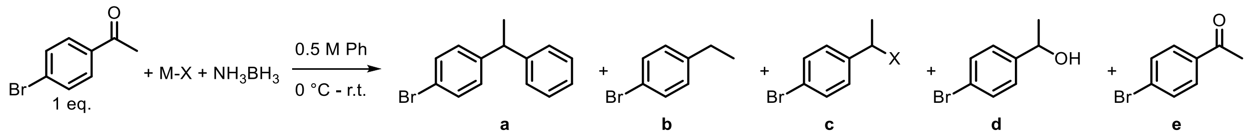

Scheme S1. Reaction scheme and possible products from 4-bromoacetophenone.

Entry	Lewis Acid	LA equiv.	AB equiv.	Time (hr)	product ratios				
					%a	%b	%c	%d	%e
1	$TiCl_4$	1	2	1	43	57	--	--	--
2	$TiCl_4$	1	1	1	72	28	--	--	--
3	$TiCl_4$	0.2	2	1	10	90	--	--	--
4	$TiCl_4$	0.5	1	1	--	31	56	13	--
5	$TiCl_4$	1	0.5	24	36	--	--	--	64
6	$TiCl_4$	1	0.6	24	--	--	40	--	60
7	$TiCl_4$	1	0.8	24	72	--	--	--	28
8	$TiBr_4$	1	1	24	8	--	45	--	47
9	$HfCl_4$	1	1	1	51	17	--	22	--
10	$HfCl_4$	1	0.5	24	78	--	--	--	22
11	$HfCl_4$	1	0.6	24	80	20	--	--	--
12	$HfCl_4$	1	0.8	24	73	27	--	--	--
13	$HfCl_4$	1.5	0.5	24	55				45
14	$HfCl_4$	1	0.5	24	36	trace	--	--	63
15	$HfCl_4$	1	0.5	24	24	61	15	--	24
16	$HfCl_4$	1	0.6	24	76	--	--	--	24

Table S1. Outcome of one-pot reduction/alkylation optimization using 4-bromoacetophenone.

Tandem reduction/alkylation optimization of ketones using 4-bromoacetophenone

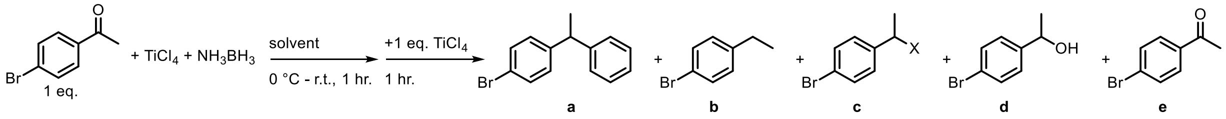

Scheme S2. Reaction scheme and possible products from 4-bromoacetophenone.

Entry	$TiCl_4$ equiv.	AB equiv.	Solvent	product ratios					Notes
				%a	%b	%c	%d	%e	
1	10%	0.5	Et_2O	--	--	--	--	--	intractable
2	10%	0.5	Ph	82	18	--	--	--	--
3	10%	0.4	Ph	--	31	56	13	--	--
4	10%	0.5	Ph	36	--	--	--	64	--
5	10%	0.5	Ph	--	--	40	--	60	--
6	10%	0.5	Ph	72	--	--	--	28	--

Table S2. Outcome of tandem reduction/alkylation optimization using 4-bromoacetophenone.

.

Tandem reduction/alkylation optimization of 4-chlorobenzoic acid

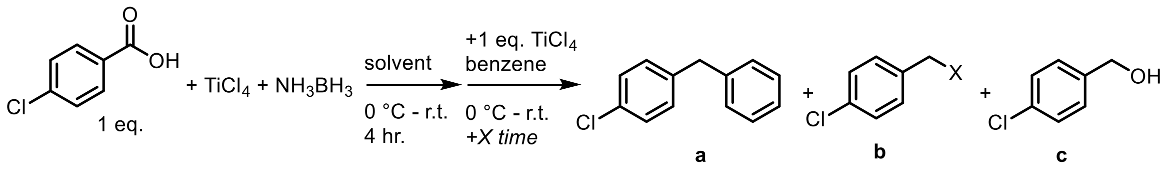

Scheme S3. Reaction scheme and possible products from 4-chlorobenzoic acid.

Entry	Initial $TiCl_4$ equiv.	AB equiv.	Solvent	*X* time	product ratios %a	%b	%c	Notes
1	20%	2	Ph	18	100	--	--	59% yield
2	100%	2	Ph	36	100	--	--	28% yield
3	20%	2	Et_2O	16	2	67	31	
4	20%	2	Et_2O	16	15	56	29	Initial rxn. time +20 h

Table S3. Outcome of tandem reduction/alkylation optimization using 4-chlorobenzoic acid.

Tandem reduction/alkylation optimization of 4-methylbenzoic acid

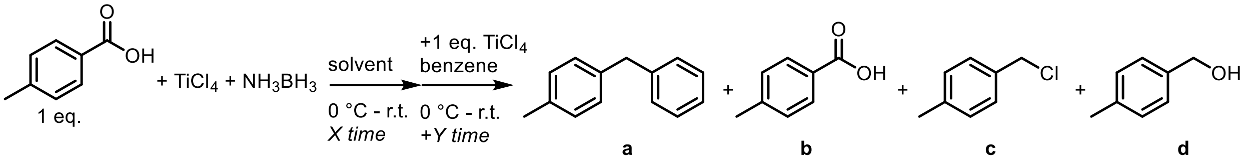

Scheme S4. Reaction scheme and possible products from 4-methylbenzoic acid.

Entry	Solvent	$TiCl_4$ eq.	AB eq.	X time (h)	Y time (h)	product ratios %a	%b	%c	%d	Notes
1	Et_2O	0.2	2	14	16	4	-	56	--	Messy
2	Ph	0.2	2	20	2	80	20	--	trace	
3	Ph	0.2	2	18	24	69	23	--	8	
4	Ph	0.2	3	24	2	71	23	--	6	
5	Ph	0.4	2	24	2	76	21	--	3	
6	Ph	0.6	2	18	2	90	--	--	10	26% yield
7	Ph	0.2	2	20	24	90	--	--	trace	40 °C 59% yield
8	Ph	0.2	2	24	24	84	--	--	16	+2 eq. $TiCl_4$ 42% yield

Table S4. Outcome of tandem reduction/alkylation optimization using 4-methylbenzoic acid.

Tandem reduction/alkylation optimization attempt of ethyl benzoates

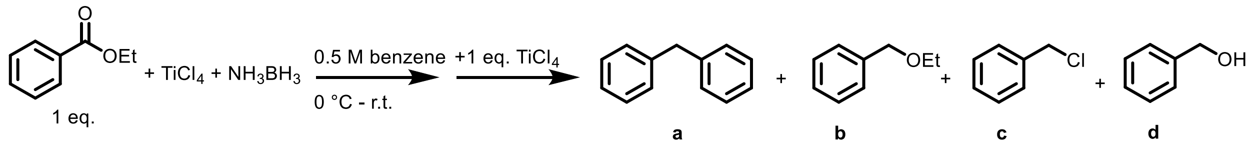

Scheme S5. Reaction scheme and possible products from ethyl benzoate.

Entry	$TiCl_4$ eq.	AB eq.	X time (h)	Y time (h)	product ratios				Notes
					%a	%b	%c	%d	
1	0.2	0.5	20	3	61	--	--	--	34% SM
2	0.2	1	24	2	65	--	--	--	35% SM
3	0.2	2	24	2	58	24	--	--	18% SM, 37% yield
4	0.2	0.5	20	3	35	--	--	--	65% SM
5	0.2	0.8	24	2	60	--	--	--	40% SM
6	0.4	0.8	24	2	78	--	--	--	22% SM
7	0.2	1.5	24	2	78	22	--	--	--
8	0.2	1	24	2	25	--	--	--	75% SM
9	0.2	1.5	4	3	94	--	--	--	6% SM

Table S5. Outcome of tandem reduction/alkylation optimization using ethyl benzoate.

One-pot reduction/alkylation optimization attempt of ethyl benzoates

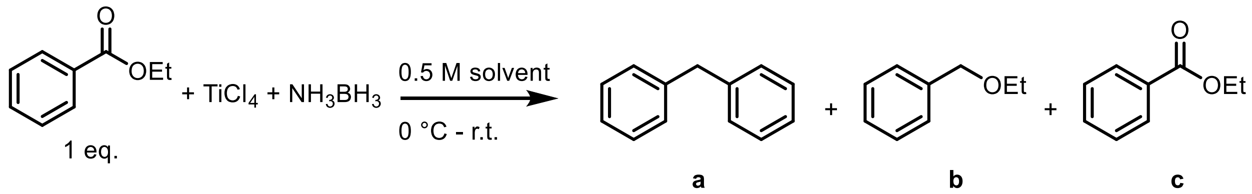

Scheme S6. Reaction scheme and possible products from ethyl benzoate.

Entry	Solvent	$TiCl_4$ eq.	AB eq.	Time (h)	product ratios			Notes
					%a	%b	%c	
1	Ph	2	2	16	77	--	23	64% yield
2	Ph	2	1.5	12	100	--	--	Optimal condition

Table S6. Outcome of one-pot reduction/alkylation optimization using ethyl benzoate.

General procedures for Friedel-Crafts reactions

General procedure for the preparation of Fridel-Crafts products from alcohols (solvent nucleophile) (GP1)

To a 15 mL oven dried round bottom flask was added alcohol (1 mmol, 1 equiv.) and a magnetic stirring bar. The reaction container was then sealed with a rubber septum followed by the addition of aryl solvent (3 mL, 0.33 M) to the round bottom flask and its contents were chilled to 0 °C via ice bath. $TiCl_4$ (0.1 mL, 1 equiv.) was then added dropwise to the reaction mixture. Upon complete addition, the reaction was stirred at 0 °C for 1 minute before being allowed to warm up to room temperature and was stirred until completion as determined by TLC. On completion of the reaction, the mixture was quenched with the addition of DI H_2O (5 mL) and allowed to stir for 1 minute, then transferred to a separatory funnel and extracted with DCM (3 x 5 mL). The combined organic layers were then dried over sodium sulfate, filtered through cotton, and concentrated under rotary evaporation, with the remaining solvent removed on high vacuum. Column chromatography was performed only if further purification was necessary.

General procedure for the preparation of Fridel-Crafts products from alcohols (non-solvent nucleophile) (GP2)

To a 15 mL oven dried round bottom flask was added alcohol (1 mmol, 1 equiv.) and a magnetic stirring bar. This was followed by the addition of dichloromethane (3 mL, 0.33 M) and the arene nucleophile (1 mmol, 1 equiv.) to the round bottom flask. The mixture was stirred and chilled to 0 °C using an ice bath. $TiCl_4$ (0.55 mL, 0.5 equiv.) was then added dropwise to the reaction mixture. Upon complete addition, the reaction was stirred at 0 °C for 1 minute before being allowed to warm up to room temperature and was stirred until completion as determined by TLC. On completion of the reaction, the mixture was quenched with the addition of DI H_2O (5 mL) and allowed to stir for 1 minute, then transferred to a separatory funnel and extracted with DCM (3 x 5 mL). The combined organic layers were then dried over sodium sulfate, filtered through cotton, and concentrated under rotary evaporation, with the remaining solvent removed on high vacuum. Column chromatography was performed only if further purification was necessary.

General procedure for the preparation of Fridel-Crafts alkylation products from phenyl ethanols (GP3)

To a 15 mL oven dried round bottom flask with side arm and condenser was added alcohol (1 mmol, 1 equiv.) and a magnetic stirring bar, followed by the addition of aryl solvent (5 mL, 0.2 M) to the round bottom flask. The reaction apparatus was sealed and flushed with nitrogen before the dropwise addition of $TiCl_4$ (0.1 mL, 1 equiv.) to the reaction mixture. Upon complete addition, the reaction was heated to reflux and was stirred for overnight (16 h). The mixture was then allowed to cool to room temperature before quenching with DI H_2O (5 mL) and allowed to stir for 1 minute. Consequently, the reaction mixture was transferred to a separatory funnel and extracted with EtOAc (3 x 5 mL). The combined organic layers were then dried over sodium sulfate, filtered through cotton, and concentrated under rotary evaporation, with the remaining solvent removed on high vacuum.

General procedure for the preparation of Fridel-Crafts alkylation products from aryl aldehydes (GP4)

To a 15 mL oven dried round bottom flask was added aldehyde (1 mmol, 1 equiv.) and a magnetic stirring bar. The reaction container was then sealed with a rubber septum and a needle connected

to an indirect N_2 line leading to an outlet was inserted through the septum. This was followed by the addition of aryl solvent (3 mL, 0.33 M) to the round bottom flask and its contents were chilled to 0 °C via ice bath. $TiCl_4$ (0.1 mL, 1 equiv.; 0.2 mL (2 equiv. for **2b** and **2c**) was then added dropwise to the reaction mixture. Once the Lewis acid was added, the septum was them removed to allow addition of borane-ammonia (31 mg., 1 equiv.) which was added directly to the reaction mixture followed by resealing of the reaction flask with the rubber septum with attached indirect line outlet. Upon complete addition, the reaction was stirred at 0 °C for 1 minute before being allowed to warm up to room temperature and was stirred until completion as determined by TLC. On completion of the reaction, the mixture was quenched with the addition of DI H_2O (5 mL) and allowed to stir for 1 minute, then transferred to a separatory funnel and extracted with DCM (3 x 5 mL). The combined organic layers were then dried over sodium sulfate, filtered through cotton, and concentrated under rotary evaporation, with the remaining solvent removed on high vacuum. Column chromatography was performed only if further purification was necessary.

General procedure for the preparation of Fridel-Crafts alkylation products from aryl ketones (GP5)

To a 15 mL oven dried round bottom flask was added ketone (1 mmol, 1 equiv.) and a magnetic stirring bar. The reaction container was then sealed with a rubber septum followed by the addition of aryl solvent (3 mL, 0.33 M) to the round bottom flask and its contents were chilled to 0 °C via ice bath. An indirect line leading to an outlet was also attached to the rubber septum. $TiCl_4$ (0.02 mL, 20 mol%) was then added dropwise to the reaction mixture. Once the Lewis acid was added, the septum was them removed to allow addition of borane-ammonia (16 mg., 0.5 equiv.) which was added directly to the reaction mixture followed by resealing of the reaction flask with the rubber septum with attached indirect line outlet. Upon complete addition, the reaction was stirred at 0 °C for 1 minute before being allowed to warm up to room temperature and stirred until starting material was no longer detectable according to TLC. After full consumption of starting material, the reaction was chilled again to 0 °C followed by the dropwise addition of $TiCl_4$ (1 mmol, 1 equiv.) and stirred for 1 minute before allowing to warm to room temperature. On completion of the reaction, the mixture was quenched with the addition of DI H_2O (5 mL) and allowed to stir for 1 minute, then transferred to a separatory funnel and extracted with DCM (3 x 5 mL). The combined organic layers were then dried over sodium sulfate, filtered through cotton, and concentrated under rotary evaporation, with the remaining solvent removed on high vacuum.

General procedure for the preparation of Fridel-Crafts alkylation products from aryl carboxylic acids (GP6)

To a 15 mL oven dried round bottom flask was added carboxylic acid (1 mmol, 1 equiv.) and a magnetic stirring bar. The reaction container was then sealed with a rubber septum followed by the addition of aryl solvent (3 mL, 0.33 M) to the round bottom flask and its contents were chilled to 0 °C via ice bath. An indirect line leading to an outlet was also attached to the rubber septum. $TiCl_4$ (0.02 mL, 20 mol%) was then added dropwise to the reaction mixture. Once the Lewis acid was added, the septum was them removed to allow addition of borane-ammonia (62 mg., 2 equiv.) which was added directly to the reaction mixture followed by resealing of the reaction flask with the rubber septum with attached indirect line outlet. Upon complete addition, the reaction was stirred at 0 °C for 1 minute before being allowed to warm up to room temperature and stirred for 20 h. The reaction was then chilled again to 0 °C followed by the dropwise addition of $TiCl_4$ (2 mmol, 2 equiv.) and stirred for 1 minute before allowing to warm to room temperature. On completion of the reaction as determined by TLC, the mixture was quenched with the addition of DI H_2O (5 mL) and allowed to stir for 1 minute, then transferred to a separatory funnel followed by 5 mL of saturated $NaHCO_3$ aqueous solution. The organic layer was extracted with DCM (3 x 5 mL) and the combined organic layers were then dried over sodium sulfate, filtered through

cotton, and concentrated under rotary evaporation, with the remaining solvent removed on high vacuum. Column chromatography was performed only if further purification was necessary.

General procedure for the preparation of Fridel-Crafts alkylation products from aryl esters (GP7)

To a 15 mL oven dried round bottom flask was added ester (1 mmol, 1 equiv.) and a magnetic stirring bar. The reaction container was then sealed with a rubber septum followed by the addition of aryl solvent (3 mL, 0.33 M) to the round bottom flask and its contents were chilled to 0 °C via ice bath. An indirect line leading to an outlet was also attached to the rubber septum. $TiCl_4$ (0.2 mL, 2 equiv.) was then added dropwise to the reaction mixture. Once the Lewis acid was added, the septum was them removed to allow addition of borane-ammonia (47 mg., 1.5 equiv.) which was added directly to the reaction mixture followed by resealing of the reaction flask with the rubber septum with attached indirect line outlet. Upon complete addition, the reaction was stirred at 0 °C for 1 minute before being allowed to warm up to room temperature and stirred for 20 h until completion as determined by TLC. If deemed incomplete by 20 h (**m-F**), the reaction was then chilled again to 0 °C followed by the dropwise addition of $TiCl_4$ (1 mmol, 1 equiv.) and stirred for 1 minute before allowing to warm to room temperature. On completion of the reaction as determined by TLC, the mixture was quenched with the addition of DI H_2O (5 mL) and allowed to stir for 1 minute, then transferred to a separatory funnel. The organic layer was extracted with DCM (3 x 5 mL) and the combined organic layers were then dried over sodium sulfate, filtered through cotton, and concentrated under rotary evaporation, with the remaining solvent removed on high vacuum. Column chromatography was performed only if further purification was necessary.

Product characterization

Characterization of Friedel-Crafts products from alcohols

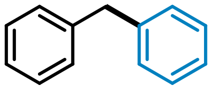

Diphenylmethane (1a); Prepared using method **GP1** and obtained as a colorless oil, mass = 144 mg, 89% yield; **^{1}H NMR** (400 MHz, $CDCl_3$) δ 7.32 – 7.25 (m, 4H), 7.21 (m, 6H), 4.00 (s, 2H). **^{13}C{^{1}H} NMR** (101 MHz, $CDCl_3$) δ 141.3, 129.1, 128.6, 126.2, 42.1. The spectral data is in accordance with previous reports.[1]

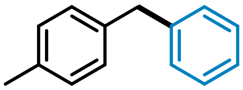

1-benzyl-4-methylbenzene (1b); Prepared using method **GP1** and obtained as a colorless oil, mass = 95 mg, 52% yield; **^{1}H NMR** (400 MHz, $CDCl_3$) δ 7.33 (m, 2H), 7.28 – 7.21 (m, 3H), 7.15 (s, 4H), 4.01 (s, 2H), 2.38 (s, 3H). **^{13} C{^{1}H} NMR** (101 MHz, $CDCl_3$) δ 141.5, 138.2, 135.7, 129.3, 129.0, 129.0, 128.6, 126.1, 41.7, 21.1. The spectral data is in accordance with previous reports.[1]

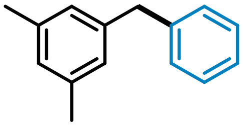

1-benzyl-3,5-dimethylbenzene (1c); Prepared using method **GP1** and obtained as a colorless oil, mass = 81 mg, 42% yield; **^{1}H NMR** (400 MHz, $CDCl_3$) δ 7.37 – 7.30 (m, 2H), 7.29 – 7.20 (m, 3H), 6.89 (d, J = 8.5 Hz, 3H), 3.97 (s, 2H), 2.34 (s, 6H). **^{13}C{^{1}H} NMR** (101 MHz, $CDCl_3$) δ 141.5, 141.1, 138.1, 129.0, 128.5, 127.9, 126.9, 126.1, 42.0, 21.4. The spectral data is in accordance with previous reports.[2]

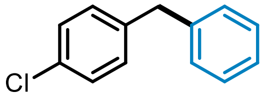

1-benzyl-4-chlorobenzene (1d); Prepared using method **GP1** and obtained as a colorless oil, mass =188 mg, 93% yield; **^{1}H NMR** (400 MHz, $CDCl_3$) δ 7.40 – 7.27 (m, 5H), 7.23 (d, J = 6.9 Hz, 2H), 7.17 (d, J = 7.4 Hz, 2H), 4.00 (s, 2H). **^{13}C{^{1}H} NMR** (101 MHz, $CDCl_3$) δ 140.7, 139.7, 132.0, 130.4, 129.0, 128.7, 126.4, 41.3. The spectral data is in accordance with previous reports.[1]

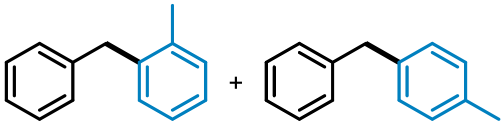

1-benzyl-2-methylbenzene + **1-benzyl-4-methylbenzene (1e);** Prepared using method **GP1** and obtained as a colorless oil, mass =146 mg, 80% yield; **^{1}H NMR** (400 MHz, $CDCl_3$) δ 7.33 (d, J = 7.6 Hz, 2H), 7.28 – 7.14 (m, 7H), 4.06 (s, 0.8H, *ortho*-isomer), 4.01 (s, 1.2H, *para*-isomer), 2.38 (s, 1.8H, *para*-isomer), 2.31 (s, 1.2H, *ortho*-isomer). **^{13}C{^{1}H} NMR** (101 MHz, $CDCl_3$) δ 141.5 (*para*-isomer), 140.5 (*ortho*-isomer), 139.1 (*ortho*-isomer), 138.2 (*para*-isomer), 136.7 (*ortho*-isomer), 135.6 (*para*-isomer), 130.1, 129.3, 129.0, 128.9, 128.5, 126.1, 41.7 (*para*-isomer), 39.6 (*ortho*-isomer), 21.2 (*para*-isomer), 19.8 (*ortho*-isomer). The spectral data is in accordance with previous reports. [1, 3]

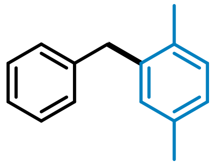

2-benzyl-1,4-dimethylbenzene (1f); Prepared using method **GP1** and obtained as a colorless oil, mass = 122 mg, 64% yield; **^{1}H NMR** (400 MHz, $CDCl_3$) δ 7.32 (t, *J* = 7.3 Hz, 2H), 7.24 (t, *J* = 7.3 Hz, 1H), 7.18 (d, *J* = 7.5 Hz, 2H), 7.11 (d, *J* = 7.7 Hz, 1H), 7.04 – 6.96 (m, 2H), 4.01 (s, 2H), 2.34 (s, 3H), 2.25 (s, 3H). **^{13}C{^{1}H} NMR** (101 MHz, $CDCl_3$) δ 140.7, 138.8, 135.5, 133.6, 130.9, 130.3, 128.8, 128.5, 127.2, 126.0, 39.6, 21.1, 19.3. The spectral data is in accordance with previous reports.[4]

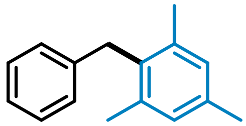

2-benzyl-1,3,5-trimethylbenzene (1g); Prepared using method **GP1** and obtained as a colorless oil, mass = 125 mg, 62% yield; **^{1}H NMR** (400 MHz, $CDCl_3$) δ 7.30 – 7.23 (m, 2H), 7.21 – 7.15 (m, 1H), 7.08 – 7.03 (m, 2H), 6.93 (s, 2H), 4.06 (s, 2H), 2.33 (s, 3H), 2.24 (s, 6H). **^{13}C{^{1}H} NMR** (101 MHz, $CDCl_3$) δ 140.3, 137.2, 135.8, 133.9, 129.0, 128.5, 128.0, 125.8, 34.8, 21.0, 20.3. The spectral data is in accordance with previous reports.[5]

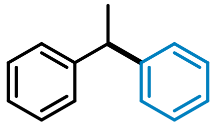

Ethane-1,1-diyldibenzene (1h); Prepared using method **GP1** and obtained as a colorless oil, mass = 160 mg, 88% yield; **^{1}H NMR** (400 MHz, $CDCl_3$) δ 7.33 – 7.27 (m, 4H), 7.26 – 7.22 (m, 4H), 7.22 – 7.17 (m, 2H), 4.17 (q, *J* = 7.2 Hz, 1H), 1.66 (d, *J* = 7.3 Hz, 3H). **^{13}C{^{1}H} NMR** (101 MHz, $CDCl_3$) δ 146.5, 128.5, 127.8, 126.2, 44.9, 22.0. The spectral data is in accordance with previous reports.[6]

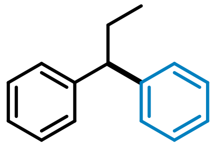

Propane-1,1-diyldibenzene (1i); Prepared using method **GP1** and obtained as a white solid, mass = 192 mg, 98% yield; **^{1}H NMR** (400 MHz, $CDCl_3$) δ 7.30 – 7.23 (m, 8H), 7.20 – 7.14 (m, 2H), 3.80 (t, *J* = 7.7 Hz, 1H), 2.08 (p, *J* = 7.4 Hz, 2H), 0.91 (t, *J* = 7.3 Hz, 3H). **^{13}C{^{1}H} NMR** (101 MHz, $CDCl_3$) δ 145.1, 128.3, 127.8, 125.9, 53.2, 28.5, 12.7. The spectral data is in accordance with previous reports.[7]

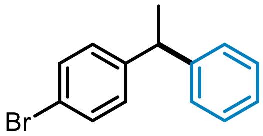

1-bromo-4-(1-phenylethyl)benzene (1j); Prepared using method **GP1** and obtained as a colorless oil, mass = 245 mg, 94% yield; **^{1}H NMR** (400 MHz, $CDCl_3$) δ 7.40 (d, *J* = 7.7 Hz, 2H), 7.31 – 7.25 (m, 2H), 7.22 – 7.16 (m, 3H), 7.09 (d, *J* = 8.5 Hz, 2H), 4.11 (q, *J* = 7.2 Hz, 1H), 1.62 (d, *J* = 7.2 Hz, 3H). **^{13}C{^{1}H} NMR** (101 MHz, $CDCl_3$) δ 145.6, 145.3, 131.3, 129.3, 128.4, 127.4, 126.2, 119.7, 44.1, 21.6. The spectral data is in accordance with previous reports.[8]

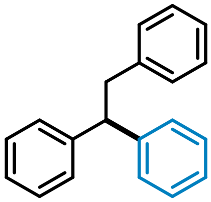

ethane-1,1,2-triyltribenzene (1k); Prepared using method **GP1** and obtained as a pale yellow solid, mass = 178 mg, 69% yield; **^{1}H NMR** (400 MHz, $CDCl_3$) δ 7.28 (s, 1H), 7.26 – 7.08 (m, 12H), 7.01 (d, *J* = 7.4 Hz, 2H), 4.24 (t, *J* = 7.8 Hz, 1H), 3.38 (d, *J* = 7.9 Hz, 2H). **^{13}C{^{1}H} NMR** (101 MHz, $CDCl_3$) δ 144.4, 140.2, 129.1, 128.3, 128.0, 126.2, 125.9, 53.1, 42.1. Compound characterization is in accordance with previous reports.[9]

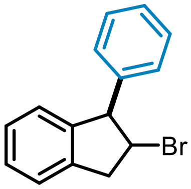

(1R)-2-bromo-1-phenyl-2,3-dihydro-1H-indene (1l); Prepared using method **GP1** and obtained as a white solid (Melting Point = 55-58 °C), mass = 218 mg, 80% yield); **^{1}H NMR** (400 MHz, $CDCl_3$ δ 7.38 – 7.27 (m, 4H), 7.26 – 7.15 (m, 4H), 6.96 (d, *J* = 7.5 Hz, 1H), 4.57 (d, *J* = 7.7 Hz, 1H), 4.42 (q, *J* = 7.6 Hz, 1H), 3.62 (dd, *J* = 16.1, 7.1 Hz, 1H), 3.39 (dd, *J* = 16.1, 8.0 Hz, 1H). **^{13}C{^{1}H} NMR** (101 MHz, $CDCl_3$) δ 143.7, 140.9, 140.8, 128.6, 128.4, 127.4, 127.3, 127.3, 124.9, 123.9, 61.5, 55.6, 42.8. **GCMS** m/z: calculated for $C_{15}H_{13}Br$: 272, found 272 (^{79}Br) and 274 (^{81}Br). **HRMS** (APCI+) m/z: calculated for $C_{15}H_{13}Br$: 273.0273, found 273.0268.

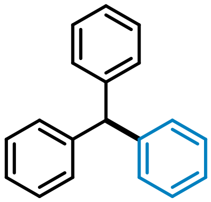

Triphenylmethane (1m); Prepared using method **GP1** and obtained as a white solid, mass = 264 mg, 99% yield; **^{1}H NMR** (400 MHz, $CDCl_3$) δ 7.33 – 7.26 (m, 6H), 7.25 – 7.19 (m, 3H), 7.16 – 7.11 (m, 6H), 5.57 (s, 1H). **^{13}C{^{1}H} NMR** (101 MHz, $CDCl_3$) δ 144.1, 129.6, 128.4, 126.4, 57.0. The spectral data is in accordance with previous reports.[7]

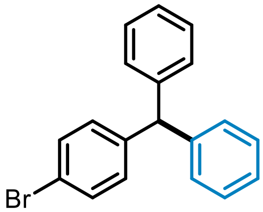

((4-bromophenyl)methylene)dibenzene (1n); Prepared using method **GP1** and obtained as a white solid, mass = 316 mg, 98% yield; **^{1}H NMR** (400 MHz, $CDCl_3$) δ 7.40 (d, *J* = 6.5 Hz, 2H), 7.31 – 7.27 (m, 4H), 7.26 – 7.21 (m, 2H), 7.09 (d, *J* = 8.1 Hz, 4H), 6.99 (d, *J* = 8.4 Hz, 2H), 5.50 (s, 1H). **^{13}C{^{1}H} NMR** (101 MHz, $CDCl_3$) δ 143.2, 142.9, 131.3, 131.1, 129.2, 128.3, 126.4, 120.2, 56.2. The spectral data is in accordance with previous reports.[10]

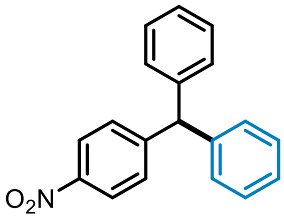

((4-nitrophenyl)methylene)dibenzene (1o); Prepared using method **GP1** and obtained as a pale yellow solid, mass = 283 mg, 98% yield; ^{1}**H NMR** (400 MHz, $CDCl_3$)) δ 8.14 (d, *J* = 8.7 Hz, 2H), 7.30 (m, 8H), 7.09 (d, *J* = 7.7 Hz, 4H), 5.64 (s, 1H). ^{13}**C{^{1}H} NMR** (101 MHz, $CDCl_3$) δ 151.5, 146.5, 142.2, 130.2, 129.2, 128.6, 126.9, 123.5, 56.6. Compound characterization is in accordance with previous reports.[10]

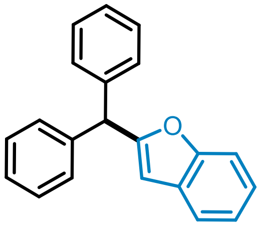

2-benzhydrylbenzofuran (1p); Prepared using method **GP2** and obtained as a white solid, mass = 236 mg, 83% yield; ^{1}**H NMR** (400 MHz, $CDCl_3$) δ 7.48 (d, *J* = 7.3 Hz, 1H), 7.42 (d, *J* = 7.7 Hz, 1H), 7.33 (d, *J* = 7.4 Hz, 4H), 7.25 (d, *J* = 7.9 Hz, 8H), 6.29 (s, 1H), 5.60 (s, 1H). ^{13}**C{^{1}H} NMR** (101 MHz, $CDCl_3$) δ 159.9, 155.1, 141.0, 128.9, 128.5, 126.9, 123.7, 122.6, 120.6, 111.1, 105.6, 51.3. Compound characterization is in accordance with previous reports.[11]

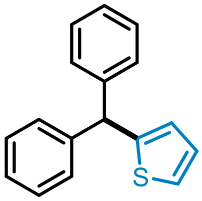

3-benzhydrylthiophene (1q); Prepared using method **GP2** and obtained as a white solid, mass = 235 mg, 94% yield; ^{1}**H NMR** (300 MHz, $CDCl_3$) δ 7.30 (t, *J* = 7.8 Hz, 4H), 7.26 – 7.18 (m, 7H), 6.94 (t, *J* = 4.4 Hz, 1H), 6.69 (d, *J* = 3.9 Hz, 1H), 5.68 (s, 1H) ^{13}**C{^{1}H} NMR** ^{13}C NMR (101 MHz, $CDCl_3$) δ 147.8, 143.7, 128.8, 128.3, 126.6, 126.5, 126.3, 124.5, 52.1. Compound characterization is in accordance with previous reports.[12]

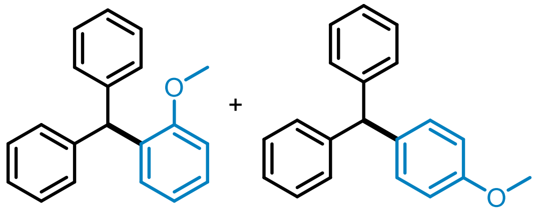

((2-methoxyphenyl)methylene)dibenzene + ((4-methoxyphenyl)methylene)dibenzene (1r); Prepared using method **GP2** and obtained as a whitish oil, mass = 236 mg, 86% yield; **^{1}H NMR** (400 MHz, $CDCl_3$) δ 7.35 – 7.28 (m, 3H), 7.26 – 7.20 (m, 3H), 7.17 – 7.12 (m, 3H), 7.10 – 7.01 (m, 3H), 6.96 – 6.79 (m, 2H), 5.92 (s, 0.26 H, *ortho*-isomer), 5.54 (s, 0.74 H, *para*-isomer), 3.80 (s, 2.22 H, *para*-isomer), 3.71 (s, 0.78 H, *ortho*-isomer). **^{13}C{^{1}H} NMR** (101 MHz, $CDCl_3$) δ 157.9 (*para*-isomer), 144.2 (*para*-isomer), 143.7 (*ortho*-isomer), 136.0, 131.7 (*ortho*-isomer), 130.3 (*para*-isomer), 129.3 (*para*-isomer), 129.2 (*ortho*-isomer), 128.2 (*para*-isomer), 128.1 (*ortho*-isomer), 127.9 (*ortho*-isomer), 126.1 (*para*-isomer), 126.0 (*ortho*-isomer), 125.8 (*ortho*-isomer), 113.6 (*para*-isomer), 110.3 (*ortho*-isomer), 55.9 (*para*-isomer), 55.6 (*ortho*-isomer), 55.1 (*para*-isomer), 49.4 (*ortho*-isomer). Compound characterization is in accordance with previous reports.[13]

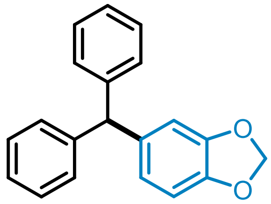

5-benzhydrylbenzo[d][1,3]dioxole (1s); Prepared using method **GP2** and obtained as a colorless oil, mass = 161 mg, 56% yield; **^{1}H NMR** (400 MHz, $CDCl_3$) δ 7.31 – 7.25 (m, 4H), 7.24 – 7.18 (m, 2H), 7.12 (dd, J = 7.0, 1.6 Hz, 4H), 6.73 (d, J = 7.9 Hz, 1H), 6.61 (d, J = 1.8 Hz, 1H), 6.57 (dd, J = 8.0, 2.1 Hz, 1H), 5.92 (s, 2H), 5.47 (s, 1H). **^{13}C{^{1}H} NMR** (101 MHz, $CDCl_3$) δ 147.6, 145.9, 143.9, 137.9, 129.3, 128.3, 126.3, 122.5, 109.9, 108.0, 100.9, 56.4. Compound characterization is in accordance with previous reports.[11]

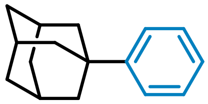

1-phenyladamantane (1t); Prepared using method **GP1** and obtained as a white solid, mass = 207 mg, 94% yield; **^{1}H NMR** (400 MHz, $CDCl_3$) δ 7.43 – 7.37 (m, 2H), 7.37 – 7.31 (m, 2H), 7.24 – 7.17 (m, 1H), 2.13 (bs, 3H), 1.95 (bs, 6H), 1.85 – 1.77 (m, 6H). **^{13}C{^{1}H} NMR** (101 MHz, $CDCl_3$) δ 151.2, 128.0, 125.4, 124.8, 43.1, 36.8, 36.1, 28.9. The spectral data is in accordance with previous reports.[14]

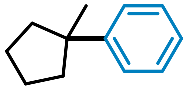

(1-methylcyclopentyl)benzene (1u); Prepared using method **GP1** and obtained as a yellow oil, mass = 106 mg, 63% yield; **^{1}H NMR** (400 MHz, $CDCl_3$) δ 7.43 – 7.27 (m, 4H), 7.25 – 7.19 (m, 1H), 2.03 – 1.73 (m, 8H), 1.32 (d, J = 2.2 Hz, 3H). **^{13}C{^{1}H} NMR** (101 MHz, $CDCl_3$) δ 151.6, 128.5, 128.2, 126.2, 125.7, 125.4, 47.3, 39.8, 29.7, 23.9. **GCMS** m/z: calculated for $C_{12}H_{16}$: 160, found 160. **HRMS** (APCI+) m/z: calculated for $C_{12}H_{16}$: 161.1324, found 161.1320.

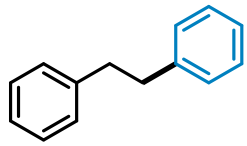

1,2-diphenylethane (1v); Prepared using method **GP3** and obtained as a colorless oil, mass = 123 mg, 68% yield; **^{1}H NMR** (300 MHz, $CDCl_3$) δ 7.22 – 7.14 (m, 4H), 7.14 – 7.05 (m, 7H), 2.83 (s, 4H). **^{13}C{^{1}H} NMR** (75 MHz, $CDCl_3$) δ 141.8, 128.5, 128.4, 126.0, 38.0. The spectral data is in accordance with previous reports.[15]

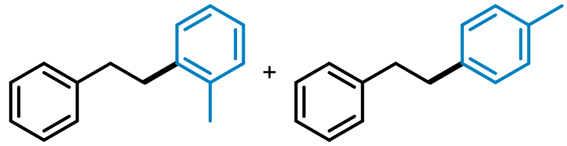

1-methyl-2-phenethylbenzene + 1-methyl-4-phenethylbenzene (1w); Prepared using method **GP3** and obtained as a whitish oil, mass = 157 mg, 80% yield; **^{1}H NMR** (400 MHz, $CDCl_3$) δ 7.24 – 7.15 (m, 2H), 7.10 (s, 3H), 7.04 (s, 2H), 6.99 (s, 1H), 6.93 (s, 1H), 2.80 (s, 4H), 2.23 (s, 1.65H, *ortho*-isomer), 2.21 (s, 1.35H, *para*-isomer). δ 7.40 – 7.27 (m, 5H), 7.23 (d, *J* = 6.9 Hz, 2H), 7.17 (d, *J* = 7.4 Hz, 2H), 4.00 (s, 2H). **^{13}C{^{1}H} NMR** (101 MHz, $CDCl_3$) δ 142.1, 142.0, 140.1, 136.0, 135.4, 130.3, 129.3, 129.1, 128.9, 128.5, 128.4, 126.7, 126.2, 126.1, 126.0, 125.9, 125.5, 38.1, 37.6, 36.8, 35.5, 21.1, 19.3. The spectral data is in accordance with previous reports.[15]

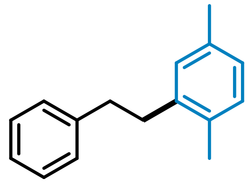

1,4-dimethyl-2-phenethylbenzene (1x); Prepared using method **GP3** and obtained as a brown residue, mass = 167 mg, 73% yield; **^{1}H NMR** (300 MHz, $CDCl_3$) δ 7.51 – 7.43 (m, 2H), 7.43 – 7.33 (m, 3H), 7.26 – 7.19 (m, 1H), 7.19 – 7.07 (m, 2H), 3.04 (s, 4H), 2.49 (s, 3H), 2.45 (s, 3H). **^{13}C{^{1}H} NMR** (75 MHz, $CDCl_3$) δ 142.2, 139.9, 135.4, 132.7, 130.2, 129.7, 128.4, 126.8, 126.0, 37.0, 35.6, 21.0, 18.8. The spectral data is in accordance with previous reports.[16]

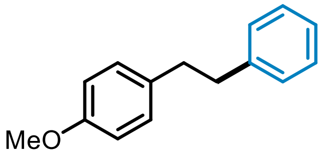

1-methoxy-4-phenethylbenzene (1y); Prepared using method **GP3** and obtained as a white solid, mass = 89 mg, 42% yield; **^{1}H NMR** (400 MHz, $CDCl_3$) δ 7.31 – 7.26 (m, 2H), 7.22 – 7.17 (m, 3H), 7.10 (d, *J* = 8.6 Hz, 2H), 6.83 (d, *J* = 8.6 Hz, 2H), 3.80 (s, 3H), 2.88 (s, 4H). **^{13}C{^{1}H} NMR** (101 MHz, $CDCl_3$) δ 157.8, 141.8, 133.8, 129.3, 128.4, 128.2, 125.8, 113.7, 55.2, 38.1, 37.0. The spectral data is in accordance with previous reports.[15]

Characterization of Friedel-Crafts products from aryl aldehydes

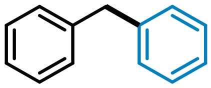

Diphenylmethane (2a); Prepared using method **GP4** and obtained as a colorless oil, mass = 158 mg, 94% yield; The spectral data is identical to **1a**.

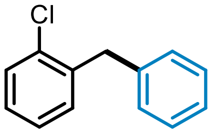

1-benzyl-2-chlorobenzene (2b); Prepared using method **GP4** (20 h and 2 equiv. $TiCl_4$) and obtained as a colorless oil, mass = 150 mg, 74% yield; **^{1}H NMR** (400 MHz, $CDCl_3$) δ 7.51 – 7.44 (m, 1H), 7.40 (d, *J* = 6.4 Hz, 2H), 7.31 (d, *J* = 6.5 Hz, 3H), 7.28 – 7.22 (m, 3H), 4.22 (s, 2H). **^{13}C{^{1}H} NMR** (101 MHz, $CDCl_3$) δ 139.6, 138.8, 134.3, 131.1, 129.6, 129.0, 128.6, 127.7, 126.9, 126.3, 39.3. The spectral data is in accordance with previous reports.[1]

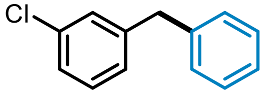

1-benzyl-3-chlorobenzene (2c); Prepared using method **GP4** (20 h and 2 equiv. $TiCl_4$) and obtained as a colorless oil, mass = 158 mg, 78% yield; **^{1}H NMR** (400 MHz, $CDCl_3$) δ 7.51 – 7.44 (m, 1H), 7.44 – 7.36 (m, 2H), 7.36 – 7.29 (m, 3H), 7.28 – 7.22 (m, 3H), 4.22 (s, 2H). **^{13}C{^{1}H} NMR** (101 MHz, $CDCl_3$) δ 143.3, 140.3, 134.4, 129.8, 129.1, 129.0, 128.7, 127.2, 126.5, 126.4, 41.7. The spectral data is in accordance with previous reports.[1]

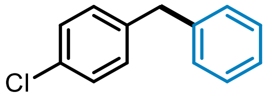

1-benzyl-4-chlorobenzene (2d); Prepared using method **GP4** and obtained as a pale yellow oil, mass = 190 mg, 91% yield; The spectral data is identical to **1d**.

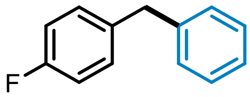

1-benzyl-4-fluorobenzene (2e); Prepared using method **GP4** and obtained as a colorless oil, mass = 162 mg, 84% yield; **^{1}H NMR** (400 MHz, $CDCl_3$) δ 7.38 (d, *J* = 7.3 Hz, 2H), 7.34 – 7.18 (m, 5H), 7.06 (t, *J* = 7.5 Hz, 2H), 4.04 (s, 2H). **^{13}C{^{1}H} NMR** (101 MHz, $CDCl_3$) ^{13}C NMR (101 MHz, Chloroform-*d*) δ 161.6 (d, *J* = 243.8 Hz), 141.1, 136.9, 130.4 (d, *J* = 7.8 Hz), 129.0, 128.7, 126.3, 115.3 (d, *J* = 21.0 Hz), 41.2. **^{19}F NMR** (282 MHz, $CDCl_3$) δ -117.3. The spectral data is in accordance with previous reports.[1]

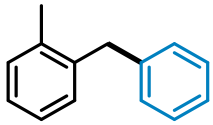

1-benzyl-2-methylbenzene (2f); Prepared using method **GP4** (20 h) and obtained as a colorless oil, mass = 115 mg, 63% yield; **^{1}H NMR** (400 MHz, $CDCl_3$) δ 7.33 – 7.27 (m, 2H), 7.24 – 7.10 (m, 7H), 4.02 (s, 2H), 2.27 (s, 3H). **^{13}C{^{1}H} NMR** (101 MHz, $CDCl_3$) δ 140.5, 139.1, 136.8, 130.4, 130.1, 128.9, 128.5, 126.6, 126.13, 126.05, 39.6, 19.8. The spectral data is in accordance with previous reports.[3]

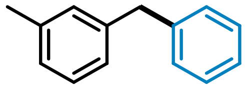

1-benzyl-3-methylbenzene (2g); Prepared using method **GP4** (2 h) and obtained as a colorless oil, mass = 156 mg, 84% yield; **^{1}H NMR** (400 MHz, $CDCl_3$) δ 7.40 – 7.34 (m, 2H), 7.32 – 7.23 (m, 4H), 7.14 – 7.06 (m, 3H), 4.04 (s, 2H), 2.40 (s, 3H). **^{13}C{^{1}H} NMR** (101 MHz, $CDCl_3$) δ 141.4, 141.2, 138.1, 129.8, 129.1, 128.6, 128.5, 127.0, 126.1, 42.0, 21.5. The spectral data is in accordance with previous reports.[17]

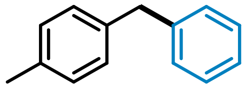

1-benzyl-4-methylbenzene (2h); Prepared using method **GP4** and obtained as a colorless oil, mass = 87 mg, 48% yield; The spectral data is identical to **1b**.

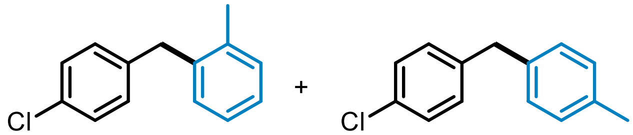

1-(4-chlorobenzyl)-2-methylbenzene + 1-(4-chlorobenzyl)-3-methylbenzene (2i); Prepared using method **GP4** and obtained as a colorless oil, mass = 197 mg, 91% yield; **^{1}H NMR** (400 MHz, $CDCl_3$) δ 7.32 (m, 2H), 7.27 – 7.23 (m, 1H), 7.15 (m, 5H), 4.02 (s, 0.8H, *ortho*-isomer), 3.98 (s, 1.2H, *para*-isomer), 2.41 (s, 1.8H, *para*-isomer), 2.31 (s, 1.2H, *ortho*-isomer). **^{13}C{^{1}H} NMR** (101 MHz, $CDCl_3$) ^{13}C NMR (101 MHz, Chloroform-*d*) δ 139.9, 138.9, 138.3, 137.5, 136.5, 135.8, 131.8, 131.7, 130.4, 130.2, 130.0, 129.9, 129.3, 128.7, 128.5, 128.5, 126.7, 126.1, 40.8 (*para*-isomer), 38.8 (*ortho*-isomer), 21.0 (*para*-isomer), 19.6 (*ortho*-isomer). The spectral data is in accordance with previous reports.[18]

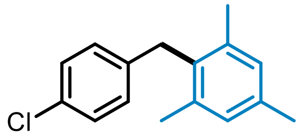

2-(4-chlorobenzyl)-1,3,5-trimethylbenzene (2j); Prepared using method **GP4** and obtained as a yellow oil, mass = 168 mg, 66% yield; **^{1}H NMR** (400 MHz, $CDCl_3$) δ 7.21 (d, *J* = 8.5 Hz, 2H), 6.96 (d, *J* = 8.1 Hz, 2H), 6.92 (s, 2H), 4.00 (s, 2H), 2.32 (s, 3H), 2.21 (s, 6H). **^{13}C{^{1}H} NMR** (101 MHz, $CDCl_3$) δ 138.7, 137.0, 136.1, 133.4, 131.5, 129.3, 129.1, 128.6, 34.2, 21.0, 20.2. The spectral data is in accordance with previous reports.[5]

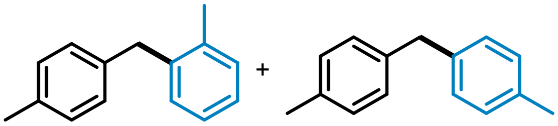

1-methyl-2-(4-methylbenzyl)benzene + 1-methyl-3-(4-methylbenzyl)benzene (2k); Prepared using method **GP4** and obtained as a colorless oil, mass = 153 mg, 78% yield; **^{1}H NMR** (400 MHz, $CDCl_3$) δ 7.22 (dd, *J* = 3.4, 1.8 Hz, 1H), 7.15 (d, *J* = 2.0 Hz, 6H), 7.08 (dt, *J* = 7.9, 2.6 Hz, 1H), 4.01 (s, 0.6H, *ortho*-isomer), 3.97 (s, 1.4H, *para*-isomer), 2.38 (s, 5H), 2.32 (s, 1H, *ortho*-isomer). **^{13}C{^{1}H} NMR** (101 MHz, $CDCl_3$) δ 139.2, 138.4, 137.4, 136.6, 135.5, 135.4, 130.3, 129.9, 129.2, 129.1, 128.8, 128.7, 126.4, 126.0, 41.2 (*para*-isomer), 39.1 (*ortho*-isomer), 21.1, 19.7 (*ortho*-isomer). The spectral data is in accordance with previous reports.[19]

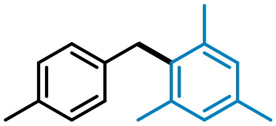

1,3,5-trimethyl-2-(4-methylbenzyl)benzene (2l); Prepared using method **GP4** and obtained as a yellow oil, mass = 136 mg, 60% yield; **^{1}H NMR** (400 MHz, $CDCl_3$) δ 7.07 (d, *J* = 7.8 Hz, 2H), 6.95 – 6.90 (m, 4H), 4.01 (s, 2H), 2.32 (s, 6H), 2.23 (s, 6H). **^{13}C{^{1}H} NMR** (101 MHz, $CDCl_3$) δ 137.14, 137.09, 135.7, 135.2, 134.1, 129.2, 129.0, 127.9, 34.4, 21.1, 21.0, 20.3. The spectral data is in accordance with previous reports.[5]

<u>Characterization of Friedel-Crafts products from aryl ketones</u>

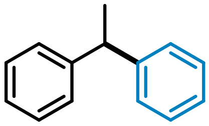

Ethane-1,1-diyldibenzene (3a); Prepared using method **GP5** and obtained as a colorless oil, mass = 178 mg, 93% yield; The spectral data is identical to **1h**.

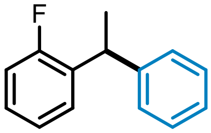

1-fluoro-2-(1-phenylethyl)benzene (3b); Prepared using method **GP5** and obtained as a colorless oil, mass = 159 mg, 75% yield; ^{1}H NMR (400 MHz, $CDCl_3$) δ 7.41 – 7.16 (m, 7H), 7.13 – 7.08 (m, 1H), 7.06 – 6.99 (m, 1H), 4.52 (q, *J* = 7.4 Hz, 1H), 1.68 (d, *J* = 7.3 Hz, 3H). **^{13}C{^{1}H} NMR** (101 MHz, $CDCl_3$) δ 160.7 (d, *J* = 245.8 Hz), 145.2, 133.4 (d, *J* = 14.5 Hz), 128.6 (d, *J* = 4.6 Hz), 128.5, 127.8 (d, *J* = 8.3 Hz), 127.7, 126.3, 124.2 (d, *J* = 3.6 Hz), 115.5 (d, *J* = 22.5 Hz), 37.7, 20.9. **^{19}F NMR** (376 MHz, $CDCl_3$) δ -119.3. The spectral data is in accordance with previous reports.[20]

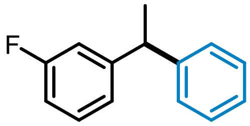

1-fluoro-3-(1-phenylethyl)benzene (3c); Prepared using method **GP5** and obtained as a yellow oil, mass = 142 mg, 71% yield; **^{1}H NMR** (400 MHz, $CDCl_3$) δ 7.41 – 7.35 (m, 2H), 7.34 – 7.25 (m, 4H), 7.11 – 7.06 (m, 1H), 7.02 (d, *J* = 10.2 Hz, 1H), 6.96 (t, *J* = 7.7 Hz, 1H), 4.23 (q, *J* = 7.3 Hz, 1H), 1.72 (d, *J* = 7.3 Hz, 3H). **^{13}C{^{1}H} NMR** (101 MHz, $CDCl_3$) δ 163.1 (d, *J* = 245.5 Hz), 149.2 (d, *J* = 6.7 Hz), 145.7, 129.8 (d, *J* = 8.3 Hz), 128.6, 127.7, 126.4, 123.4 (d, *J* = 2.8 Hz), 114.6 (d, *J* = 21.2 Hz), 113.0 (d, *J* = 21.1 Hz), 44.7, 21.8. **^{19}F NMR** (376 MHz, $CDCl_3$) δ -114.7. The spectral data is in accordance with previous reports.[6]

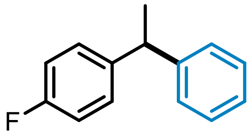

1-fluoro-4-(1-phenylethyl)benzene (3d); Prepared using method **GP5** and obtained as a colorless oil, mass = 151 mg, 76% yield; **^{1}H NMR** (400 MHz, $CDCl_3$) δ 7.38 – 7.30 (m, 2H), 7.28 – 7.18 (m, 5H), 7.05 – 6.96 (m, 2H), 4.19 (q, *J* = 7.3 Hz, 1H), 1.67 (d, *J* = 7.3 Hz, 3H). **^{13}C{^{1}H} NMR** (101 MHz, $CDCl_3$) δ 161.4 (d, *J* = 244.0 Hz), 146.3, 142.2, 129.1 (d, *J* = 7.8 Hz), 128.6, 127.7, 126.3, 115.2 (d, *J* = 21.1 Hz), 44.2, 22.1. **^{19}F NMR** (376 MHz, $CDCl_3$) δ -118.9. The spectral data is in accordance with previous reports.[21]

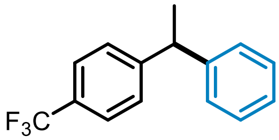

1-(1-phenylethyl)-4-(trifluoromethyl)benzene (3e); Prepared using method **GP5** and obtained as a yellow oil, mass = 211 mg, 88% yield; **^{1}H NMR** (400 MHz, $CDCl_3$) δ 7.38 – 7.30 (m, 2H), 7.28 – 7.18 (m, 5H), 7.05 – 6.96 (m, 2H), 4.19 (q, *J* = 7.3 Hz, 1H), 1.67 (d, *J* = 7.3 Hz, 3H). **^{13}C{^{1}H} NMR** (101 MHz, $CDCl_3$) δ 150.6, 145.4, 128.7, 128.1, 127.7, 126.6, 125.5 (q, *J* = 3.8 Hz), 125.4, 44.8, 21.7. **^{19}F NMR** (376 MHz, $CDCl_3$) δ -63.8. The spectral data is in accordance with previous reports.[22]

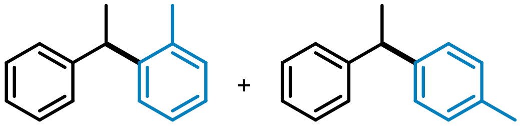

1-methyl-2-(1-phenylethyl)benzene + 1-methyl-4-(1-phenylethyl)benzene (3f); Prepared using method **GP5** and obtained as a colorless oil, mass = 165 mg, 84% yield; **^{1}H NMR** (400 MHz, $CDCl_3$) δ 7.32 (t, *J* = 7.4 Hz, 2H), 7.29 – 7.24 (m, 2H), 7.21 (t, *J* = 6.9 Hz, 1H), 7.15 (t, *J* = 5.9 Hz, 4H), 4.16 (q, *J* = 7.2 Hz, 1H), 2.35 (s, 3H), 1.67 (d, *J* = 7.2 Hz, 3H). **^{13}C{^{1}H} NMR** (101 MHz, $CDCl_3$) δ 146.6, 143.4, 135.5, 129.1, 128.4, 127.6, 127.5, 126.0, 44.4, 22.0, 21.0. The spectral data is in accordance with previous reports.[22]

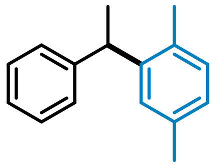

1,4-dimethyl-2-(1-phenylethyl)benzene (3g); Prepared using method **GP5** and obtained as a colorless oil, mass = 214 mg, 99% yield; **^{1}H NMR** (400 MHz, $CDCl_3$) δ 7.35 – 7.28 (m, 2H), 7.26 – 7.19 (m, 3H), 7.17 – 7.12 (m, 1H), 7.11 – 7.06 (m, 1H), 7.04 – 6.98 (m, 1H), 4.36 (q, *J* = 7.2 Hz, 1H), 2.39 (s, 3H), 2.26 (s, 3H), 1.68 (d, *J* = 7.2 Hz, 3H). **^{13}C{^{1}H} NMR** (101 MHz, $CDCl_3$) δ 146.5, 143.8, 135.4, 133.0, 130.4, 128.4, 127.8, 127.6, 126.9, 125.9, 41.1, 22.2, 21.4, 19.4. The spectral data is in accordance with previous reports.[23]

Characterization of Friedel-Crafts products from aryl acids

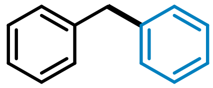

Diphenylmethane (4a); Prepared using method **GP6** and obtained as a pale yellow oil, mass = 94 mg, 56% yield; The spectral data is identical to **1a**.

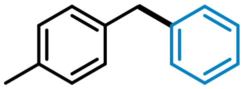

1-benzyl-4-methylbenzene (4b); Prepared using method **GP6** and obtained as a colorless oil, mass = 80 mg, 44% yield; The spectral data is identical to **1b**.

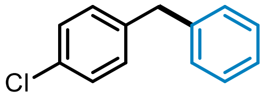

1-benzyl-4-chlorobenzene (4c); Prepared using method **GP6** and obtained as a colorless oil, mass = 152 mg, 75% yield; The spectral data is identical to **1d**.

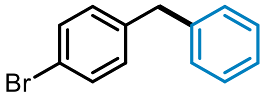

1-benzyl-4-bromobenzene (4d); Prepared using method **GP6** and obtained as a colorless oil, mass = 181 mg, 73% yield; **^{1}H NMR** (400 MHz, $CDCl_3$) δ 7.46 (d, *J* = 8.4 Hz, 2H), 7.35 (d, *J* = 6.1 Hz, 2H), 7.28 (t, *J* = 5.0 Hz, 1H), 7.23 (d, *J* = 7.3 Hz, 2H), 7.12 (d, *J* = 8.4 Hz, 2H), 3.99 (s, 2H). **^{13}C{^{1}H} NMR** (101 MHz, $CDCl_3$) δ 140.6, 140.2, 131.6, 130.8, 129.0, 128.7, 126.4, 120.1, 41.4. The spectral data is in accordance with previous reports.[1]

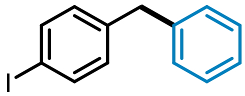

1-benzyl-4-iodobenzene (4e); Prepared using method **GP6** and obtained as a pale yellow oil, mass = 170 mg, 58% yield; **^{1}H NMR** (400 MHz, $CDCl_3$) δ 7.67 – 7.60 (m, 2H), 7.37-7.30 (m, 2H), 7.29 – 7.24 (m, 1H), 7.23 – 7.17 (m, 2H), 7.00 – 6.94 (m, 2H), 3.97 (s, 2H). **^{13}C{^{1}H} NMR** (101 MHz, $CDCl_3$) δ 140.9, 140.5, 137.6, 131.1, 129.0, 128.7, 126.4, 91.4, 41.5. The spectral data is in accordance with previous reports.[1]

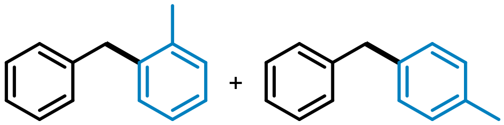

1-benzyl-2-methylbenzene + **1-benzyl-4-methylbenzene (4f);** Prepared using method **GP6** and obtained as a colorless oil, mass = 111 mg, 61% yield; The spectral data is identical to **1e**.

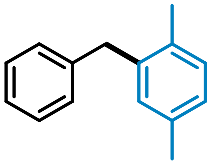

2-benzyl-1,4-dimethylbenzene (4g); Prepared using method **GP6** and obtained as a pale yellow oil, mass = 86 mg, 43% yield; The spectral data is identical to **1f**.

Characterization of Friedel-Crafts products from aryl esters

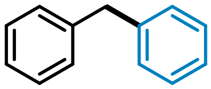

Diphenylmethane (5a); Prepared using method **GP7** and obtained as a pale yellow oil, mass = 134 mg, 78% yield; The spectral data is identical to **1a**.

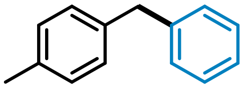

1-benzyl-4-methylbenzene (5b); Prepared using method **GP** and obtained as a colorless oil, mass = 88 mg, 47% yield; The spectral data is identical to **1b**.

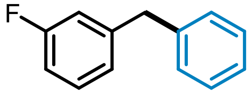

1-benzyl-3-fluorobenzene (5c); Prepared using method **GP7** (20 h, then 1 equiv. $TiCl_4$ for 2 h) and obtained as a colorless oil, mass = 150 mg, 79% yield; **^{1}H NMR** (400 MHz, $CDCl_3$) δ 7.35 – 7.29 (m, 2H), 7.29 – 7.23 (m, 2H), 7.23 – 7.18 (m, 2H), 7.02 – 6.97 (m, 1H), 6.94 – 6.87 (m, 2H), 3.99 (s, 2H). **^{13}C{^{1}H} NMR** (101 MHz, $CDCl_3$) δ 163.1 (d, *J* = 245.7 Hz), 143.8 (d, *J* = 7.4 Hz), 140.4, 130.0 (d, *J* = 8.3 Hz), 129.1, 128.7, 126.5, 124.7 (d, *J* = 2.9 Hz), 115.9 (d, *J* = 21.2 Hz), 113.1 (d, *J* = 20.9 Hz), 41.8. **^{19}F NMR** (376 MHz, $CDCl_3$) δ -115.0. The spectral data is in accordance with previous reports.[3]

References

(1) He, T.; Klare, H. F. T.; Oestreich, M., Catalytically Generated Meerwein's Salt-Type Oxonium Ions for Friedel-Crafts C(sp(2))-H Methylation with Methanol. *J Am Chem Soc* **2023**, *145* (6), 3795-3801.

(2) Wang, X. X.; Luo, M. J.; Lu, J. M., N-Heterocyclic carbene-palladium(ii)-1-methylimidazole complex-catalyzed Suzuki-Miyaura coupling of benzyl carbamates with arylboronic acids. *Org Biomol Chem* **2015**, *13* (47), 11438-44.

(3) Tang, J.; Lv, L.; Dai, X. J.; Li, C. C.; Li, L.; Li, C. J., Nickel-catalyzed cross-coupling of aldehydes with aryl halides via hydrazone intermediates. *Chem Commun (Camb)* **2018**, *54* (14), 1750-1753.

(4) Li, Y.; Xiong, Y.; Li, X.; Ling, X.; Huang, R.; Zhang, X.; Yang, J., Benzylation of arenes with benzyl ethers promoted by the in situ prepared superacid BF3–H2O. *Green Chemistry* **2014**, *16* (6).

(5) Lv, F.; Xiao, J.; Xiang, J.; Guo, F.; Tang, Z. L.; Han, L. B., Conversion of Aryl Aldehydes to Benzyl Iodides and Diarylmethanes by H(3)PO(3)/I(2). *J Org Chem* **2021**, *86* (3), 3081-3088.

(6) Mahajani, N. S.; Chisholm, J. D., Synthesis of 1,1'-Diarylethanes and Related Systems by Displacement of Trichloroacetimidates with Trimethylaluminum. *J Org Chem* **2018**, *83* (7), 4131-4139.

(7) Han, B.; Ren, C.; Wu, L., Titanium-Catalyzed Hydrodehalogenation of Alkyl Halides. *Organometallics* **2023**, *42* (12), 1248-1253.

(8) Chatterjee, I.; Qu, Z. W.; Grimme, S.; Oestreich, M., B(C6F5)3 -catalyzed transfer of dihydrogen from one unsaturated hydrocarbon to another. *Angew Chem Int Ed Engl* **2015**, *54* (41), 12158-62.

(9) Donck, S.; Baroudi, A.; Fensterbank, L.; Goddard, J.-P.; Ollivier, C., Visible-Light Photocatalytic Reduction of Sulfonium Salts as a Source of Aryl Radicals. *Adv. Synth. Catal.* **2013**, *355* (8), 1477-1482.

(10) Prakash, G. K. S.; Panja, C.; Shakhmin, A.; Shah, E.; Mathew, T.; Olah, G. A., BF3−H2O Catalyzed Hydroxyalkylation of Aromatics with Aromatic Aldehydes and Dicarboxaldehydes: Efficient Synthesis of Triarylmethanes, Diarylmethylbenzaldehydes, and Anthracene Derivatives. *J. Org. Chem.* **2009**, *74* (22), 8659-8668.

(11) Zhang, Z.; Wang, H.; Qiu, N.; Kong, Y.; Zeng, W.; Zhang, Y.; Zhao, J., Synthesis of Triarylmethanes via Palladium-Catalyzed Suzuki Coupling of Trimethylammonium Salts and Arylboronic Acids. *J. Org. Chem.* **2018**, *83* (15), 8710-8715.

(12) Yuan, F.-Q.; Gao, L.-X.; Han, F.-S., PdCl2-catalyzed efficient allylation and benzylation of heteroarenes under ligand, base/acid, and additive-free conditions. *Chem. Comm.* **2011**, *47* (18), 5289-5291.

(13) Xia, Y.; Hu, F.; Liu, Z.; Qu, P.; Ge, R.; Ma, C.; Zhang, Y.; Wang, J., Palladium-Catalyzed Diarylmethyl C(sp3)–C(sp2) Bond Formation: A New Coupling Approach toward Triarylmethanes. *Org. Lett.* **2013**, *15* (7), 1784-1787.

(14) Bonsir, M.; Davila, C.; Kennedy, A. R.; Geerts, Y., Exhaustive One-Step Bridgehead Methylation of Adamantane Derivatives with Tetramethylsilane. *European Journal of Organic Chemistry* **2021**, *2021* (37), 5227-5237.

(15) Yang, X. W.; Li, D. H.; Song, A. X.; Liu, F. S., "Bulky-Yet-Flexible" alpha-Diimine Palladium-Catalyzed Reductive Heck Cross-Coupling: Highly Anti-Markovnikov-Selective Hydroarylation of Alkene in Air. *J Org Chem* **2020**, *85* (18), 11750-11765.

(16) Zhang, S.; Vayer, M.; Noël, F.; Vuković, V. D.; Golushko, A.; Rezajooei, N.; Rowley, C. N.; Lebœuf, D.; Moran, J., Unlocking the Friedel-Crafts arylation of primary aliphatic alcohols and epoxides driven by hexafluoroisopropanol. *Chem* **2021**, *7* (12), 3425-3441.

(17) Dan, X.; Yang, Q.; Xing, L.; Tang, Y.; Wang, W.; Cai, Y., Heterogeneous Metallaphotocatalytic C(sp(2))-C(sp(3)) Cross-Coupling Reactions with Integrated Bipyridyl-Ni(II)-Carbon Nitride. *Org Lett* **2023**, *25* (22), 4124-4129.

(18) Ishida, N.; Masuda, Y.; Ishikawa, N.; Murakami, M., Cooperation of a Nickel–Bipyridine Complex with Light for Benzylic C−H Arylation of Toluene Derivatives. *Asian Journal of Organic Chemistry* **2017**, *6* (6), 669-672.

(19) Fukuzawa, S.-i.; Tsuchimoto, T.; Hiyama, T., Superacid-Catalyzed Reductive Friedel−Crafts Reaction of Arenes Using Arenecarbaldehyde Acetals. *J. Org. Chem.* **1997**, *62* (1), 151-156.

(20) Chen, J.; Chen, C.; Ji, C.; Lu, Z., Cobalt-Catalyzed Asymmetric Hydrogenation of 1,1-Diarylethenes. *Org. Lett.* **2016**, *18* (7), 1594-1597.

(21) Peng, L.; Li, Z.; Yin, G., Photochemical Nickel-Catalyzed Reductive Migratory Cross-Coupling of Alkyl Bromides with Aryl Bromides. *Org Lett* **2018**, *20* (7), 1880-1883.

(22) Kumar, G.; Bhattacharya, D.; Mistry, P.; Chatterjee, I., In-Catalyzed Transfer Hydrogenation and Regioselective Hydrogen-Deuterium Addition to the Olefins. *J Org Chem* **2023**, *88* (11), 6987-6994.

(23) Dayal, N.; Opoku-Temeng, C.; Mohammad, H.; Abutaleb, N. S.; Hernandez, D.; Onyedibe, K. I.; Wang, M.; Zeller, M.; Seleem, M. N.; Sintim, H. O., Inhibitors of Intracellular Gram-Positive Bacterial Growth Synthesized via Povarov-Doebner Reactions. *ACS Infect. Dis* **2019**, *5* (11), 1820-1830.

NMR Spectra

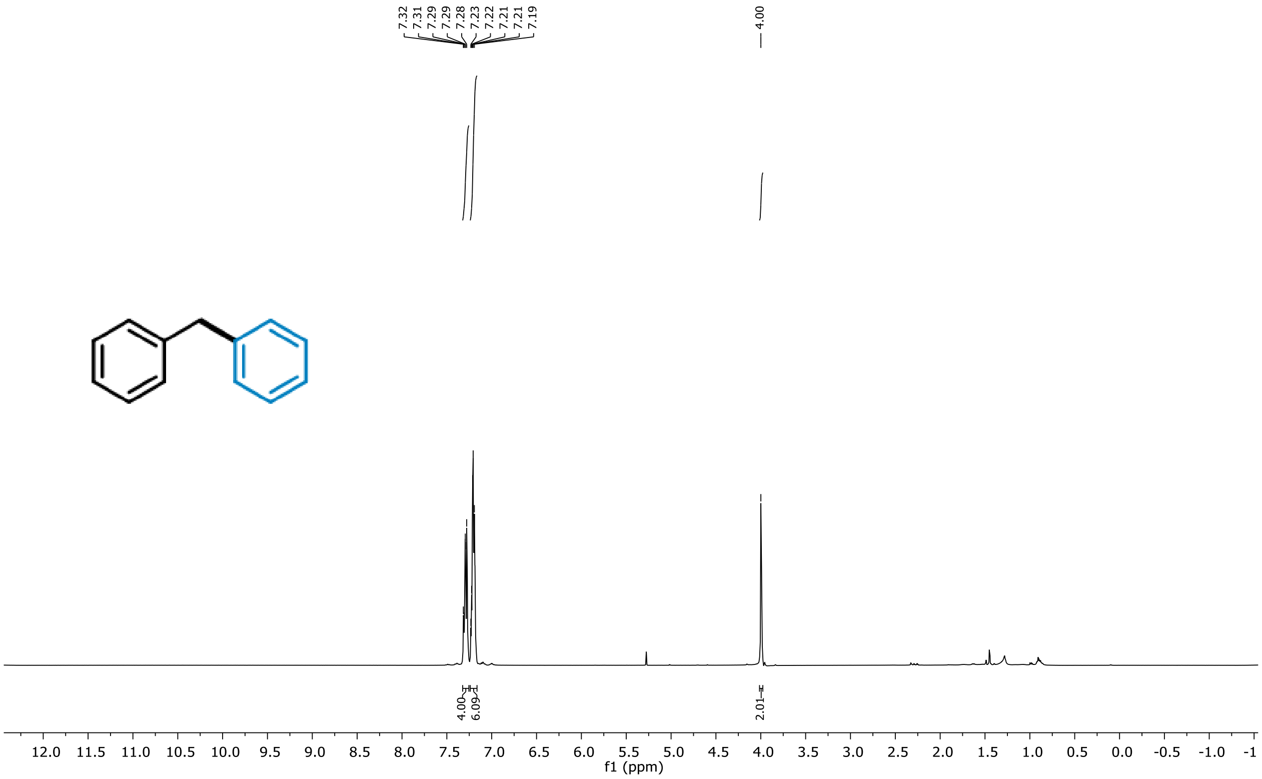

^{1}H NMR (400 MHz, $CDCl_3$) diphenylmethane (**1a**)

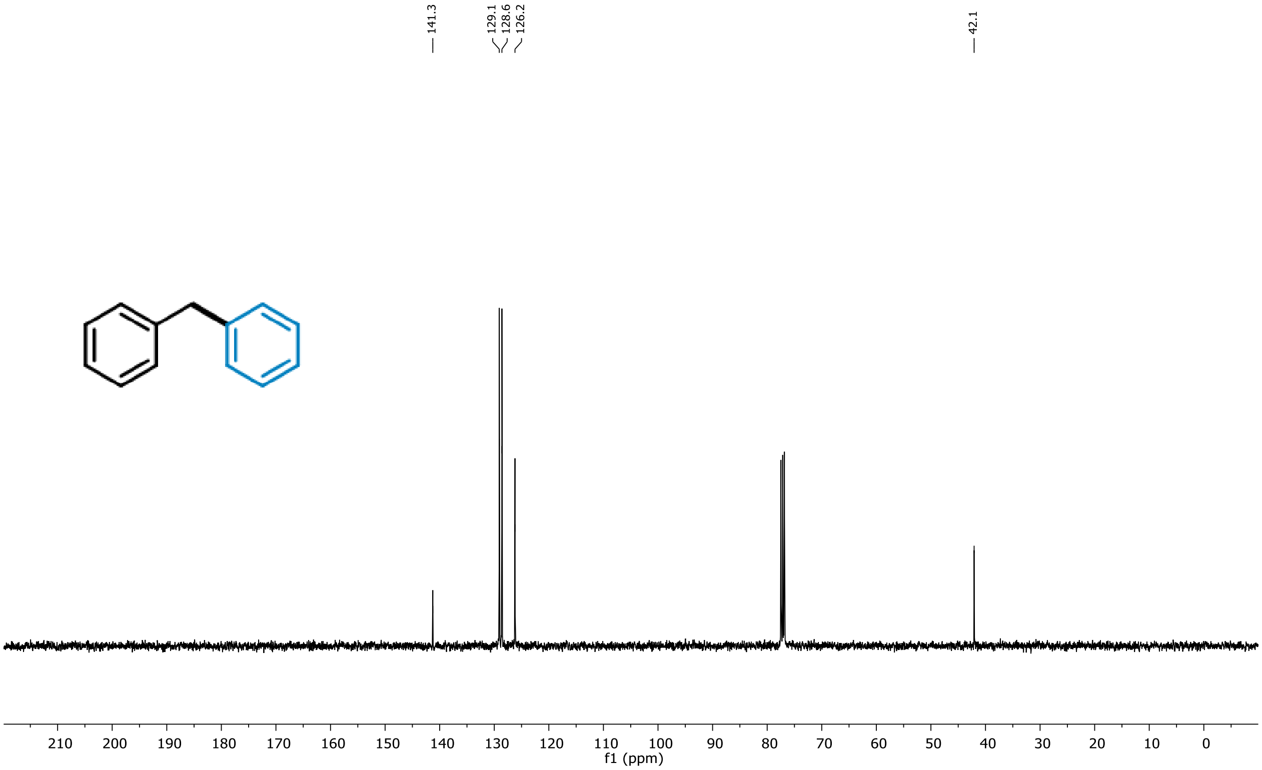

^{13}C{^{1}H} NMR (101 MHz, $CDCl_3$) diphenylmethane (**1a**)

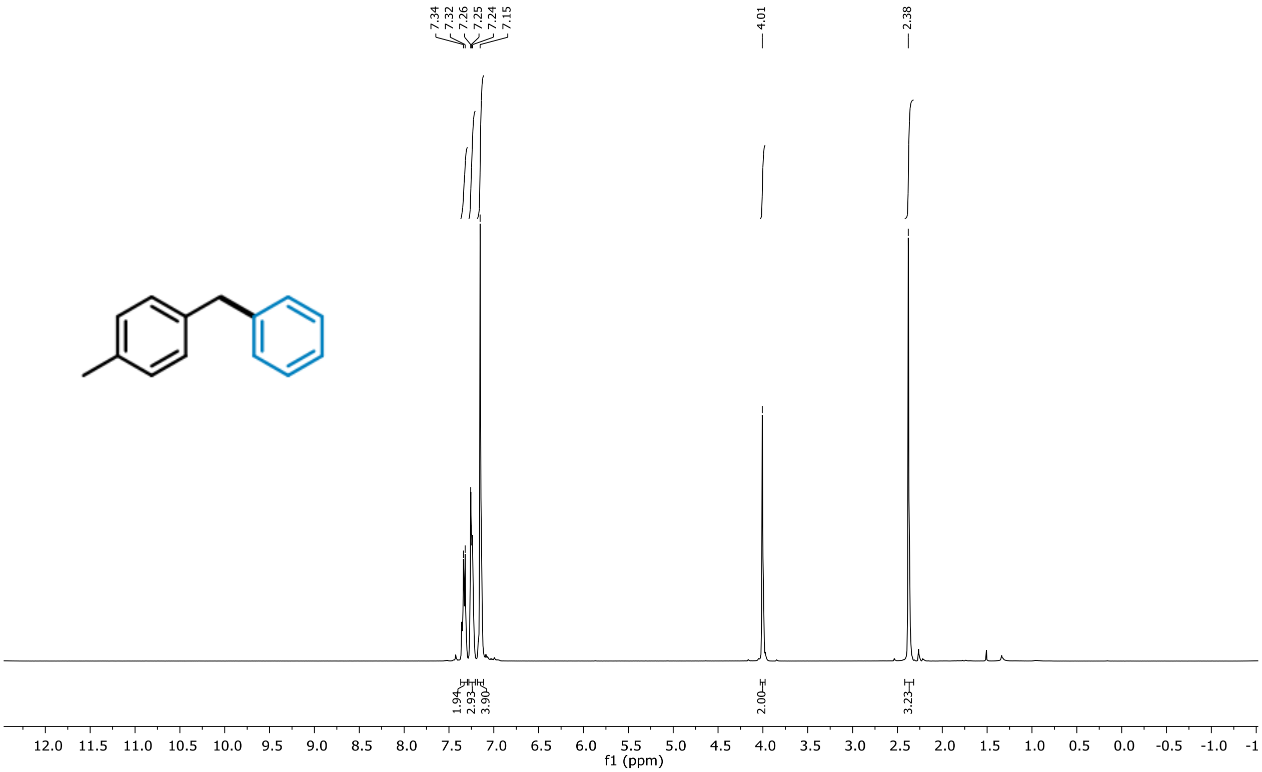

^{1}H NMR (400 MHz, $CDCl_3$) 1-benzyl-4-methylbenzene (**1b**)

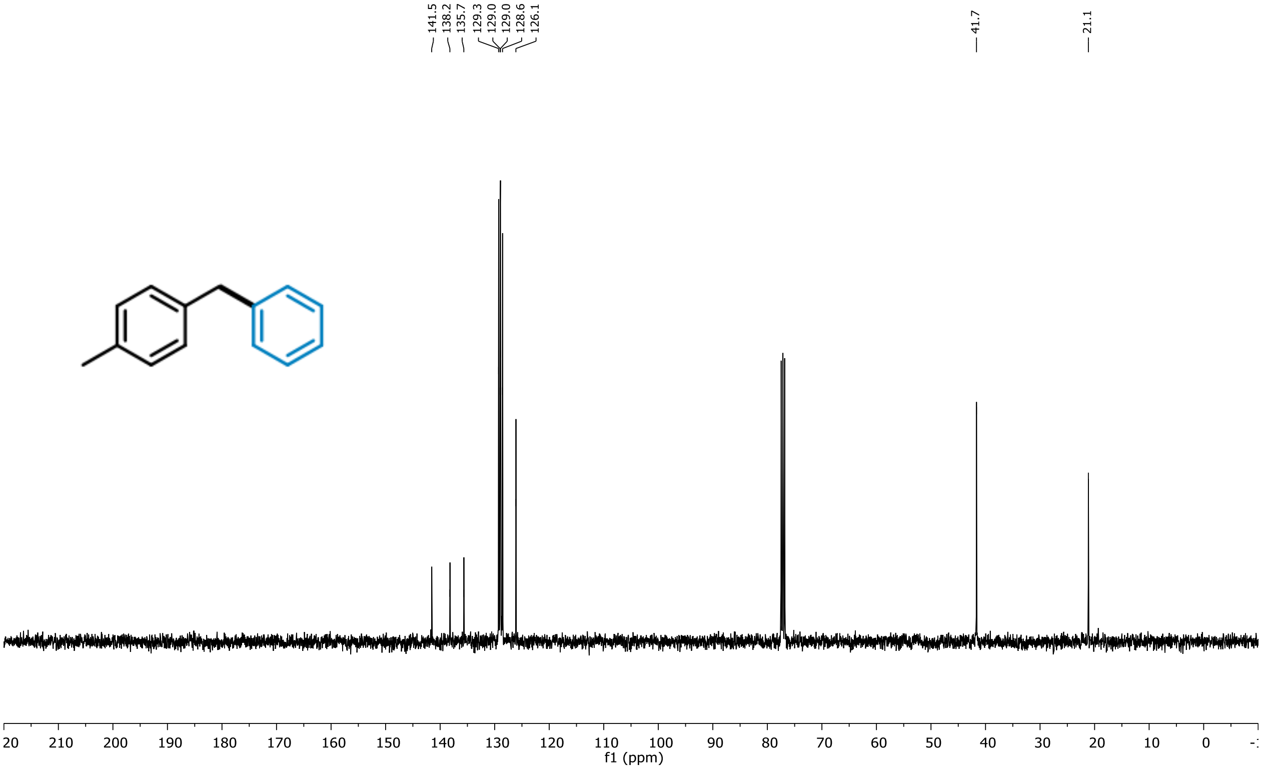

$^{13}C\{^{1}H\}$ NMR (101 MHz, $CDCl_3$) 1-benzyl-4-methylbenzene (**1b**)

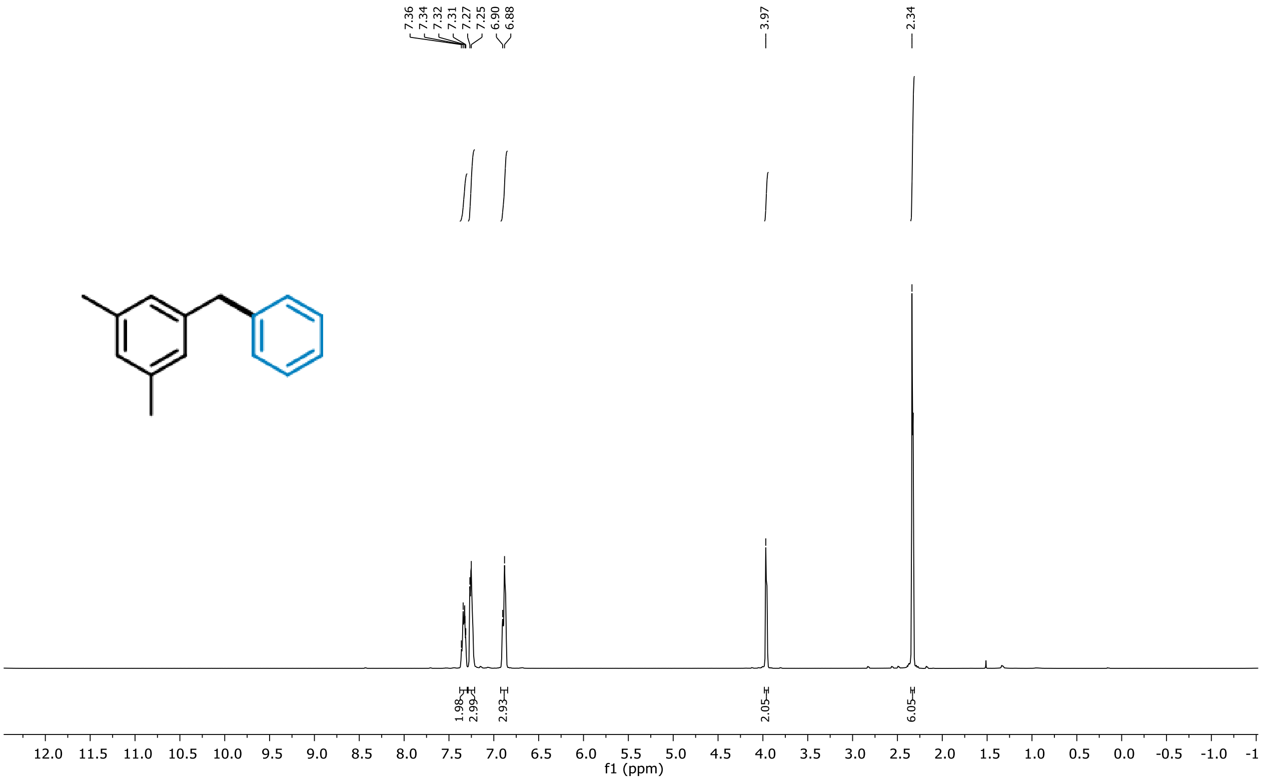

^{1}H NMR (400 MHz, $CDCl_3$) 1-benzyl-3,5-dimethylbenzene (**1c**)

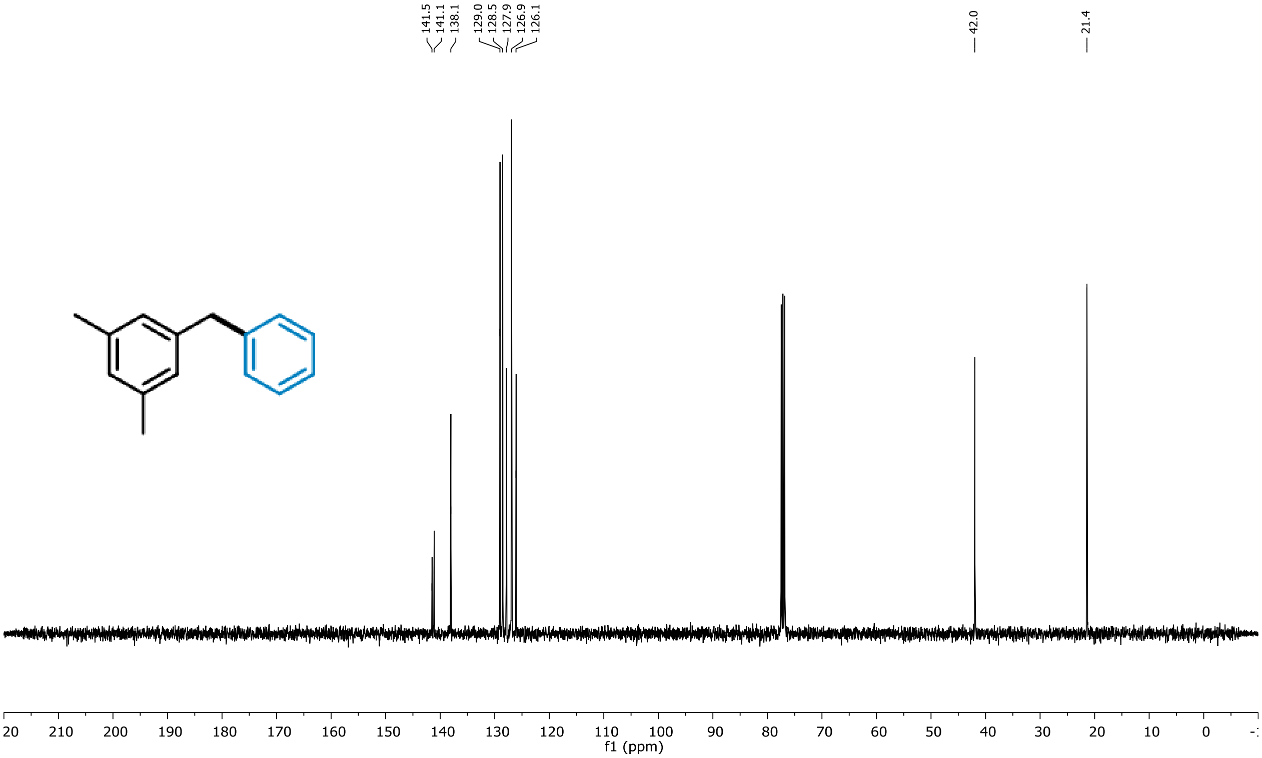

$^{13}C\{^{1}H\}$ NMR (101 MHz, $CDCl_3$) 1-benzyl-3,5-dimethylbenzene (**1c**)

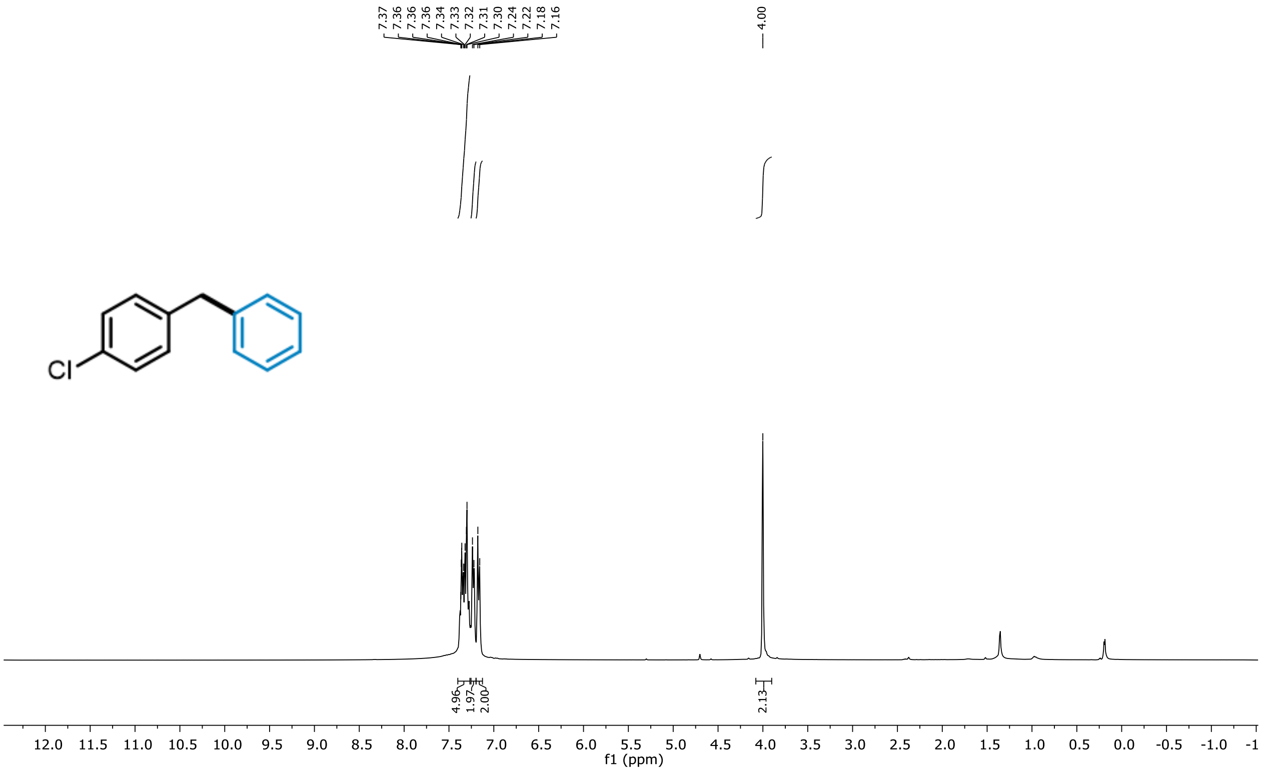

^{1}H NMR (400 MHz, $CDCl_3$) 1-benzyl-4-chlorobenzene (**1d**)

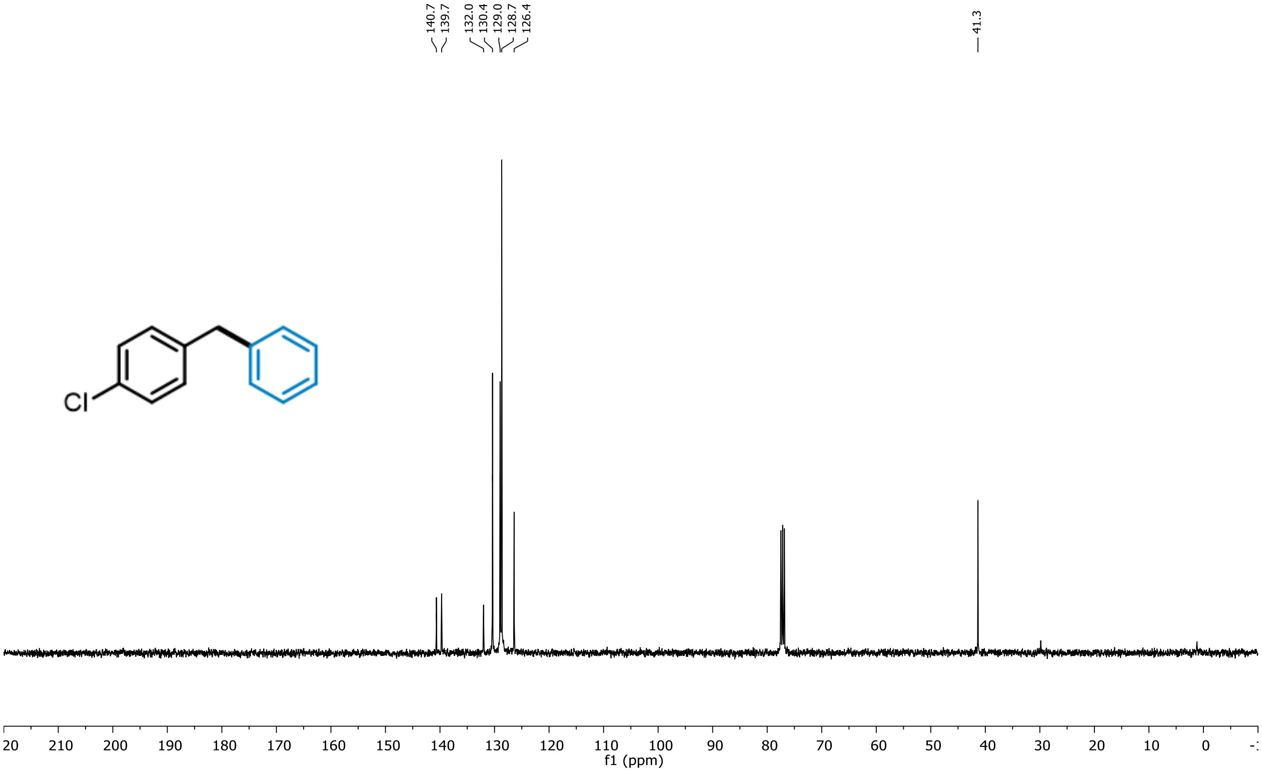

$^{13}C\{^{1}H\}$ NMR (101 MHz, $CDCl_3$) 1-benzyl-4-chlorobenzene (**1d**)

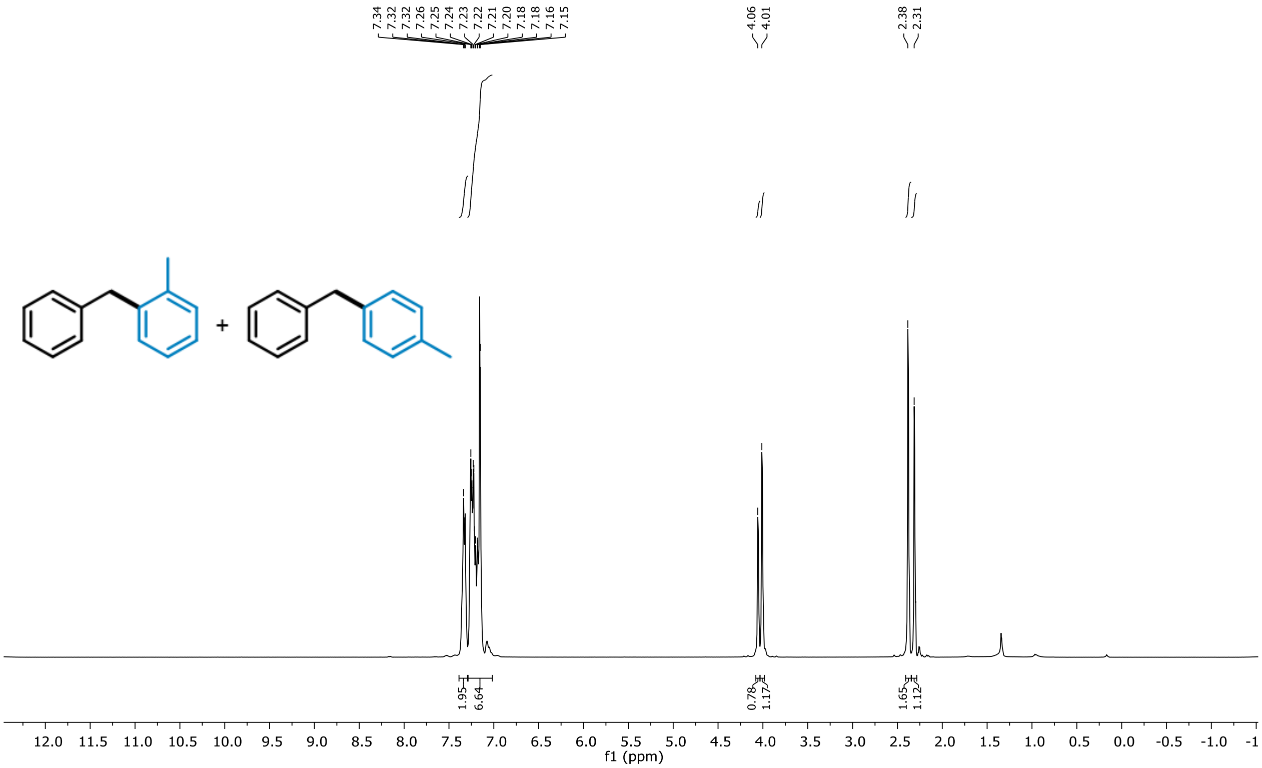

^{1}H NMR (400 MHz, $CDCl_3$) 1-benzyl-2-methylbenzene + 1-benzyl-4-methylbenzene (**1e**)

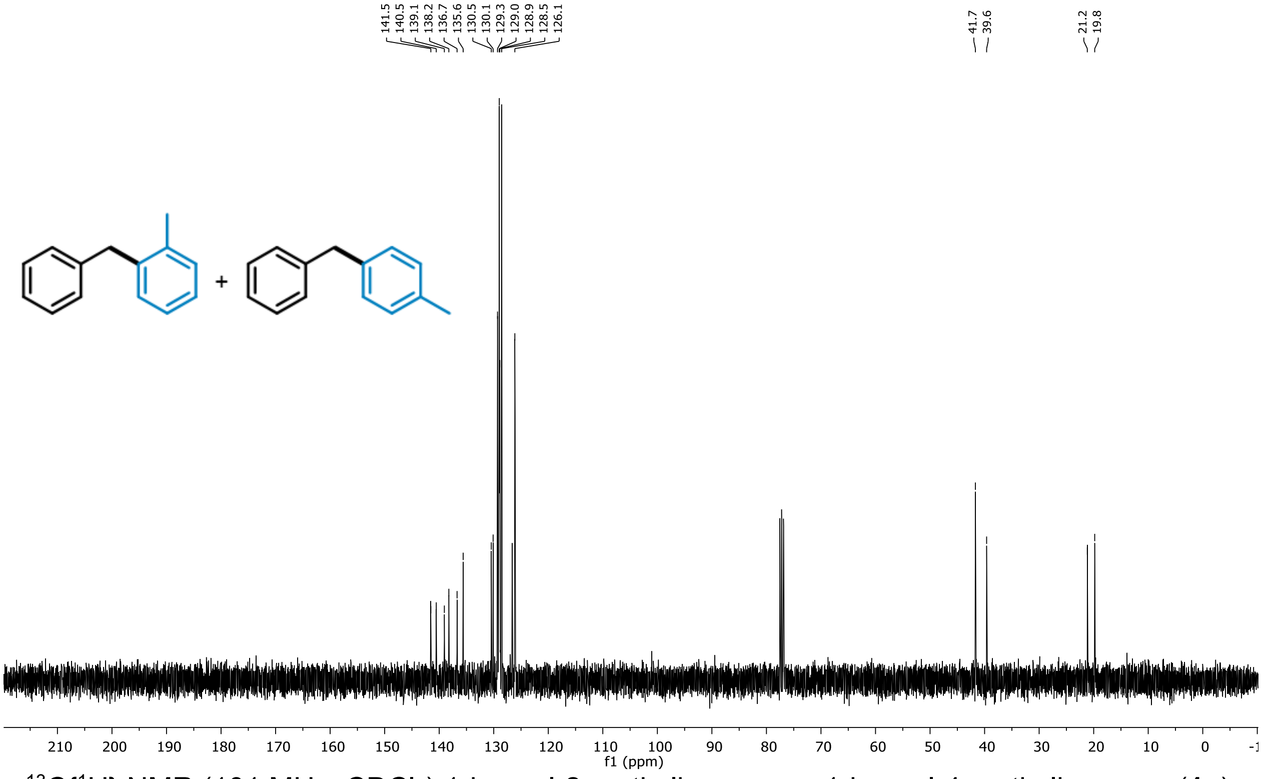

$^{13}C\{^{1}H\}$ NMR (101 MHz, $CDCl_3$) 1-benzyl-2-methylbenzene + 1-benzyl-4-methylbenzene (**1e**)

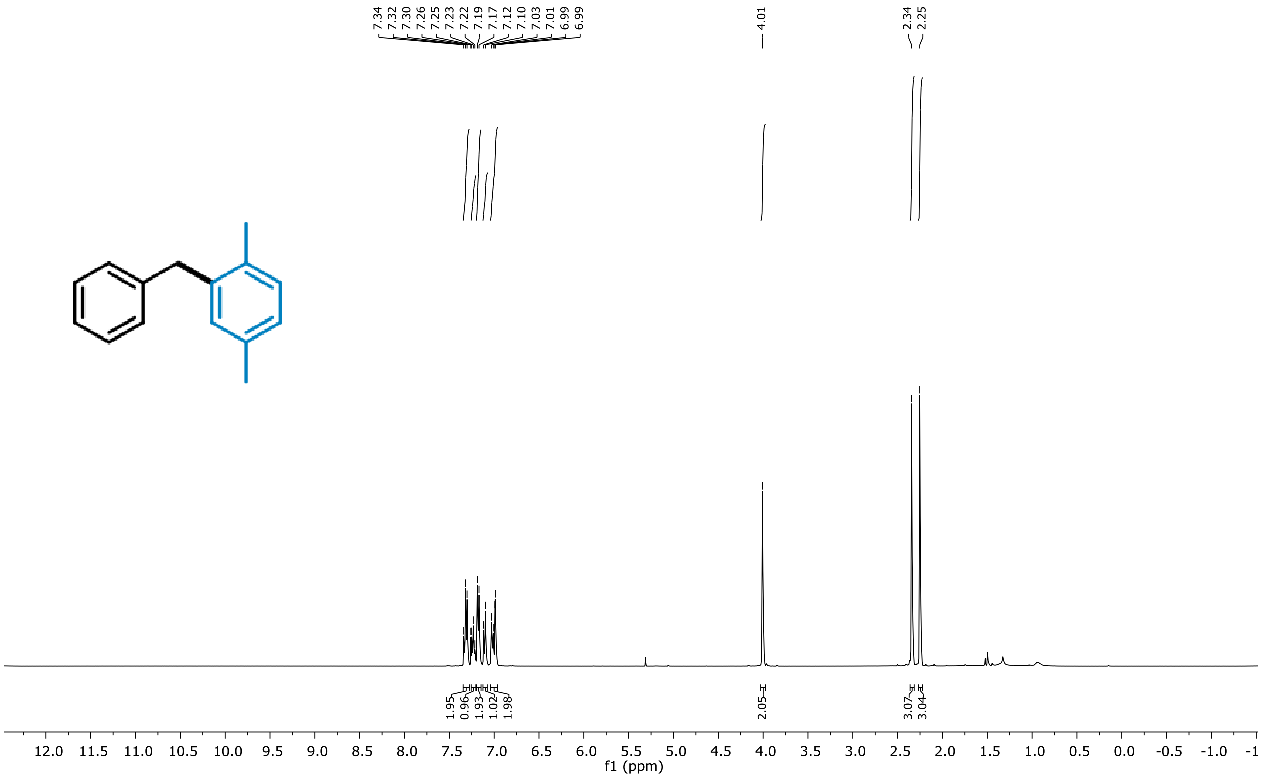

^{1}H NMR (400 MHz, $CDCl_3$) 2-benzyl-1,4-dimethylbenzene (**1f**)

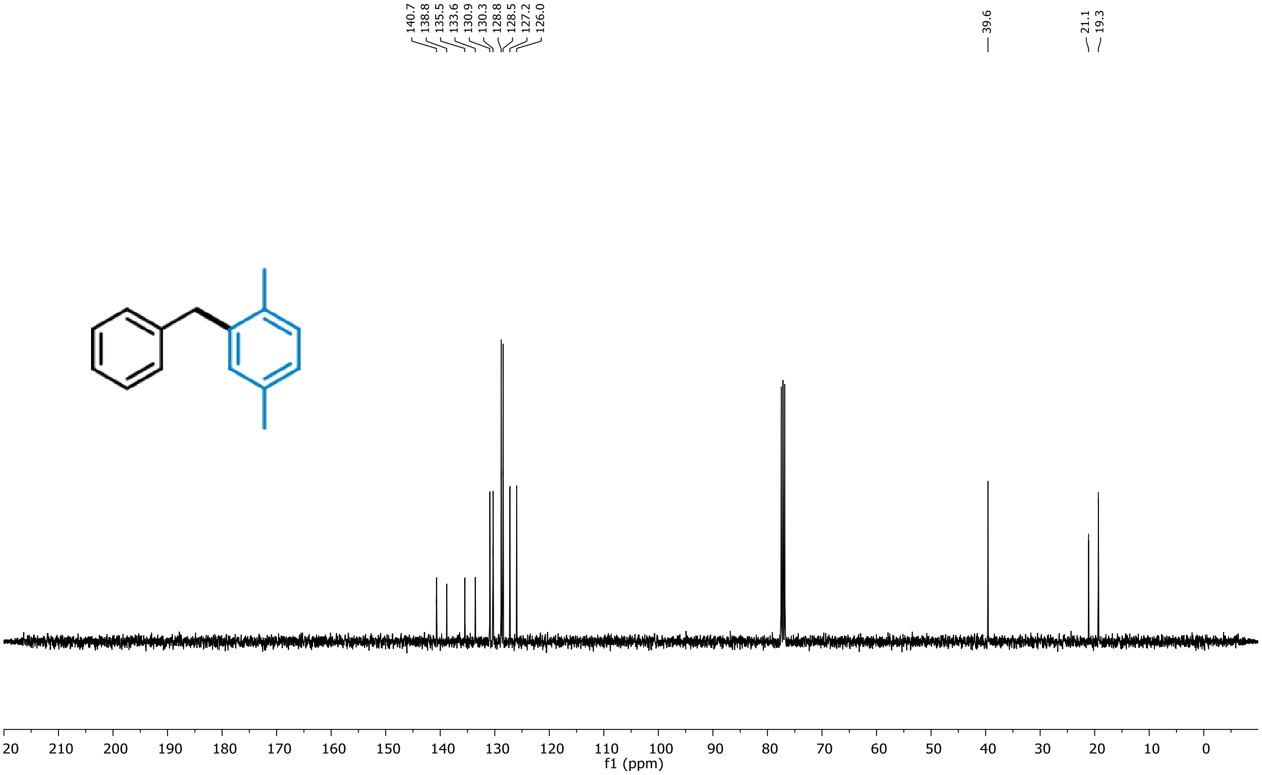

$^{13}C\{^1H\}$ NMR (101 MHz, $CDCl_3$) 2-benzyl-1,4-dimethylbenzene (**1f**)

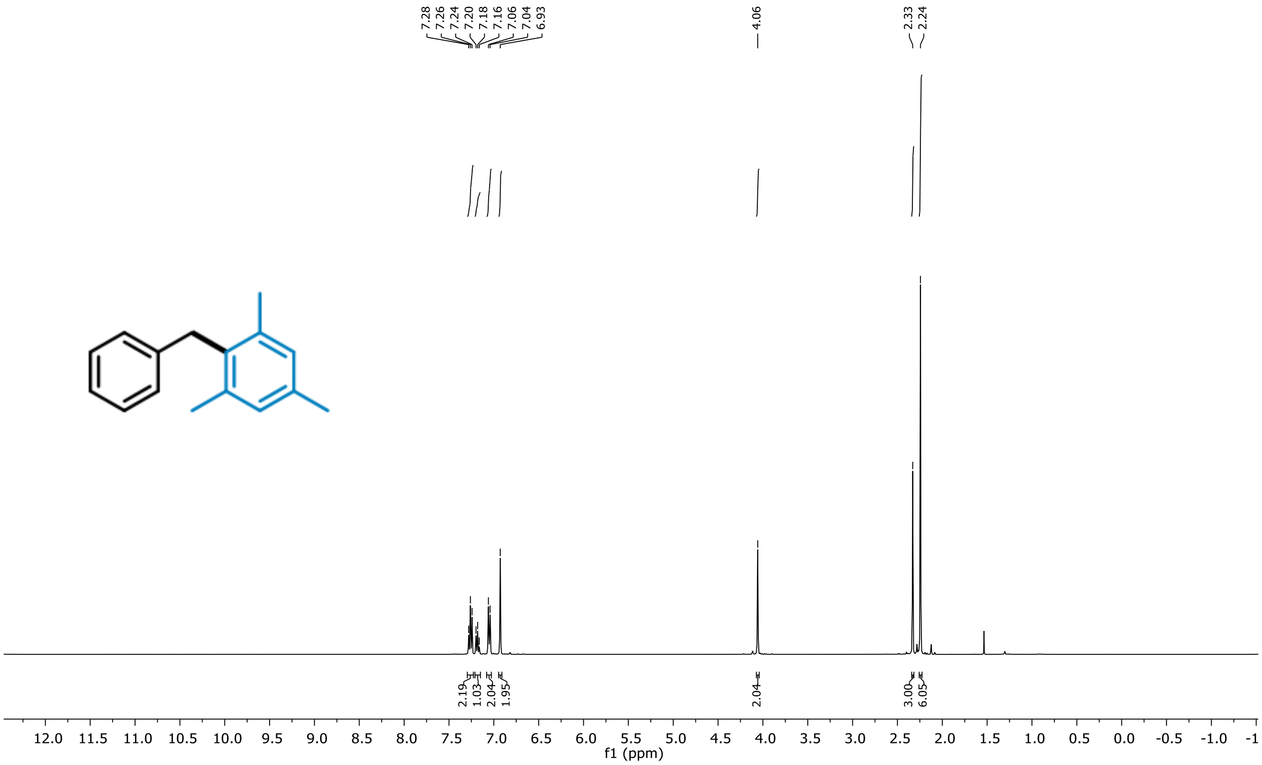

^{1}H NMR (400 MHz, $CDCl_3$) 2-benzyl-1,3,5-trimethylbenzene (**1g**)

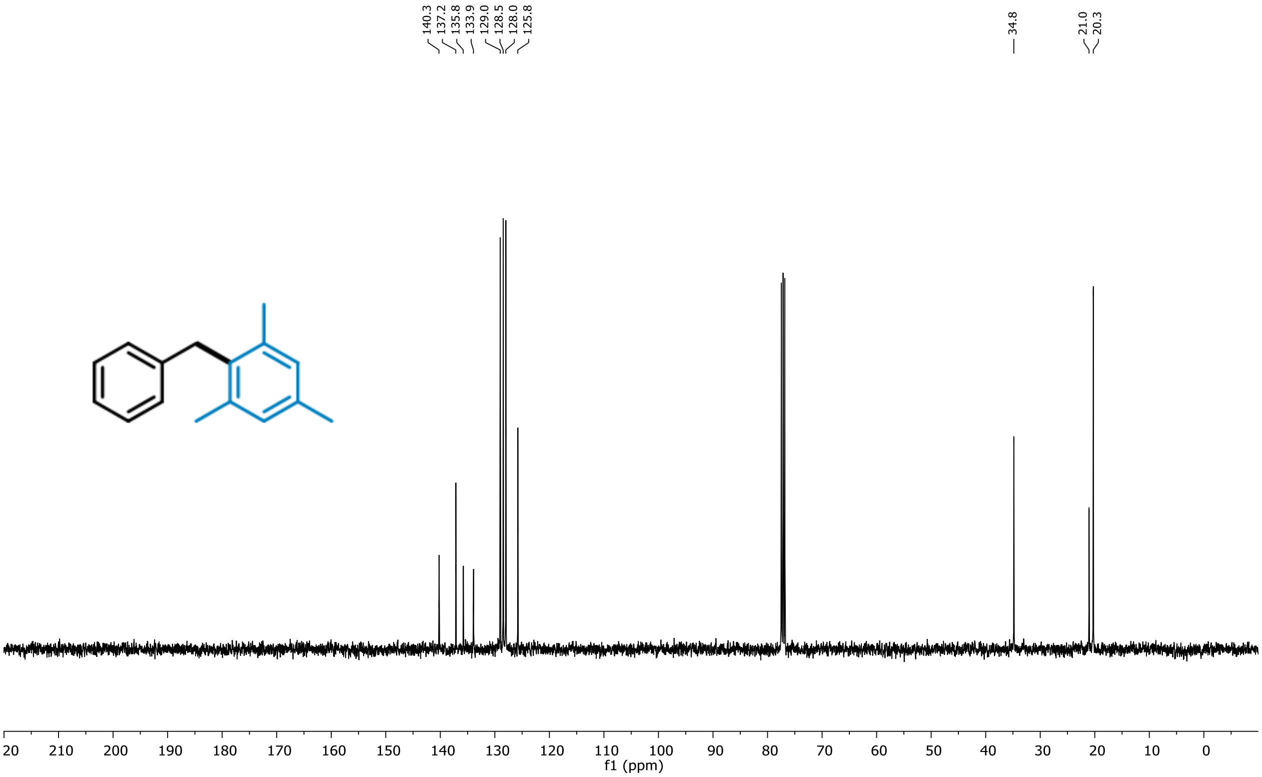

^{13}C{^{1}H} NMR (101 MHz, $CDCl_3$) 2-benzyl-1,3,5-trimethylbenzene (**1g**)

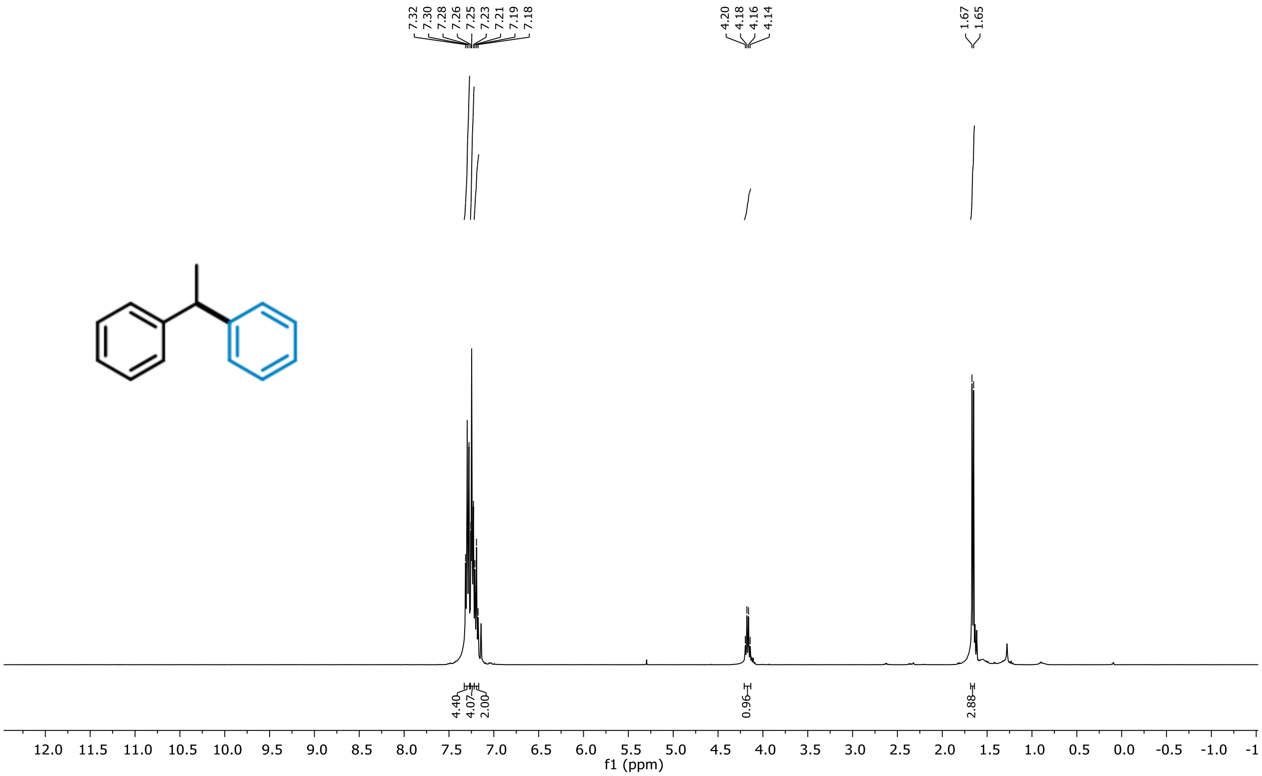

^{1}H NMR (400 MHz, $CDCl_3$) ethane-1,1-diyldibenzene (**1h**)

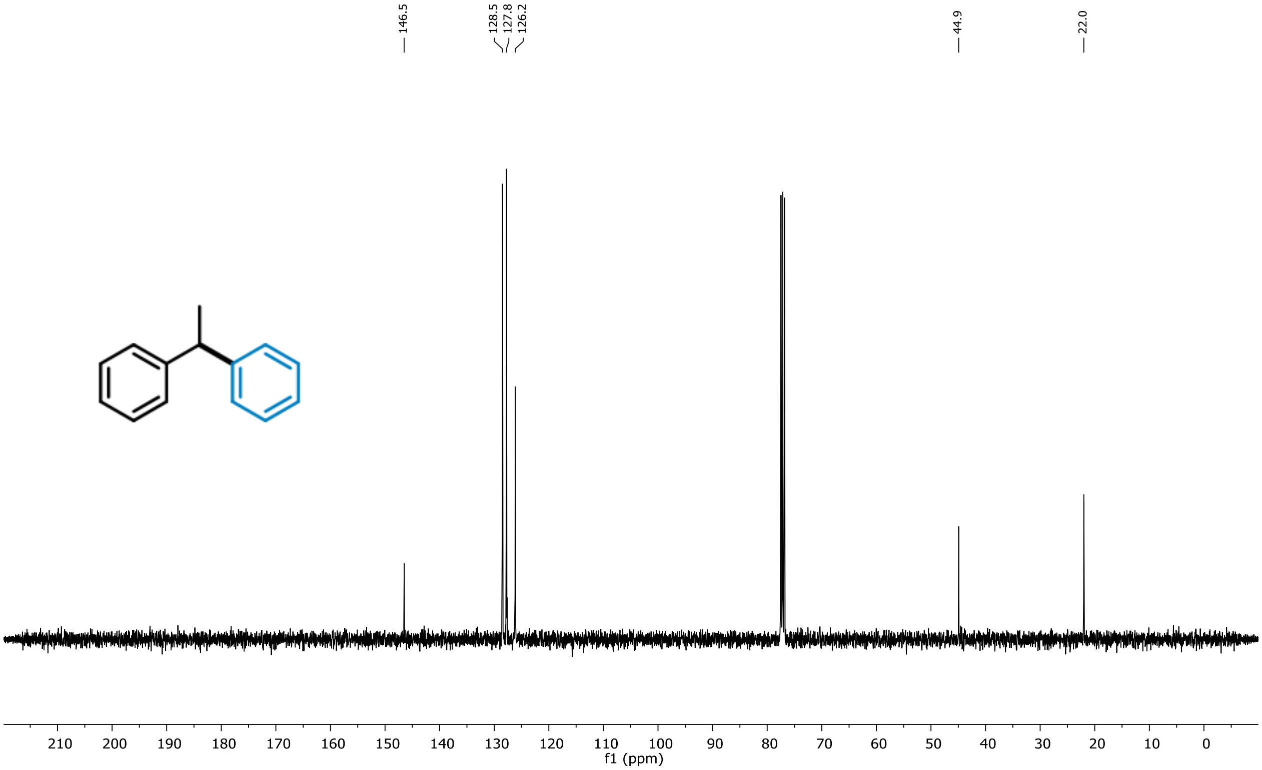

^{13}C{^{1}H} NMR (101 MHz, $CDCl_3$) ethane-1,1-diyldibenzene (**1h**)

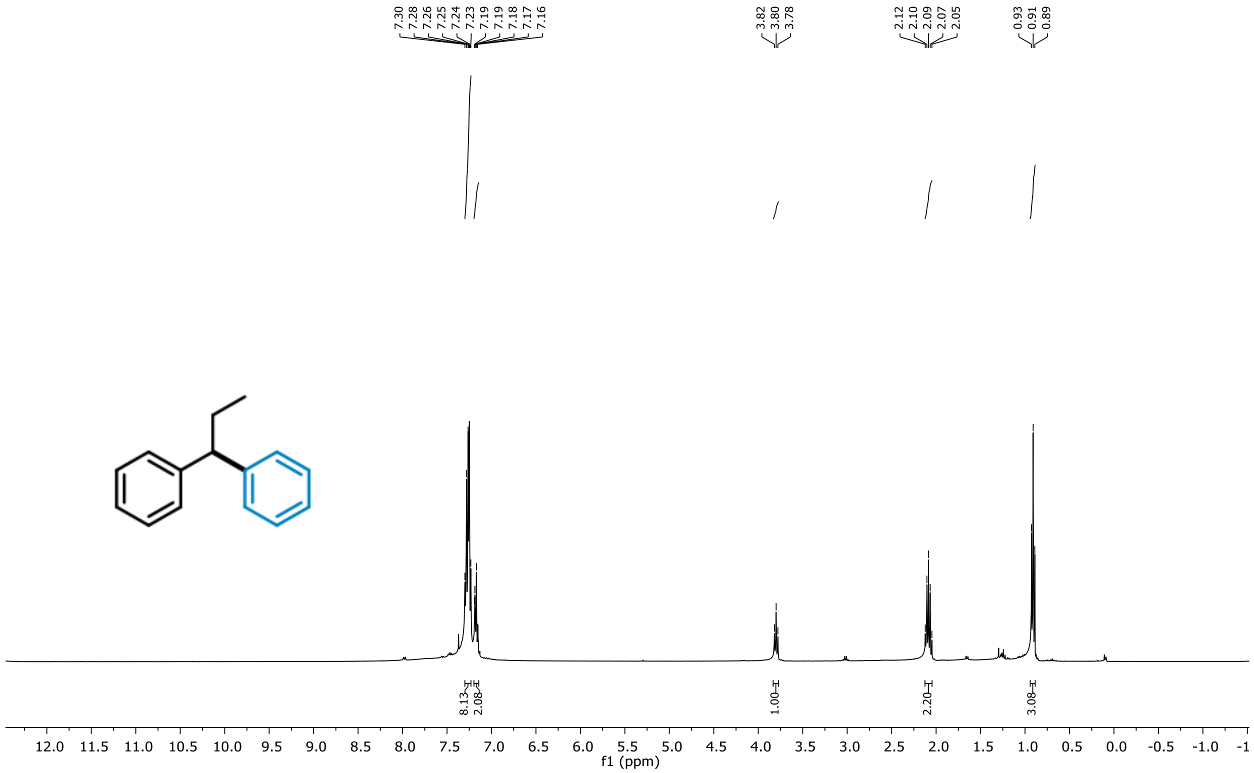

^{1}H NMR (400 MHz, $CDCl_3$) propane-1,1-diyldibenzene (**1i**)

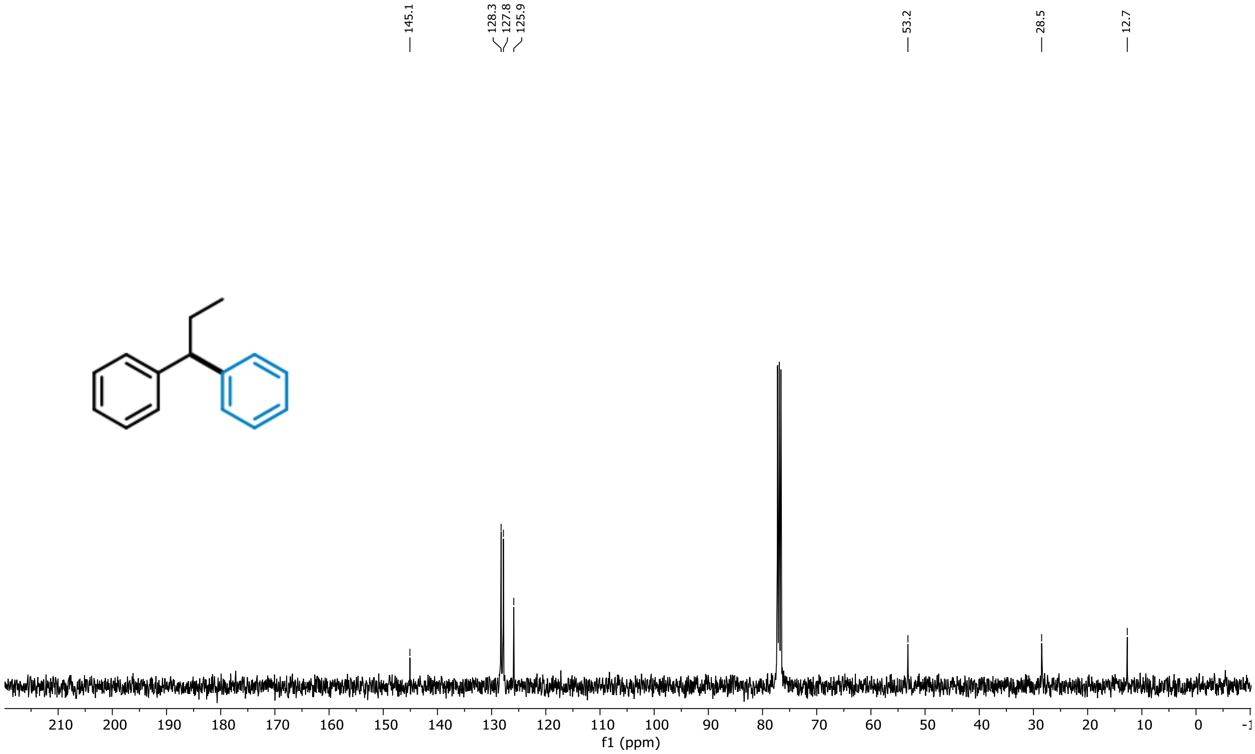

^{13}C{^{1}H} NMR (101 MHz, $CDCl_3$) propane-1,1-diyldibenzene (**1i**)

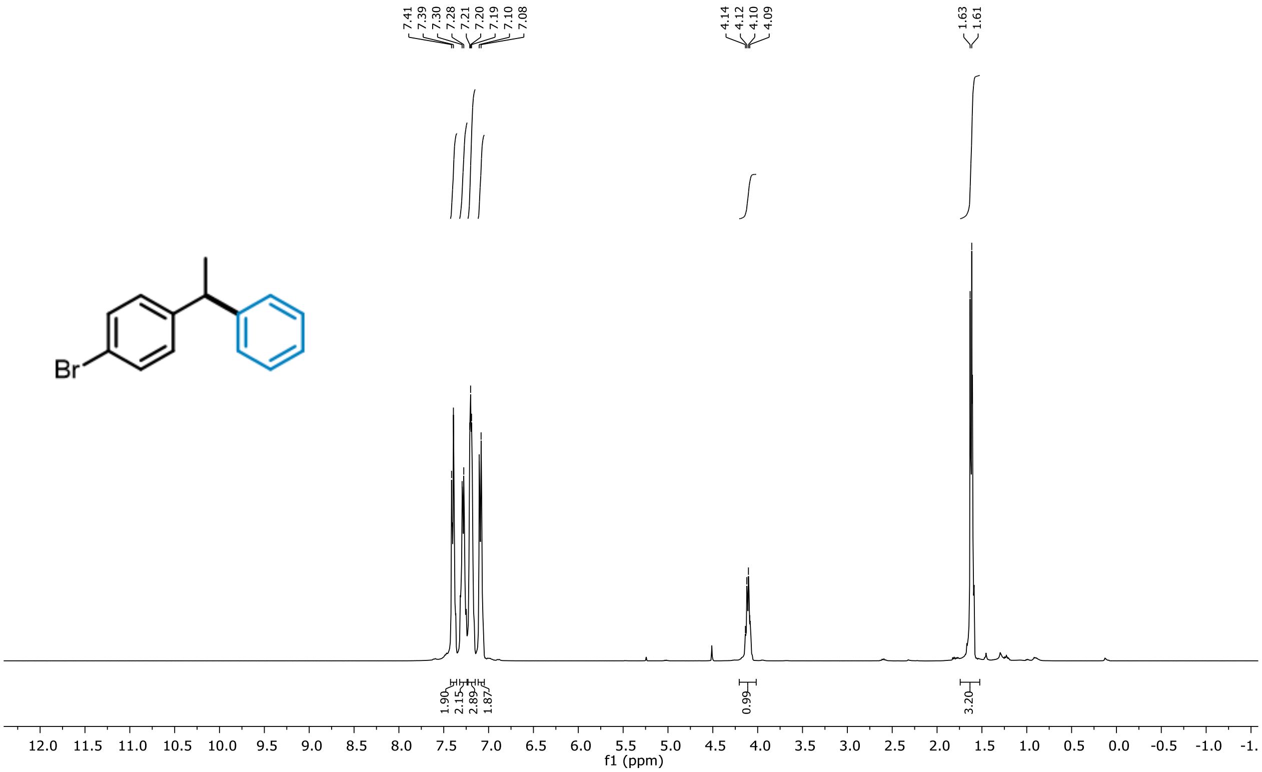

^{1}H NMR (400 MHz, $CDCl_3$) 1-bromo-4-(1-phenylethyl)benzene (**1j**)

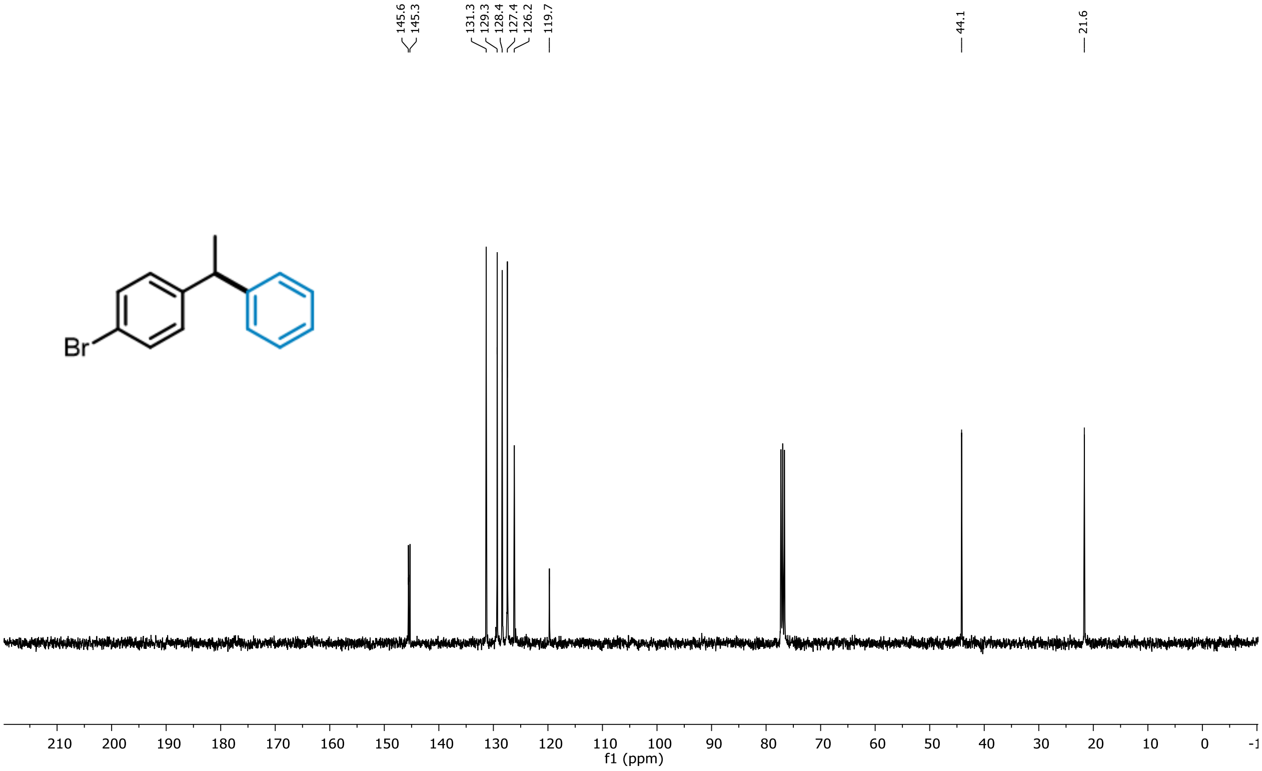

^{13}C{^{1}H} NMR (101 MHz, $CDCl_3$) 1-bromo-4-(1-phenylethyl)benzene (**1j**)

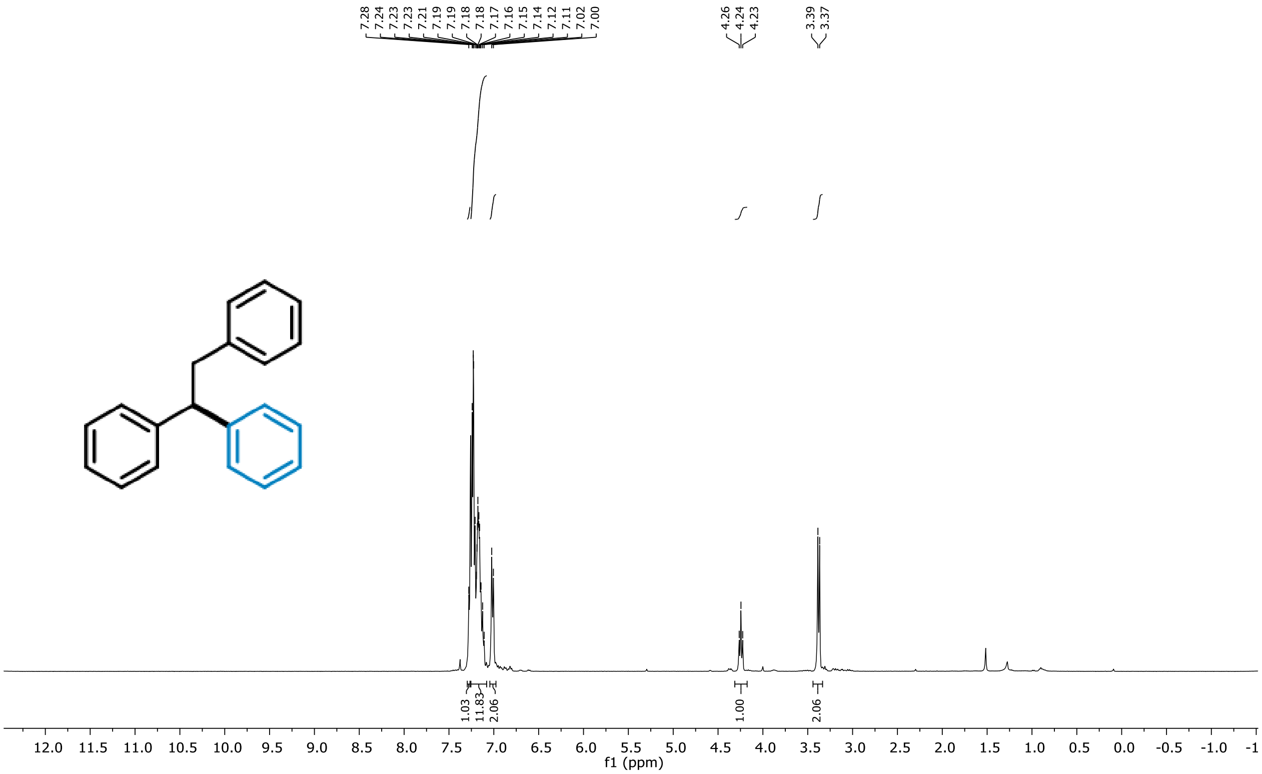

^{1}H NMR (400 MHz, $CDCl_3$) ethane-1,1,2-triyltribenzene (**1k**)

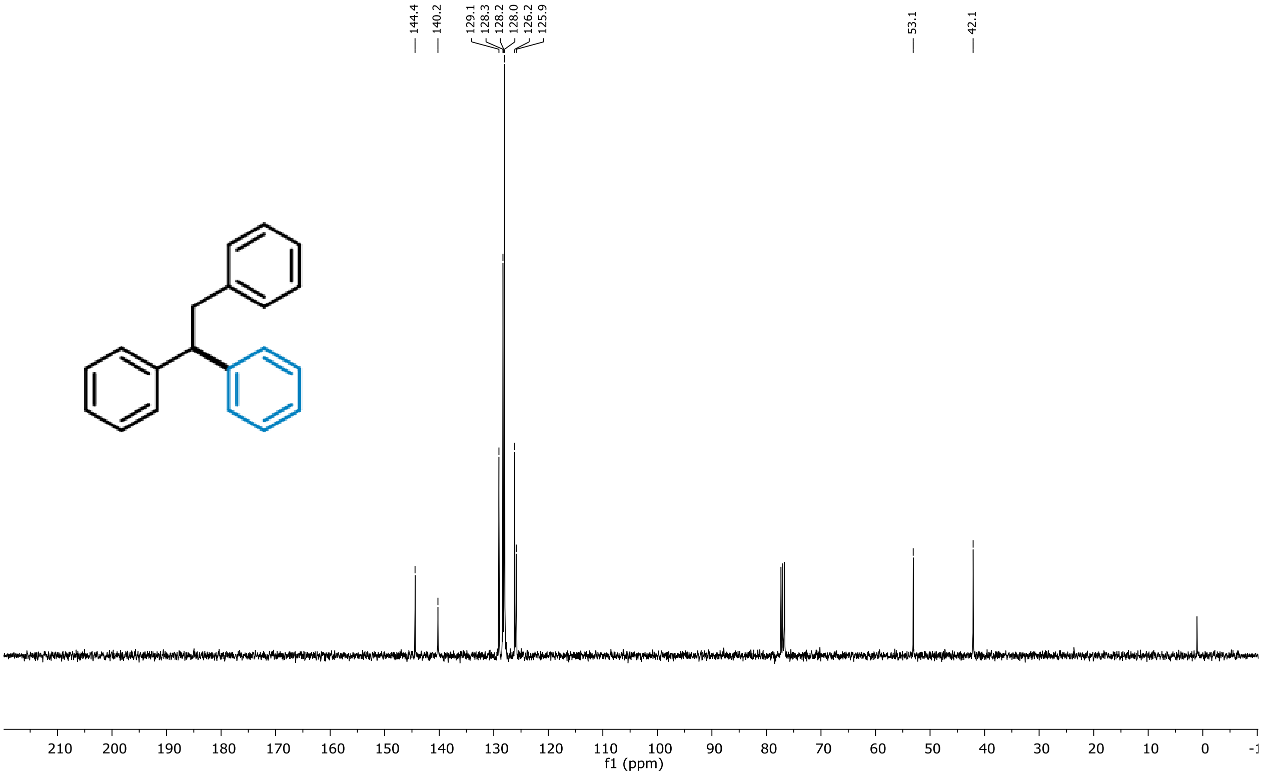

^{13}C{^{1}H} NMR (101 MHz, $CDCl_3$) ethane-1,1,2-triyltribenzene (**1k**)

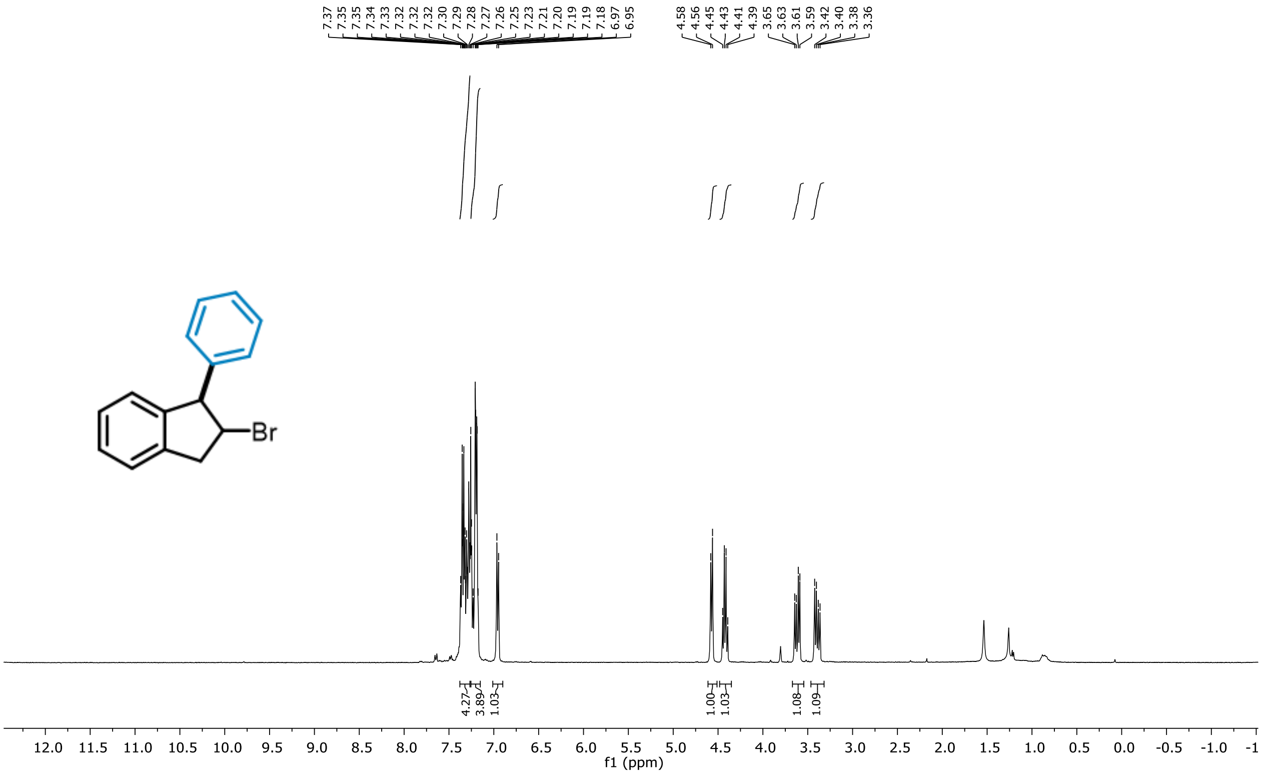

^{1}H NMR (400 MHz, $CDCl_3$) 2-bromo-1-phenyl-2,3-dihydro-1H-indene (**1l**)

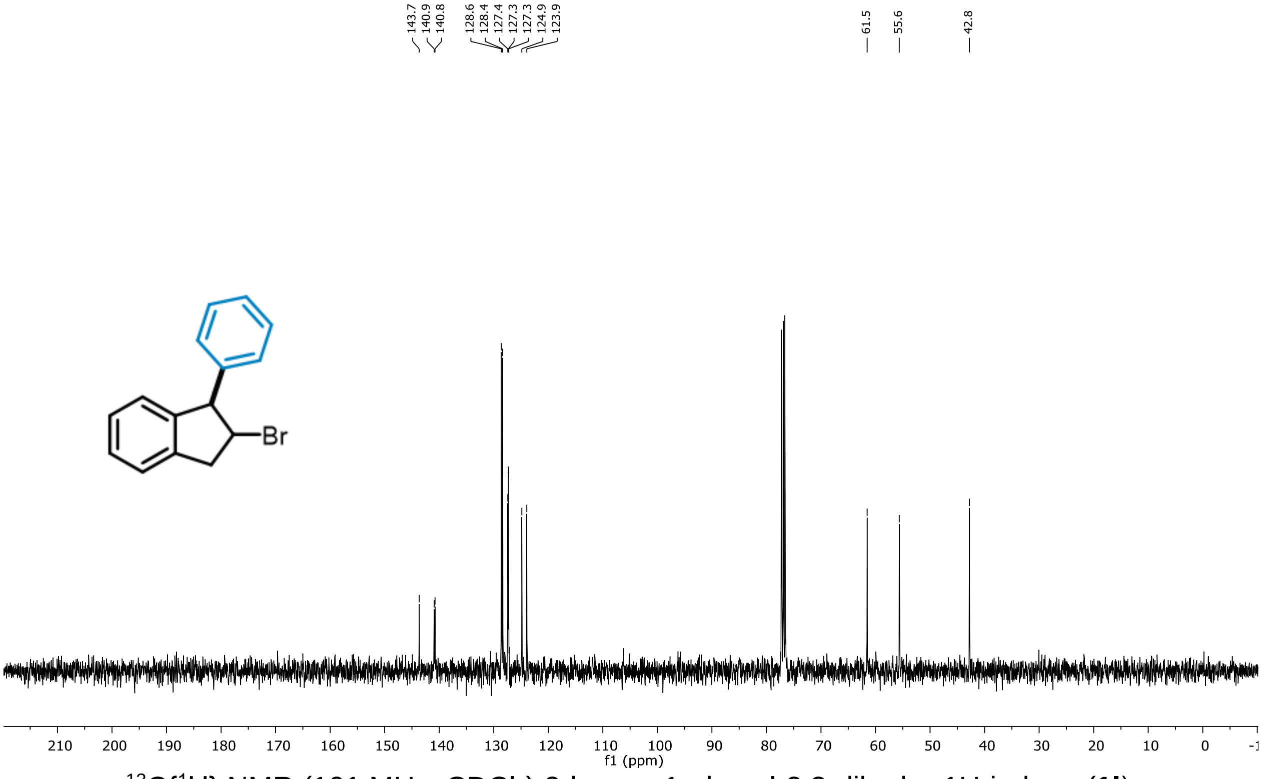

$^{13}C\{^{1}H\}$ NMR (101 MHz, $CDCl_3$) 2-bromo-1-phenyl-2,3-dihydro-1H-indene (**1l**)

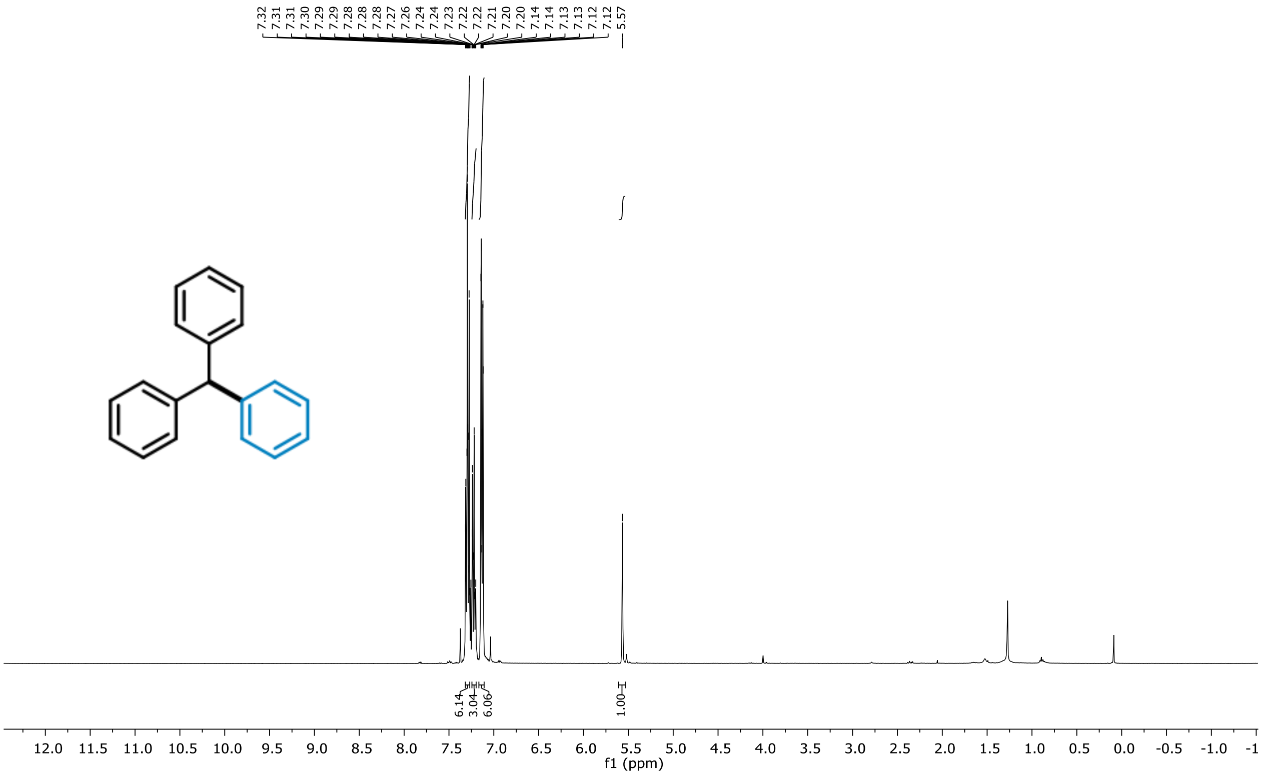

^{1}H NMR (400 MHz, $CDCl_3$) triphenylmethane (**1m**)

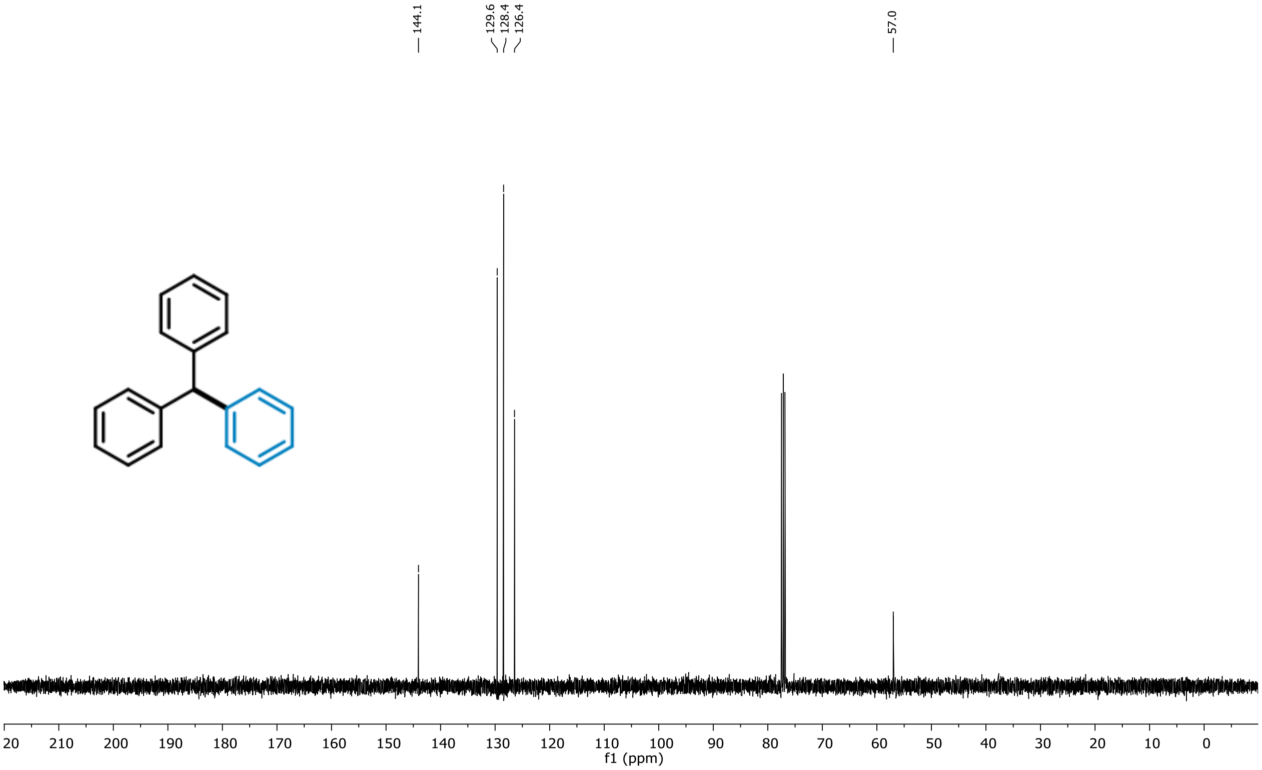

^{13}C{^{1}H} NMR (101 MHz, $CDCl_3$) triphenylmethane (**1m**)

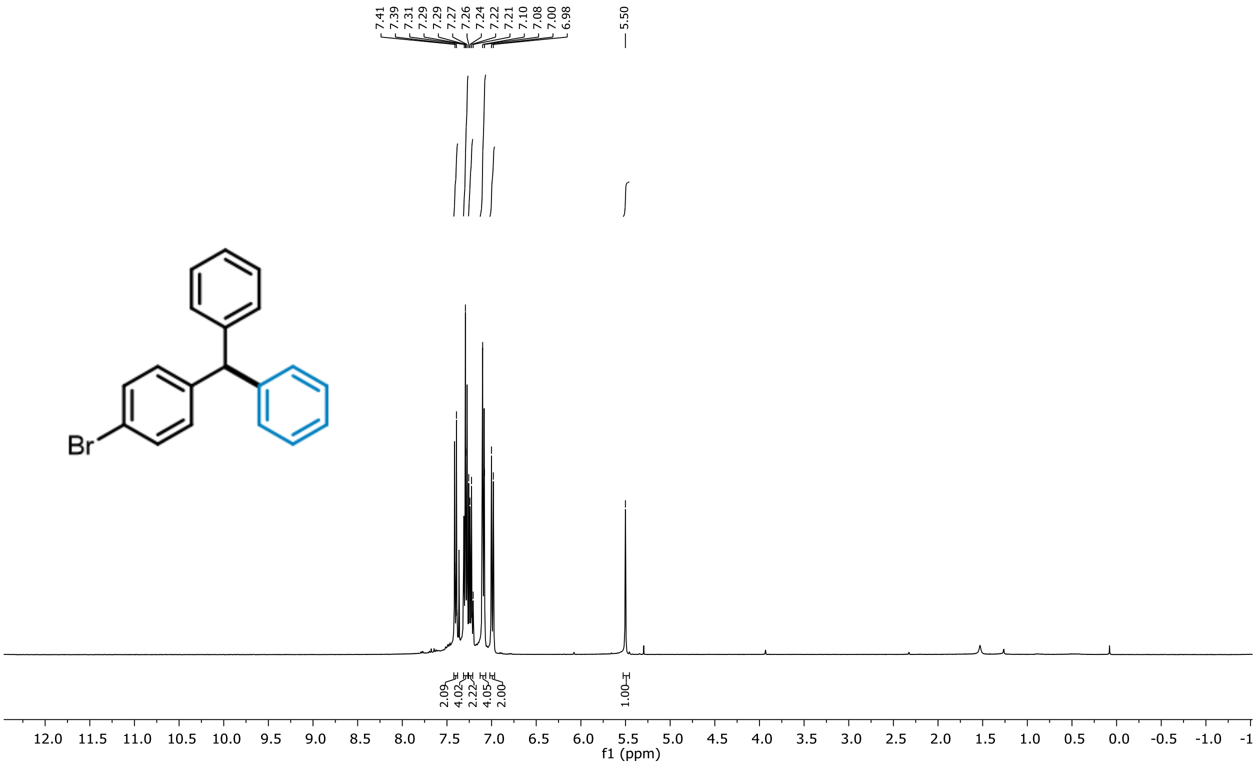

^{1}H NMR (400 MHz, $CDCl_3$) ((4-bromophenyl)methylene)dibenzene (**1n**)

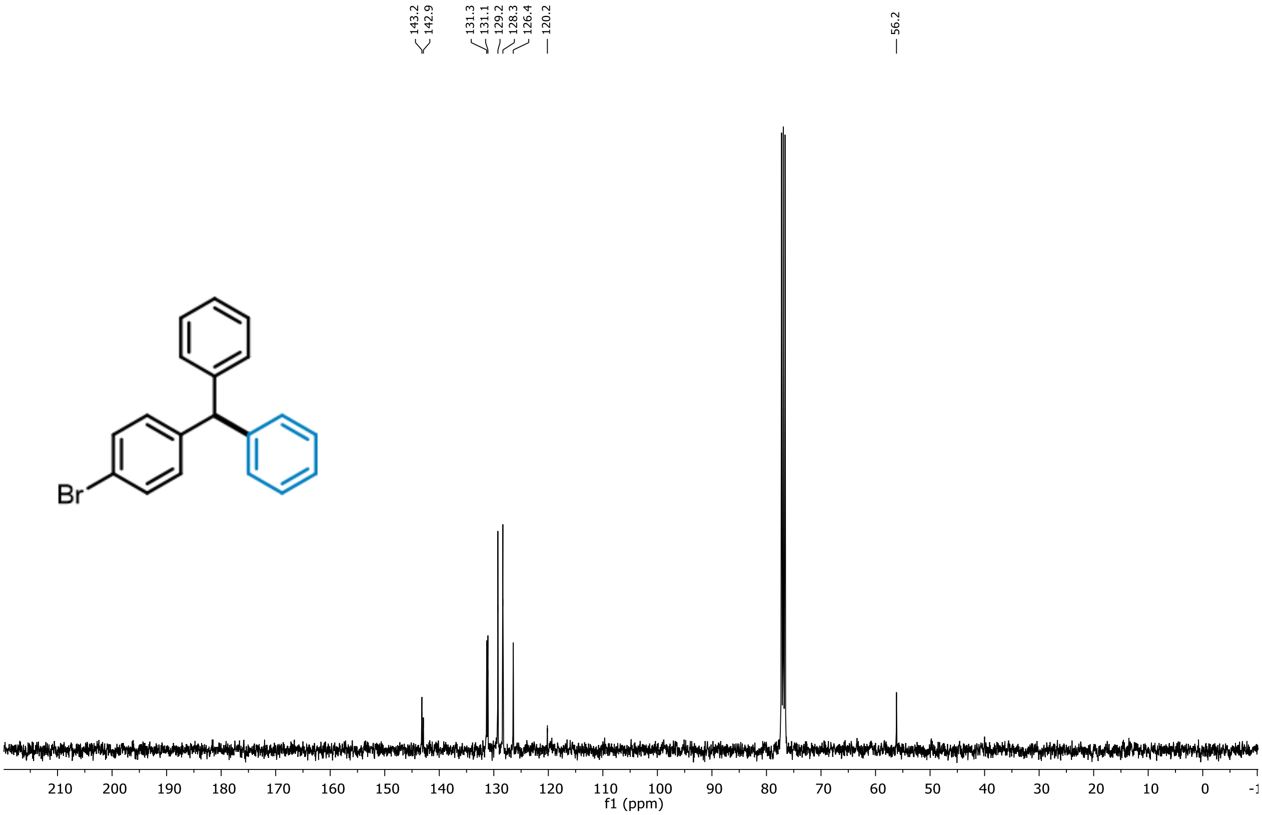

^{13}C{^{1}H} NMR (101 MHz, $CDCl_3$) ((4-bromophenyl)methylene)dibenzene (**1n**)

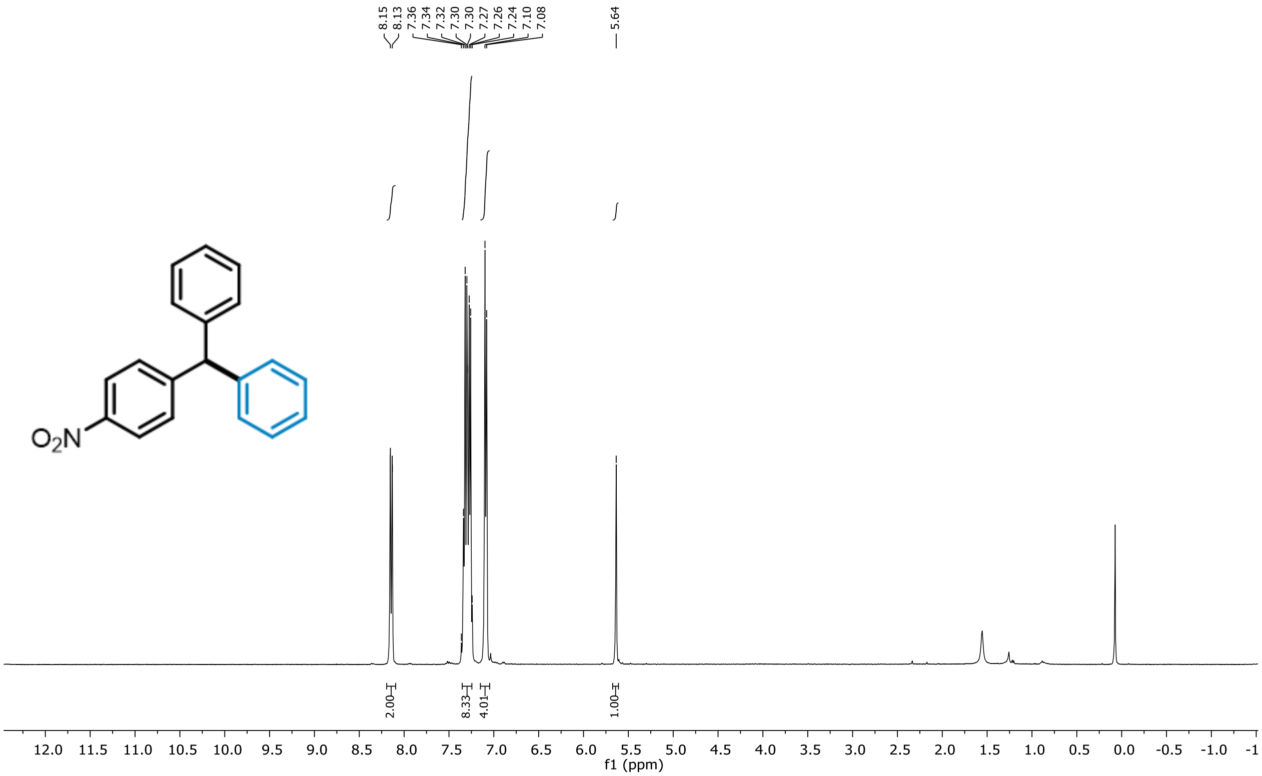

^{1}H NMR (400 MHz, $CDCl_3$) ((4-nitrophenyl)methylene)dibenzene (**1o**)

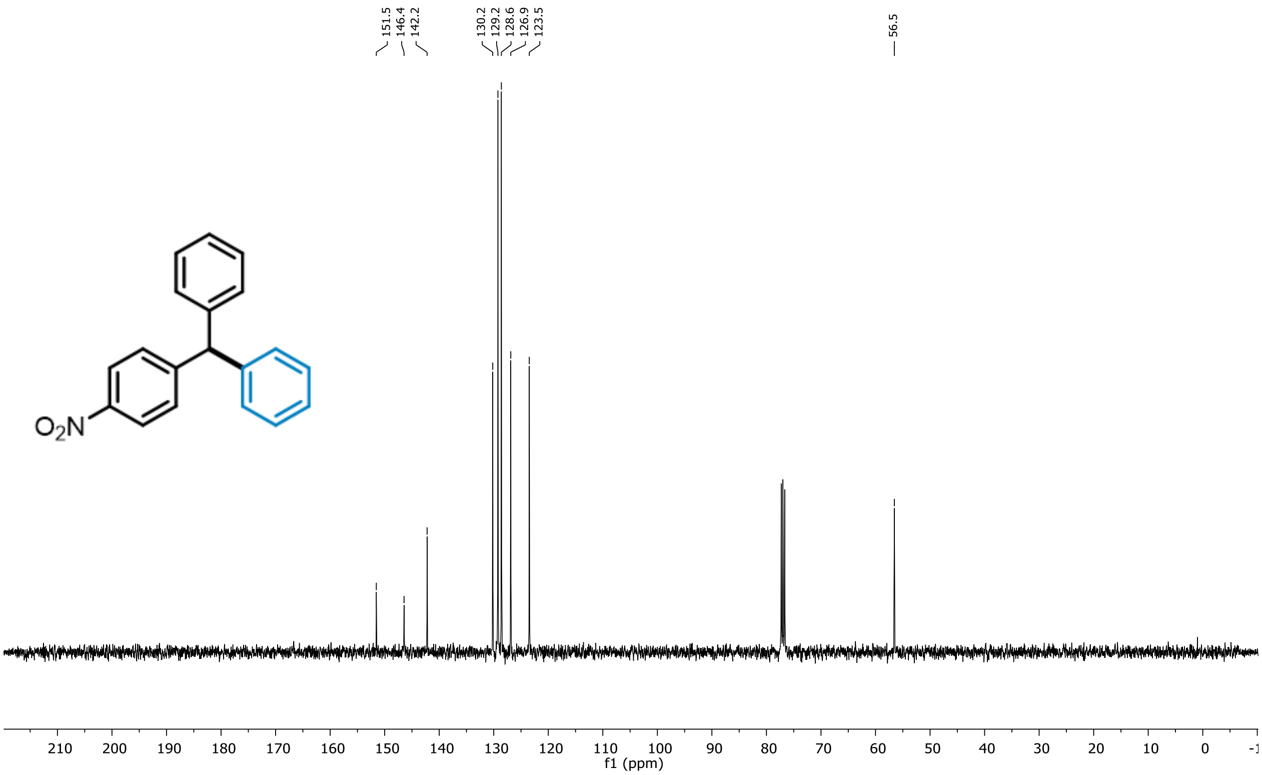

$^{13}C\{^{1}H\}$ NMR (101 MHz, $CDCl_3$) ((4-nitrophenyl)methylene)dibenzene (**1o**)

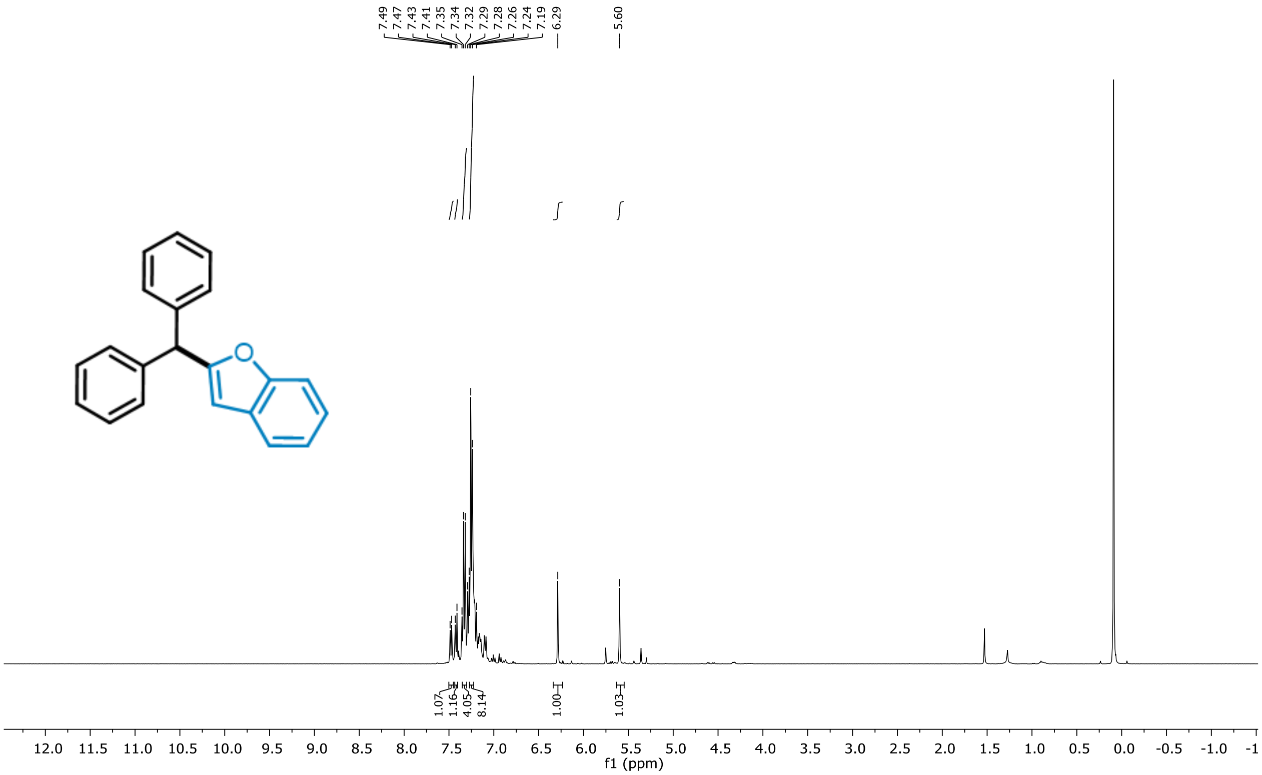

^{1}H NMR (400 MHz, $CDCl_3$) 2-benzhydrylbenzofuran (**1p**)

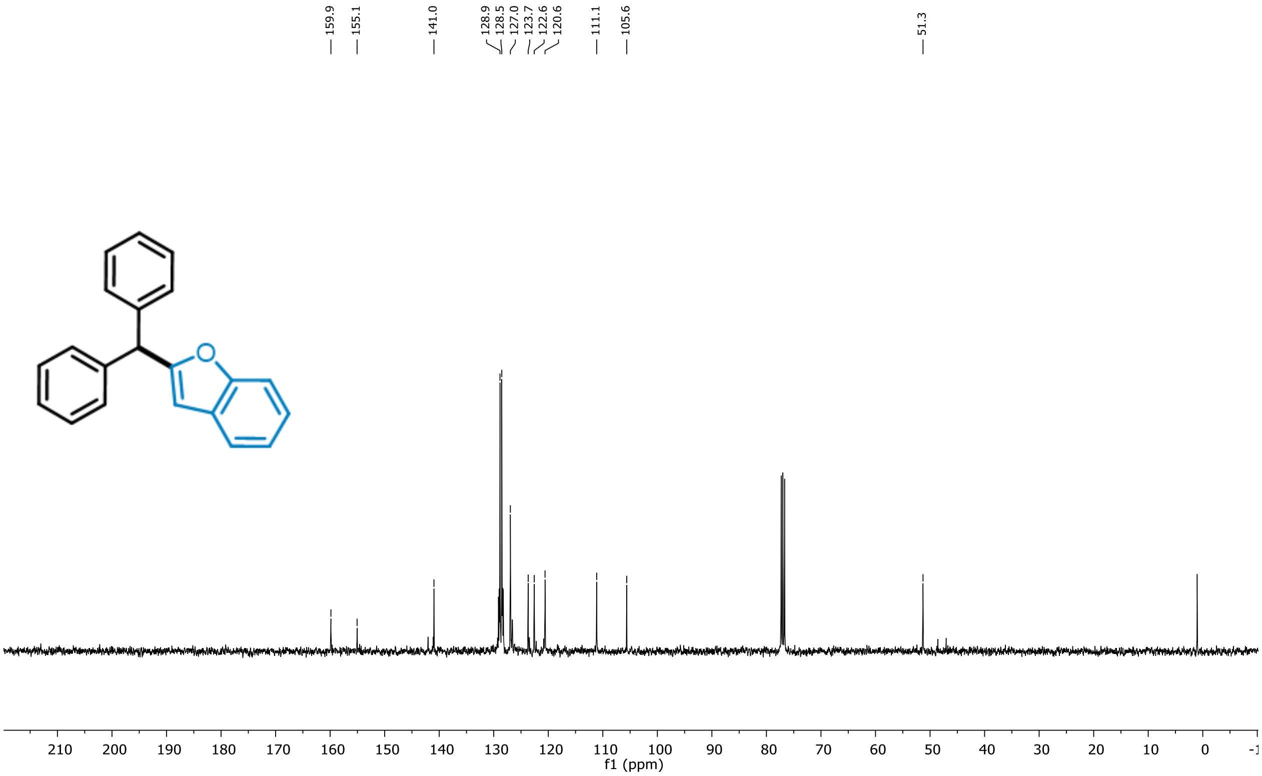

$^{13}C\{^{1}H\}$ NMR (101 MHz, $CDCl_3$) 2-benzhydrylbenzofuran (**1p**)

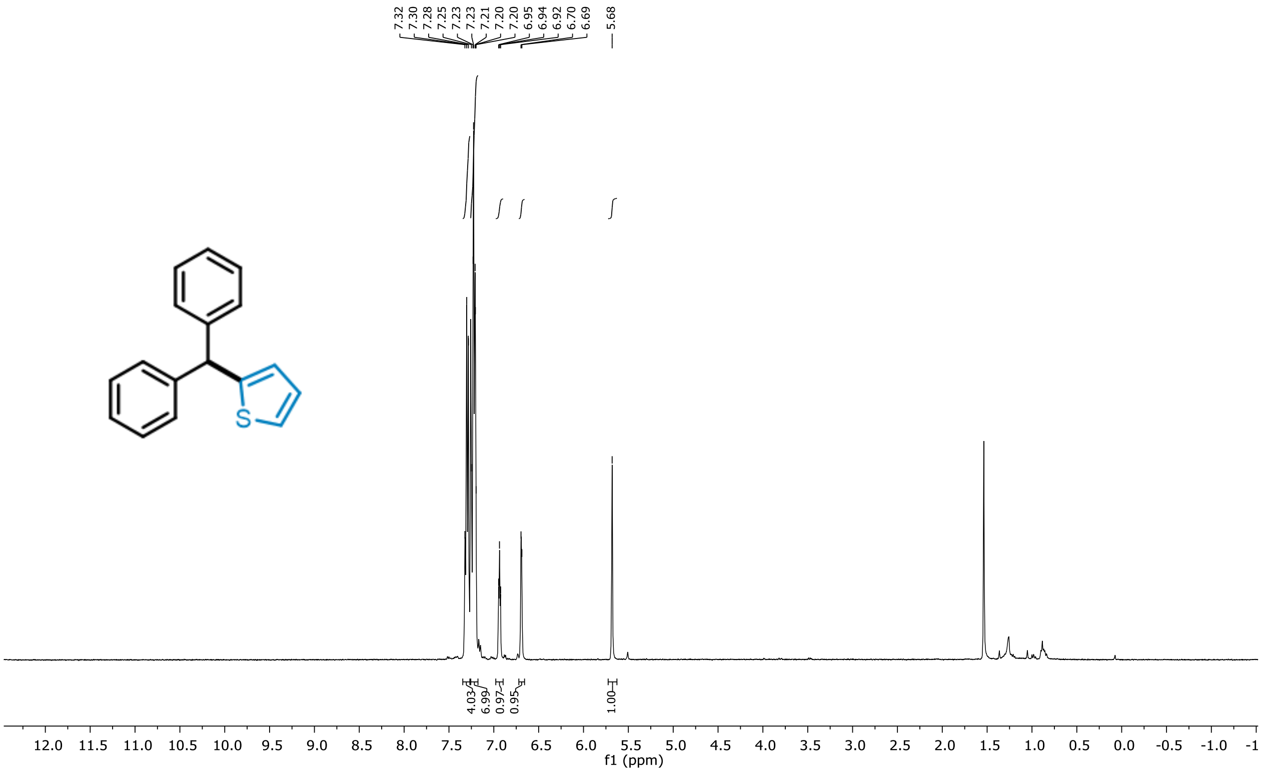

^{1}H NMR (400 MHz, $CDCl_3$) 3-benzhydrylthiophene (**1q**)

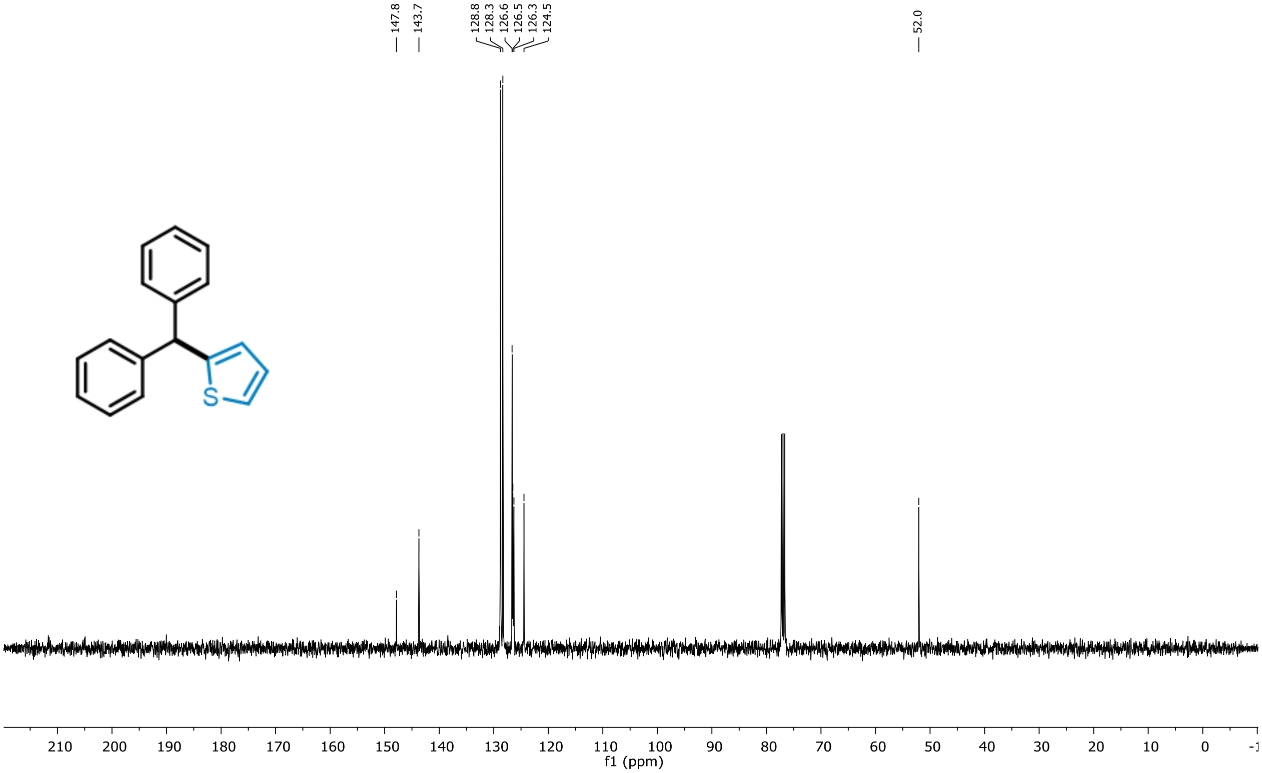

$^{13}C\{^{1}H\}$ NMR (101 MHz, $CDCl_3$) 3-benzhydrylthiophene (**1q**)

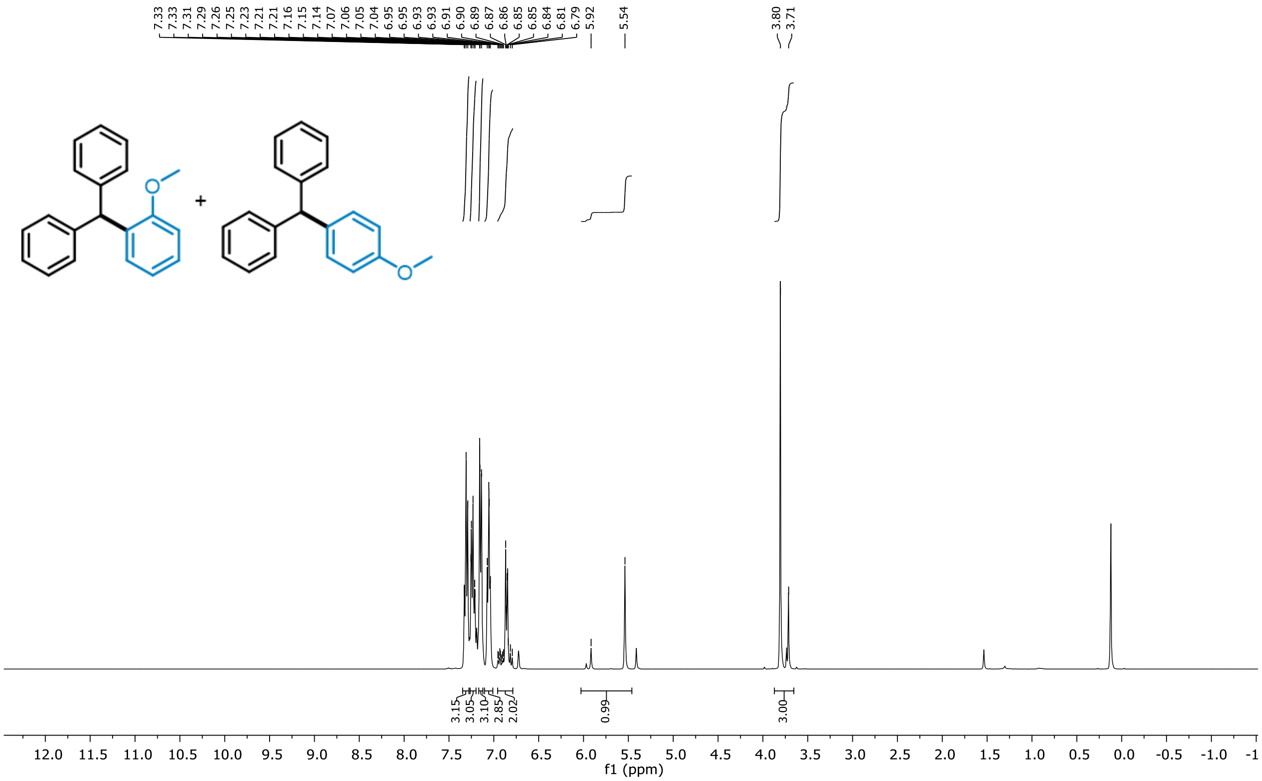

^{1}H NMR (400 MHz, $CDCl_3$) ((2-methoxyphenyl)methylene)dibenzene + ((4-methoxyphenyl)methylene)dibenzene (**1r**)

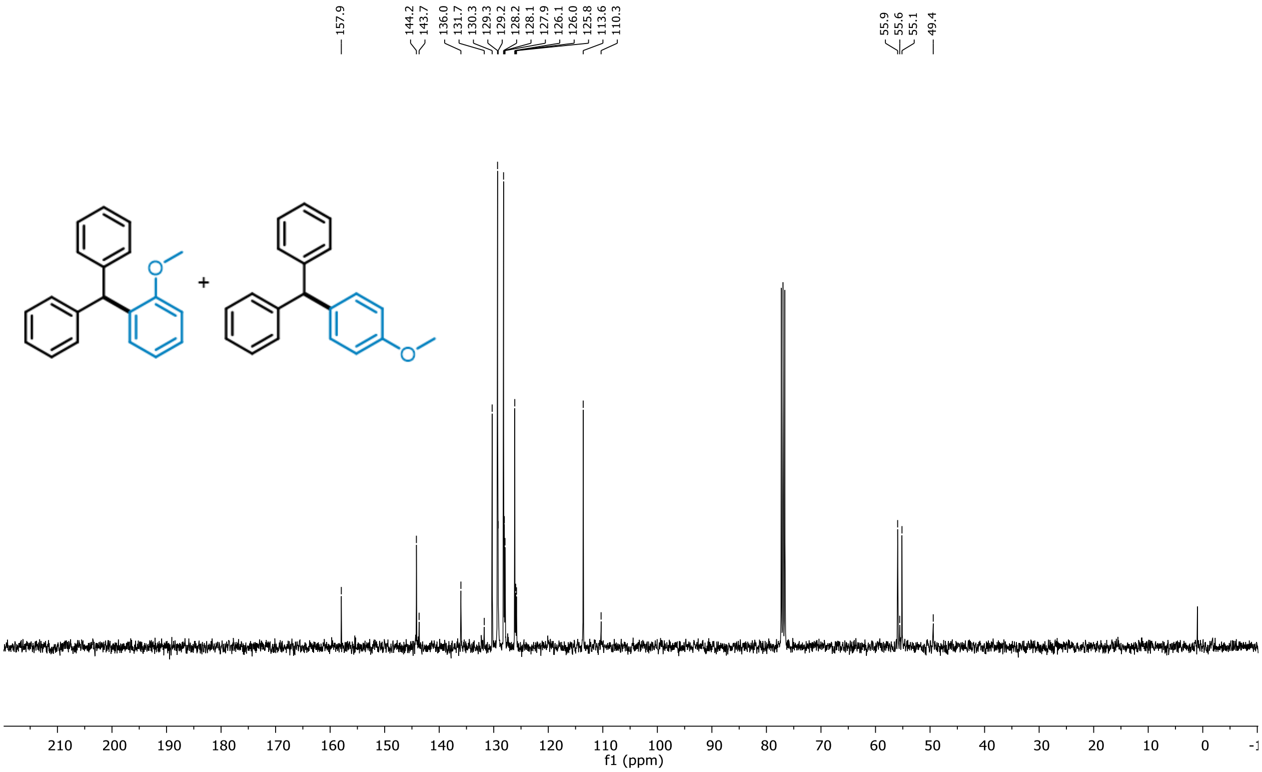

^{13}C{^{1}H} NMR (101 MHz, $CDCl_3$) ((2-methoxyphenyl)methylene)dibenzene + ((4-methoxyphenyl)methylene)dibenzene (**1r**)

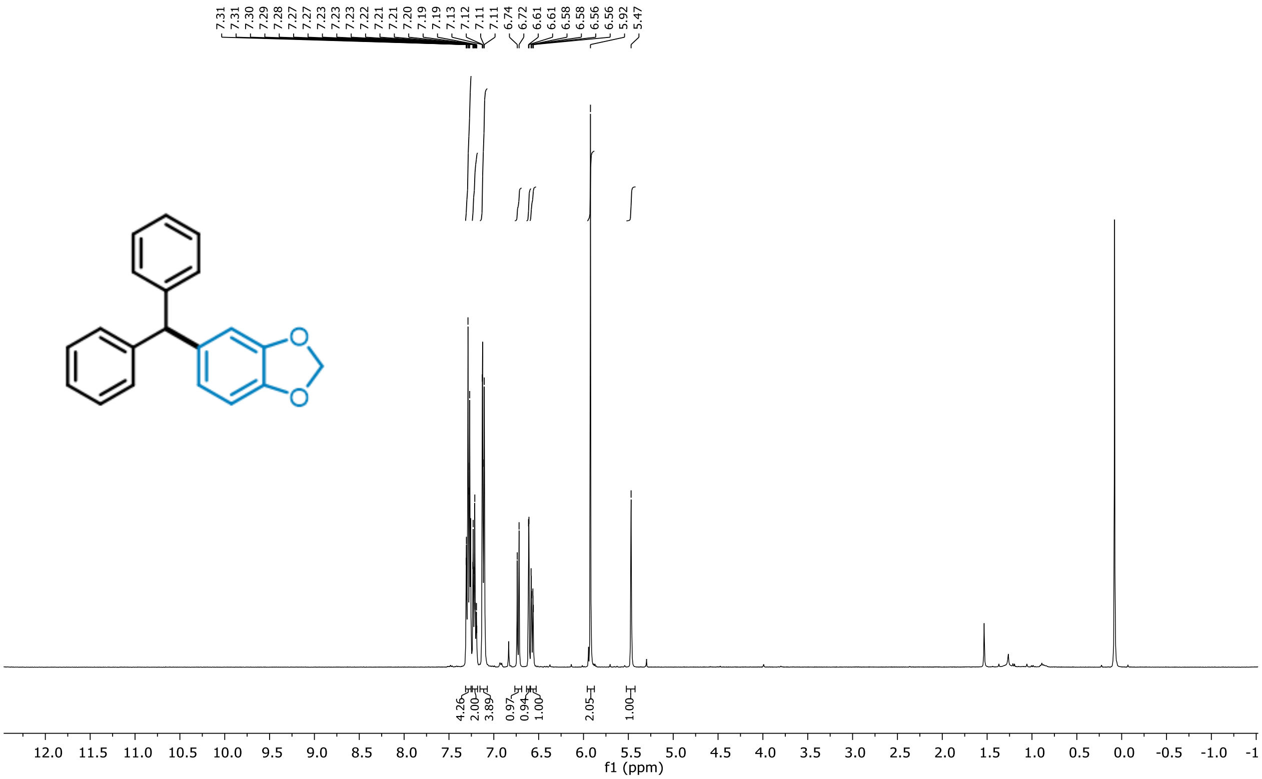

^{1}H NMR (400 MHz, $CDCl_3$) 5-benzhydrylbenzo[d][1,3]dioxole (**1s**)

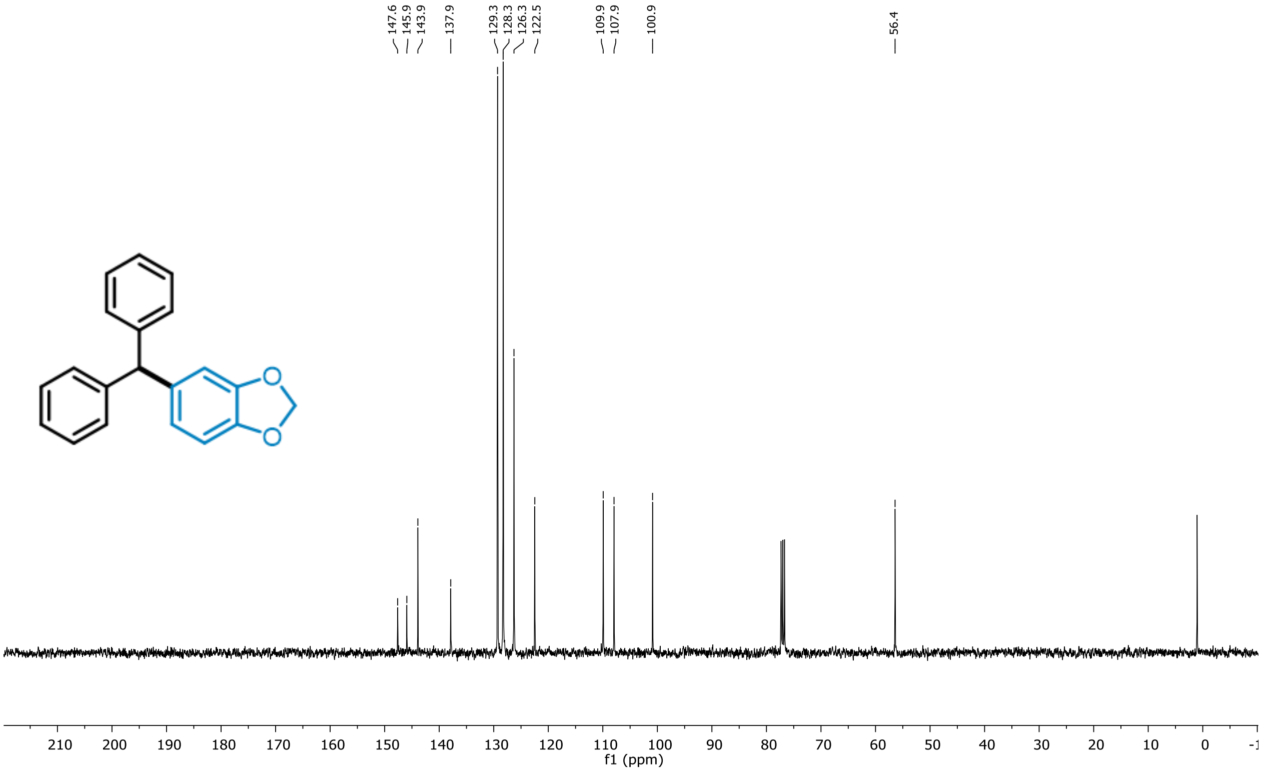

$^{13}C\{^1H\}$ NMR (101 MHz, $CDCl_3$) 5-benzhydrylbenzo[d][1,3]dioxole (**1s**)

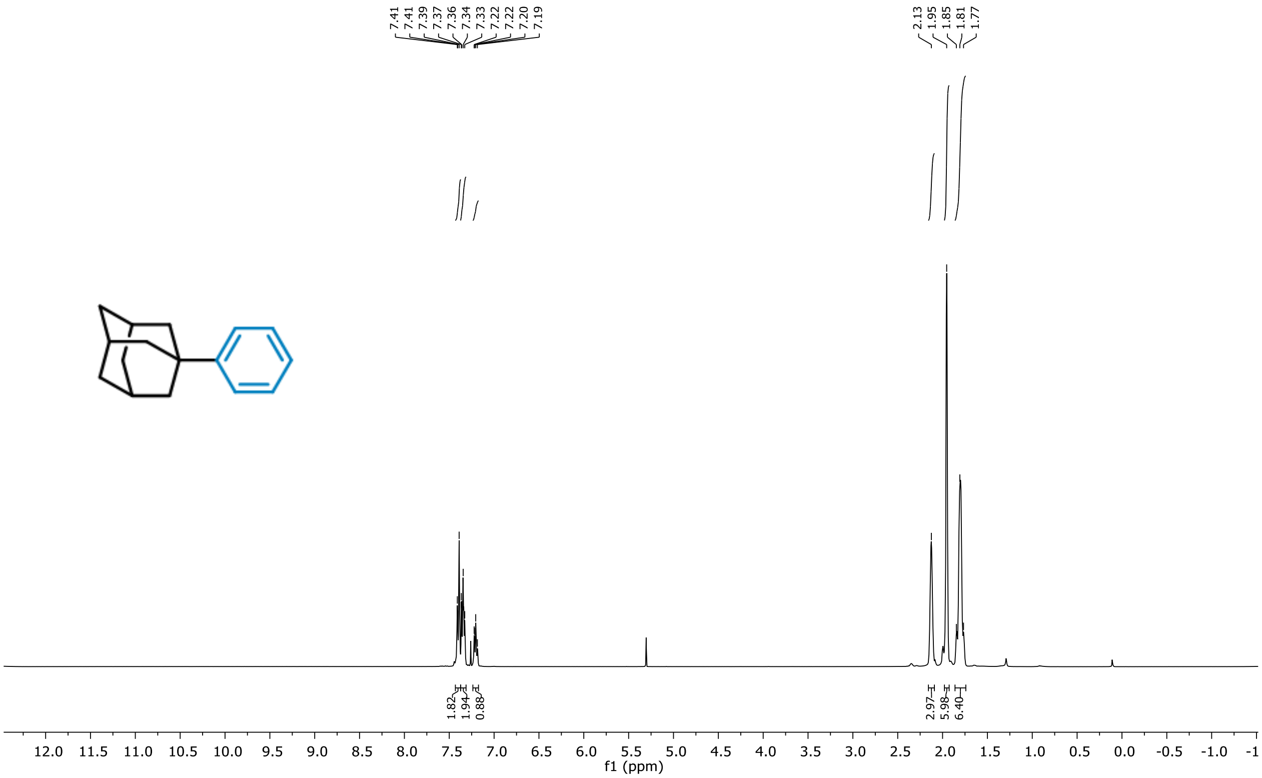

^{1}H NMR (400 MHz, $CDCl_3$) 1-phenyladamantane (**1t**)

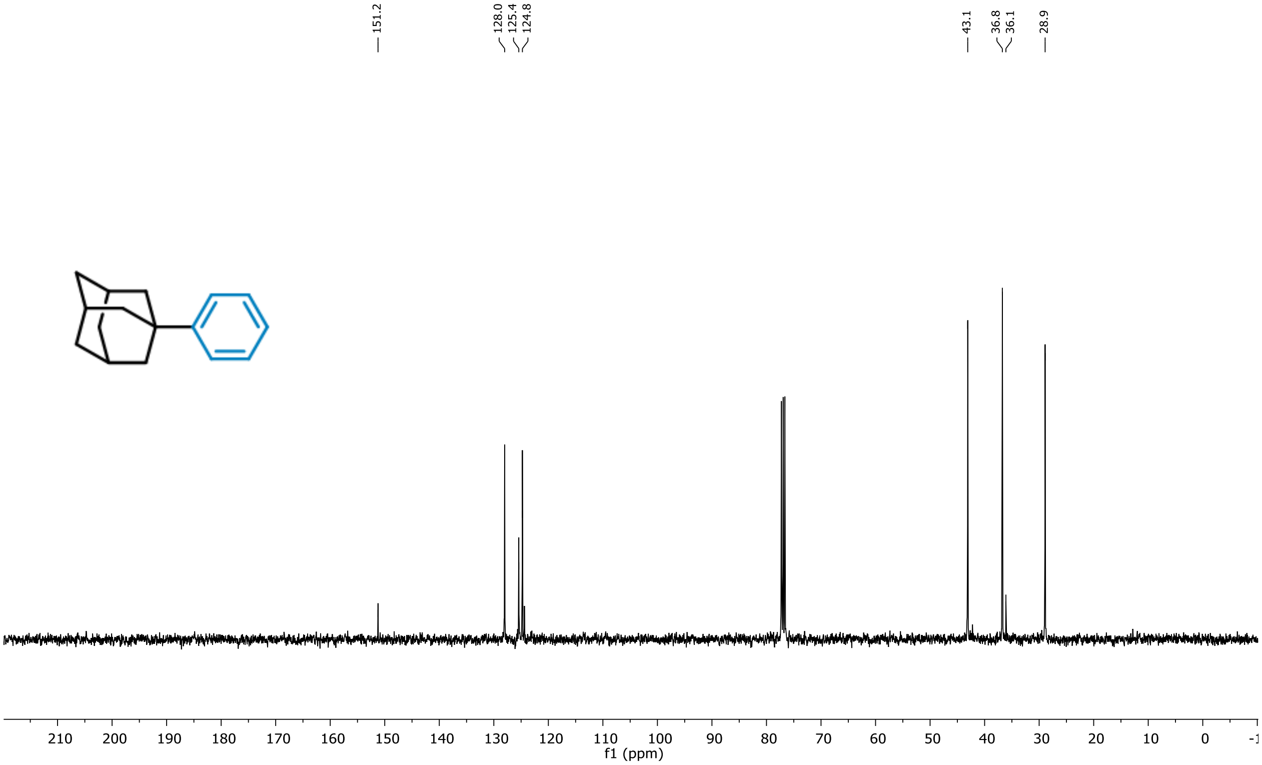

$^{13}C\{^{1}H\}$ NMR (101 MHz, $CDCl_3$) 1-phenyladamantane (**1t**)

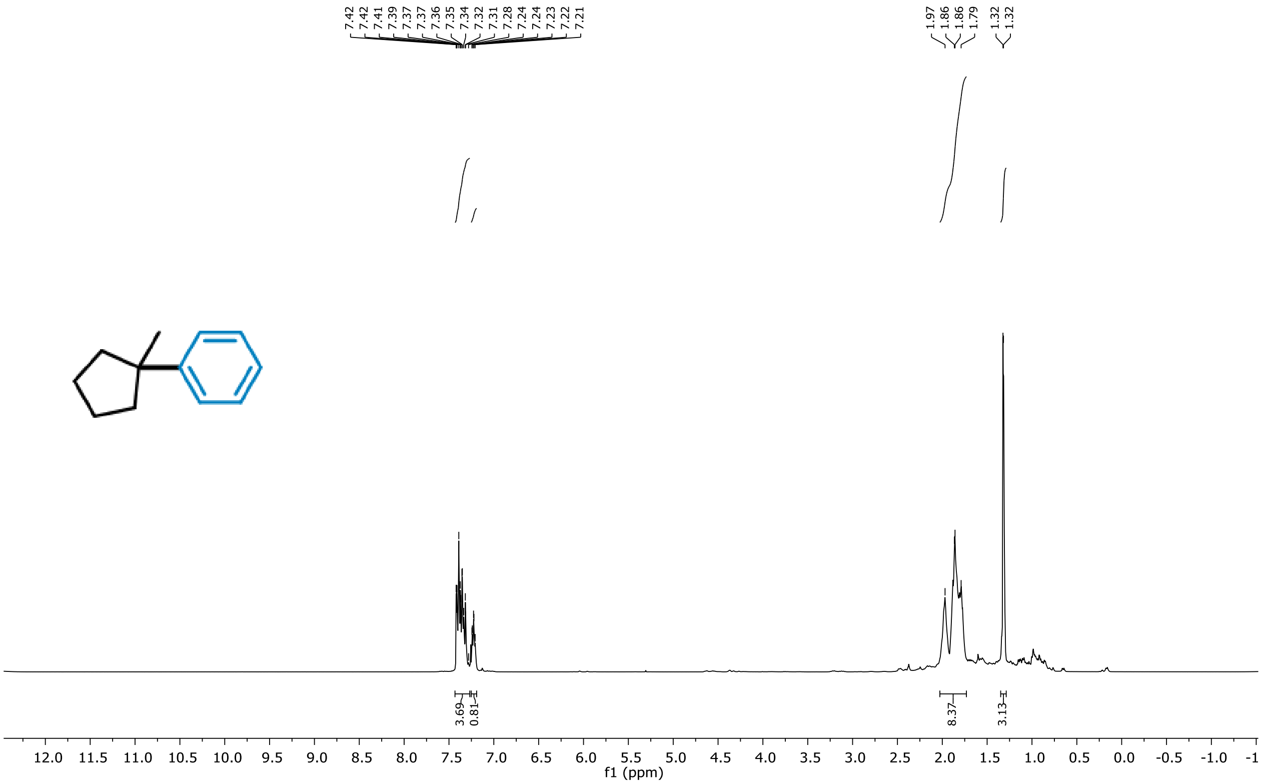

^{1}H NMR (400 MHz, $CDCl_3$) (1-methylcyclopentyl)benzene (**1u**)

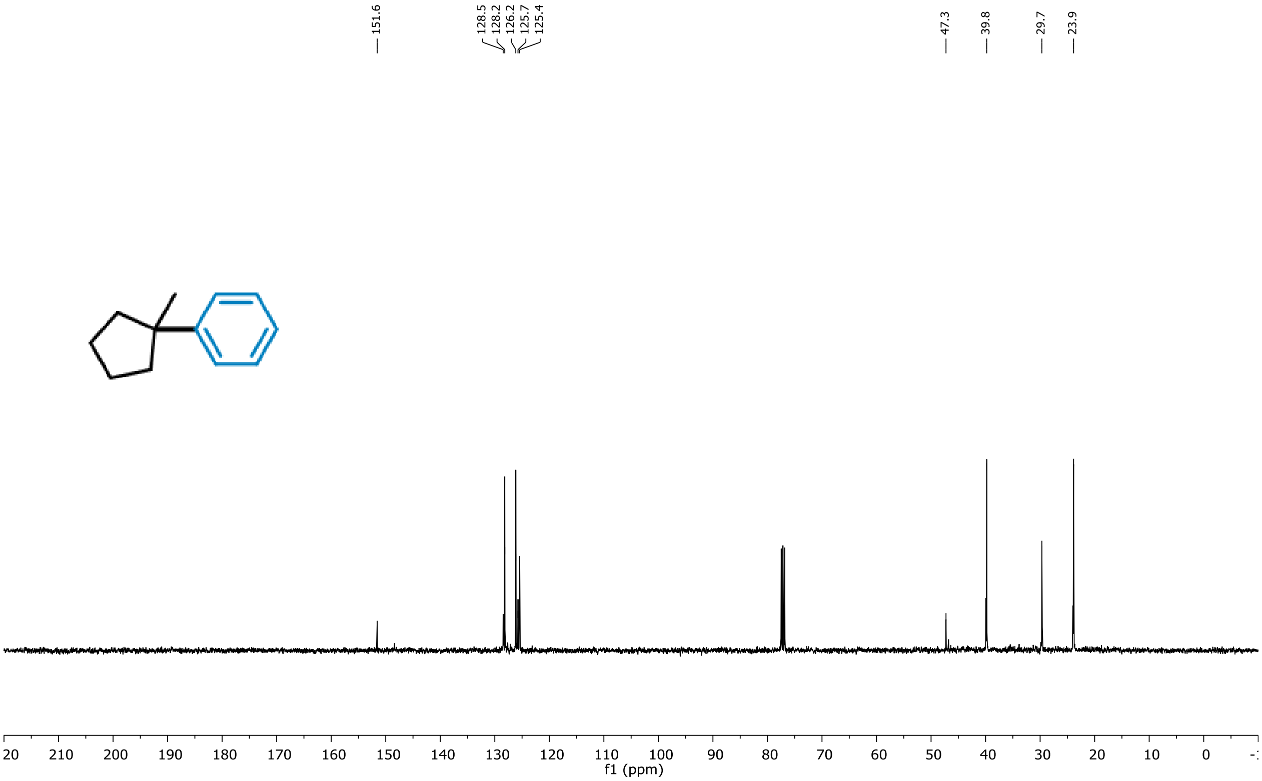

$^{13}C\{^1H\}$ NMR (101 MHz, $CDCl_3$) (1-methylcyclopentyl)benzene (**1u**)

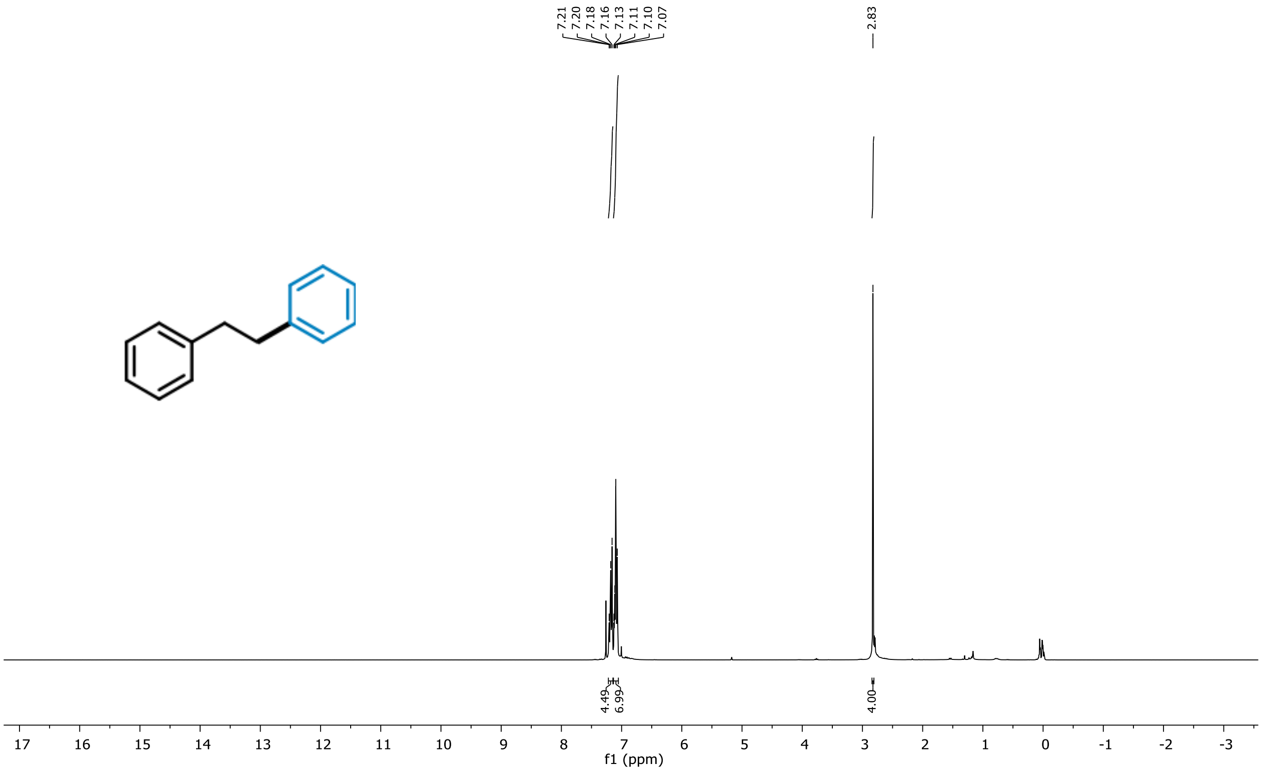

^{1}H NMR (400 MHz, $CDCl_3$) 1,2-diphenylethane (**1v**)

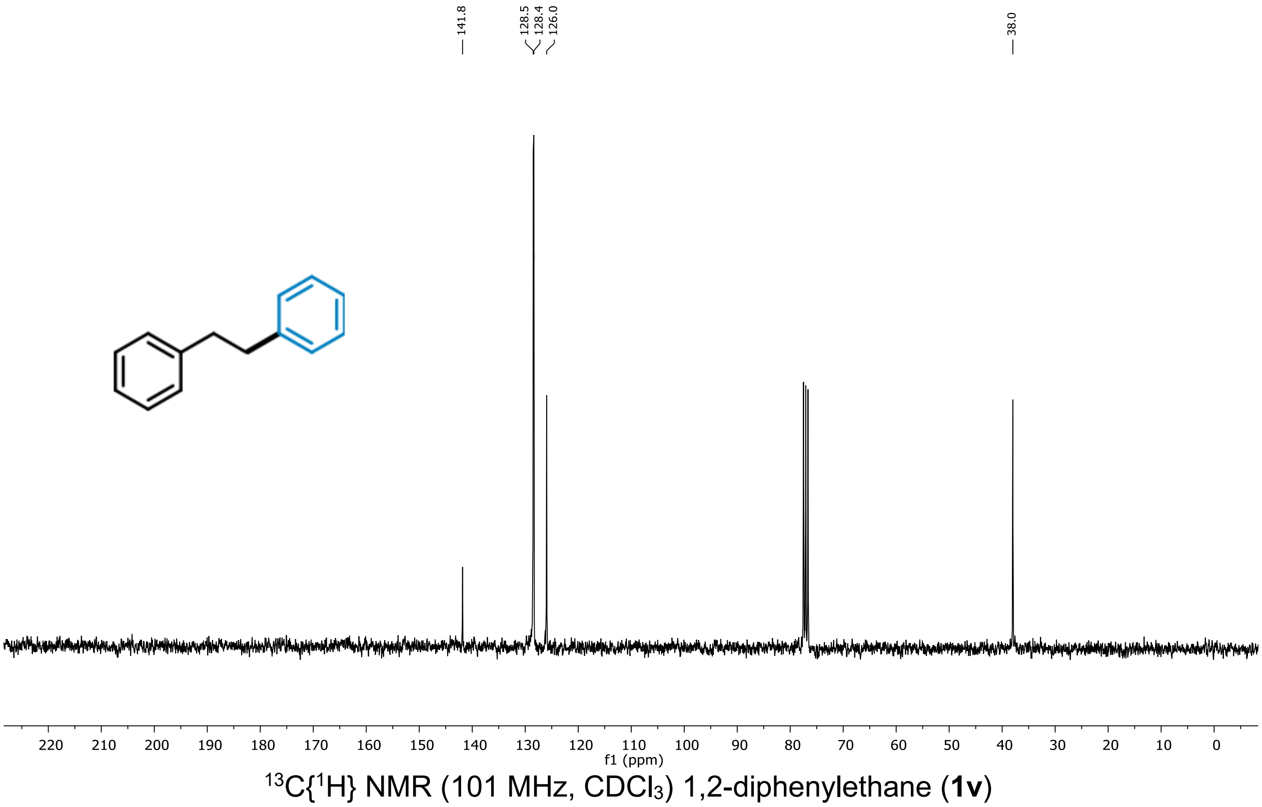

$^{13}C\{^{1}H\}$ NMR (101 MHz, $CDCl_3$) 1,2-diphenylethane (**1v**)

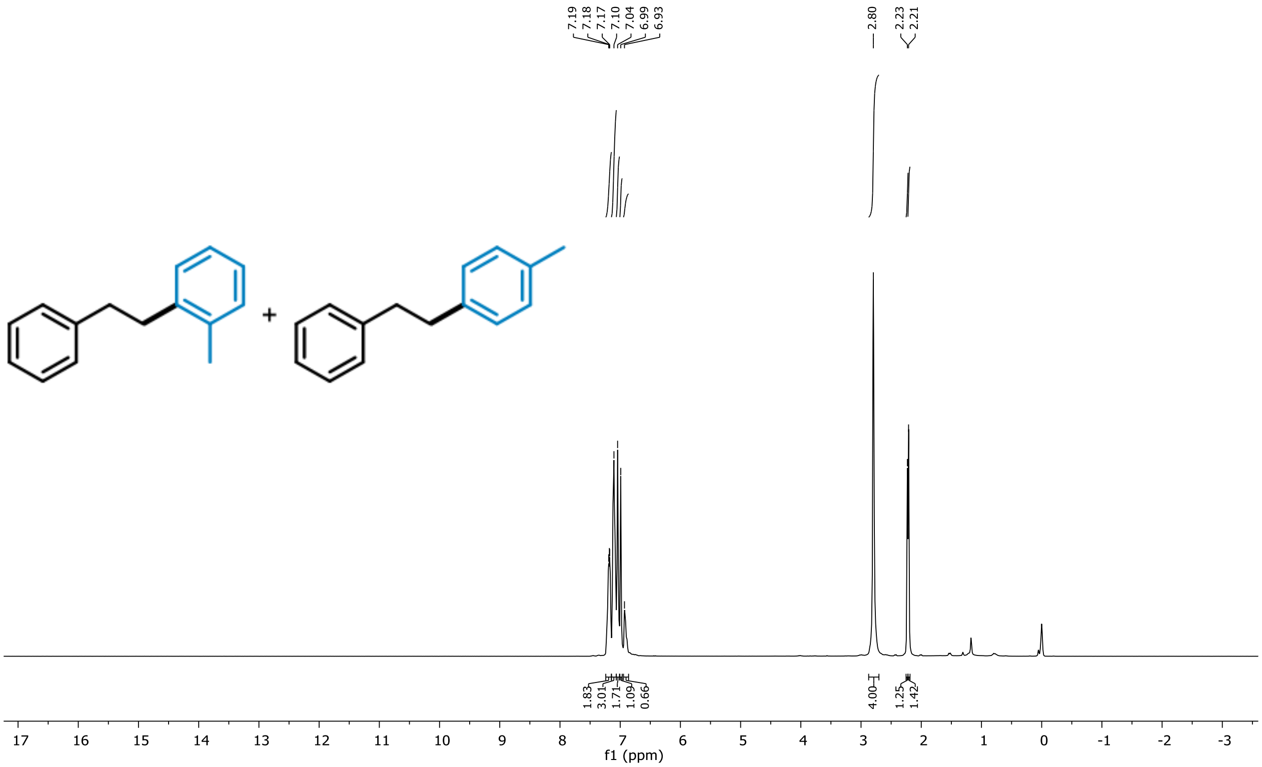

^{1}H NMR (400 MHz, $CDCl_3$) 1-methyl-2-phenethylbenzene + 1-methyl-4-phenethylbenzene (**1w**)

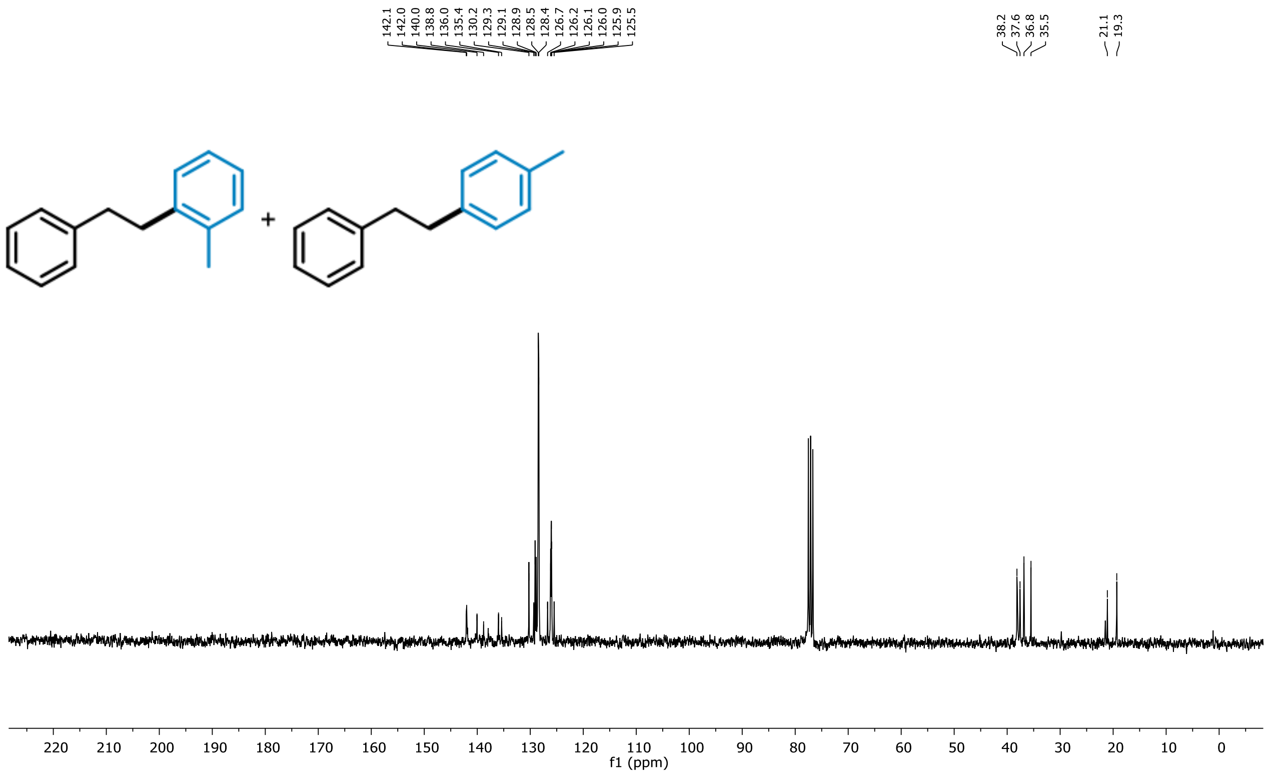

$^{13}C\{^{1}H\}$ NMR (101 MHz, $CDCl_3$) 1-methyl-2-phenethylbenzene + 1-methyl-4-phenethylbenzene (**1w**)

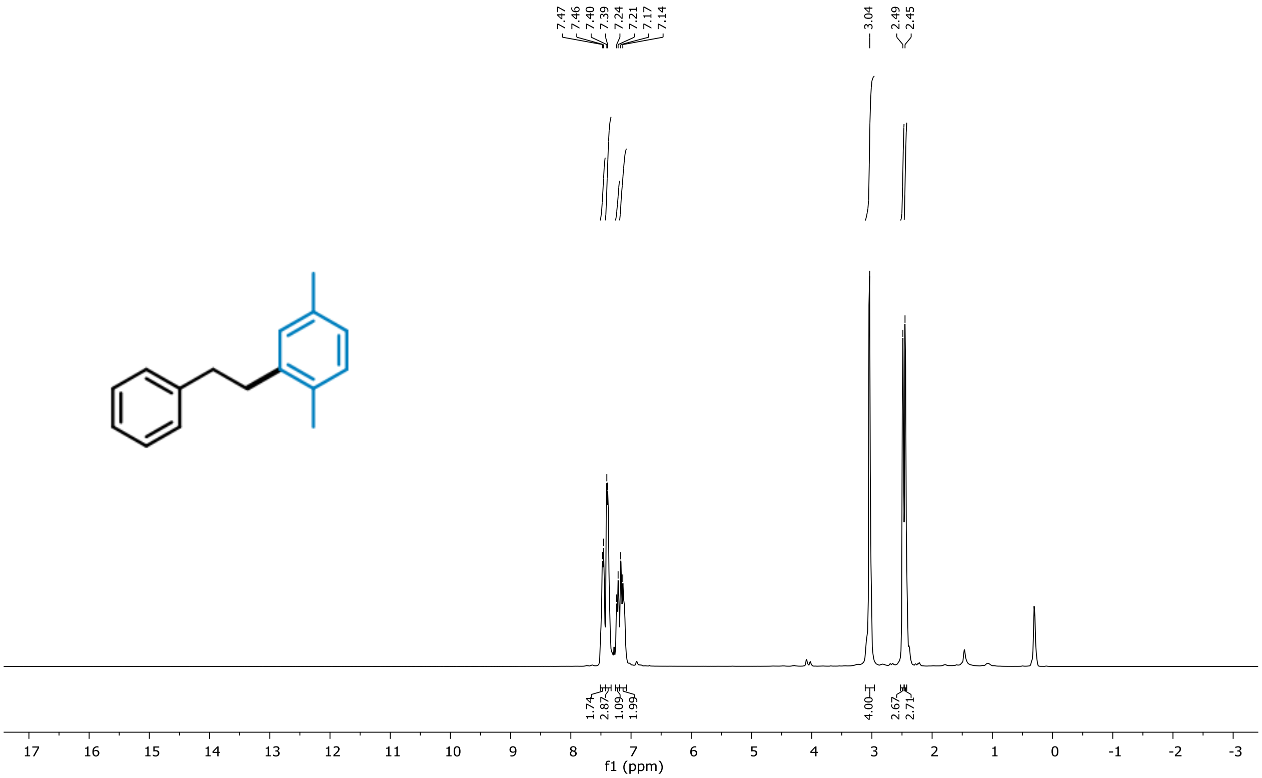

^{1}H NMR (400 MHz, $CDCl_3$) 1,4-dimethyl-2-phenethylbenzene (**1x**)

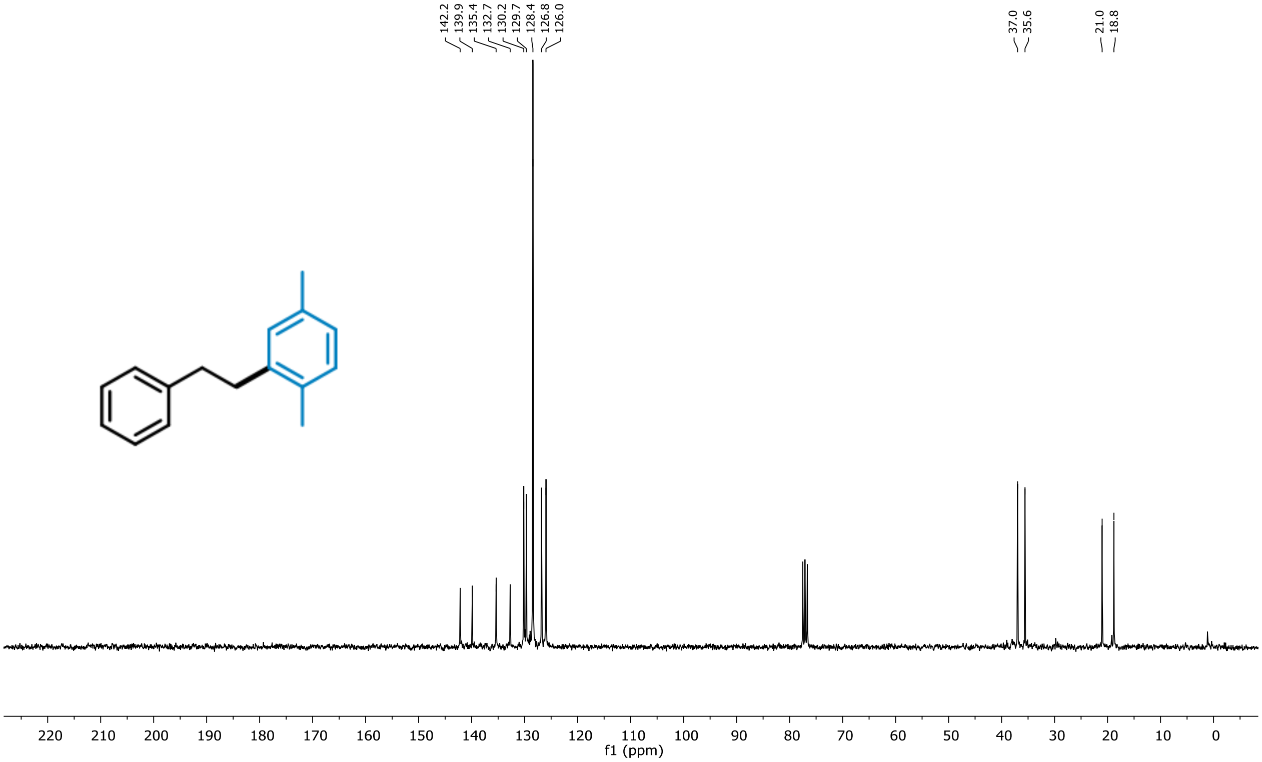

$^{13}C\{^{1}H\}$ NMR (101 MHz, $CDCl_3$) 1,4-dimethyl-2-phenethylbenzene (**1x**)

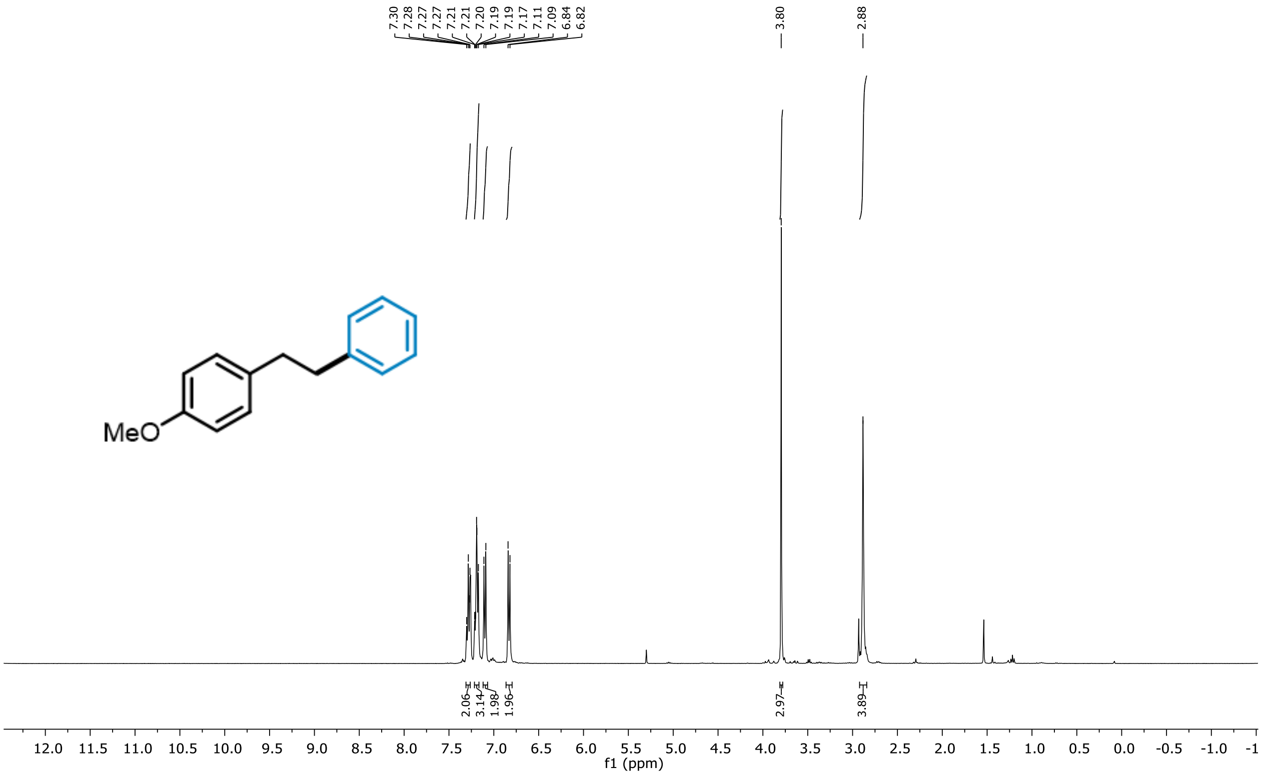

^{1}H NMR (400 MHz, $CDCl_3$) 1-methoxy-4-phenethylbenzene (**1y**)

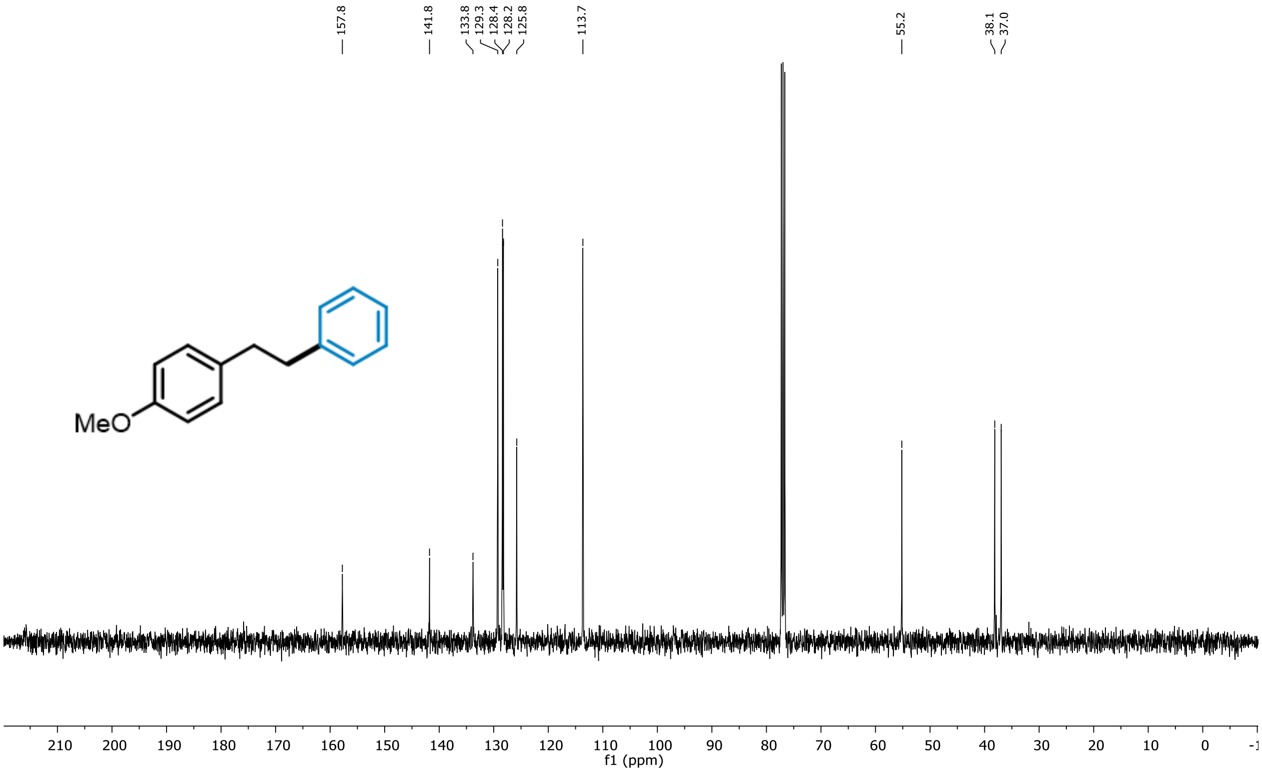

^{13}C{^{1}H} NMR (101 MHz, $CDCl_3$) 1-methoxy-4-phenethylbenzene (**1y**)

^{1}H NMR (400 MHz, $CDCl_3$) diphenylmethane (**2a**) matches with compound **1a**.
$^{13}C\{^{1}H\}$ NMR (101 MHz, $CDCl_3$) diphenylmethane (**2a**) matches with compound **1a**.

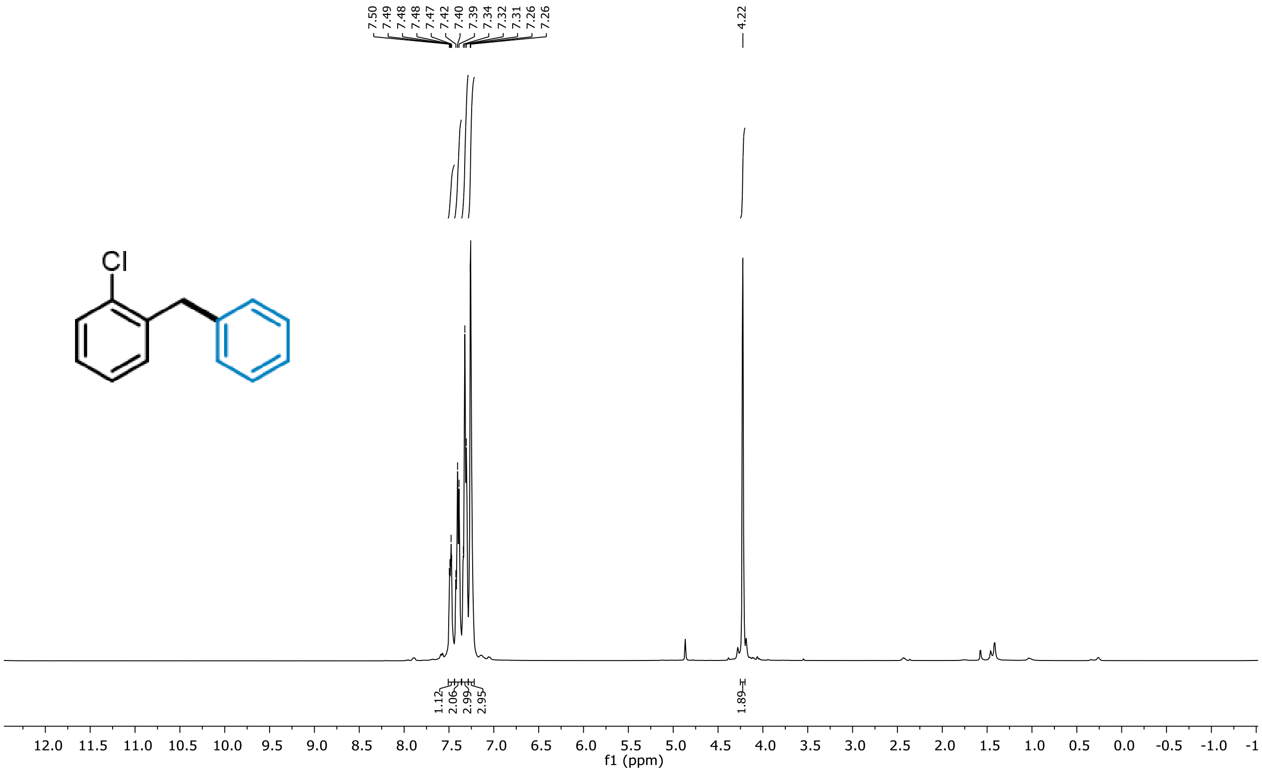

^{1}H NMR (400 MHz, $CDCl_3$) 1-benzyl-2-chlorobenzene (**2b**)

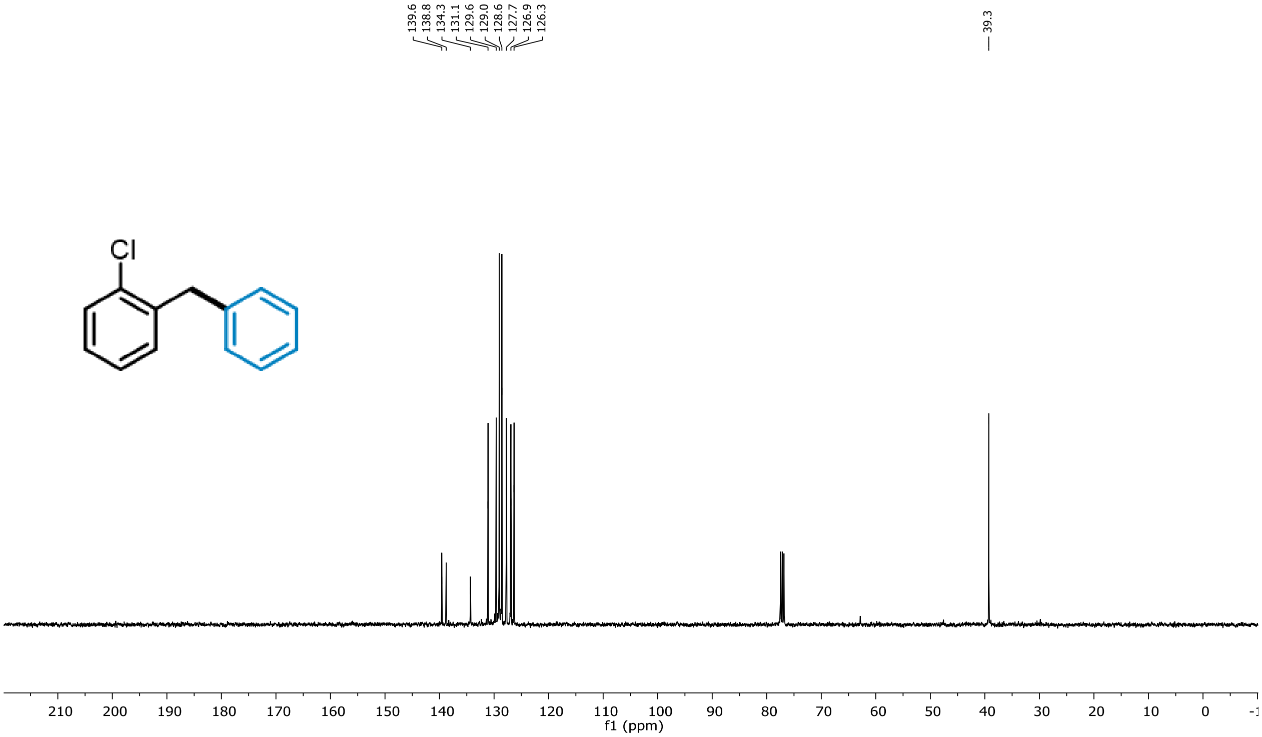

$^{13}C\{^{1}H\}$ NMR (101 MHz, $CDCl_3$) 1-benzyl-2-chlorobenzene (**2b**)

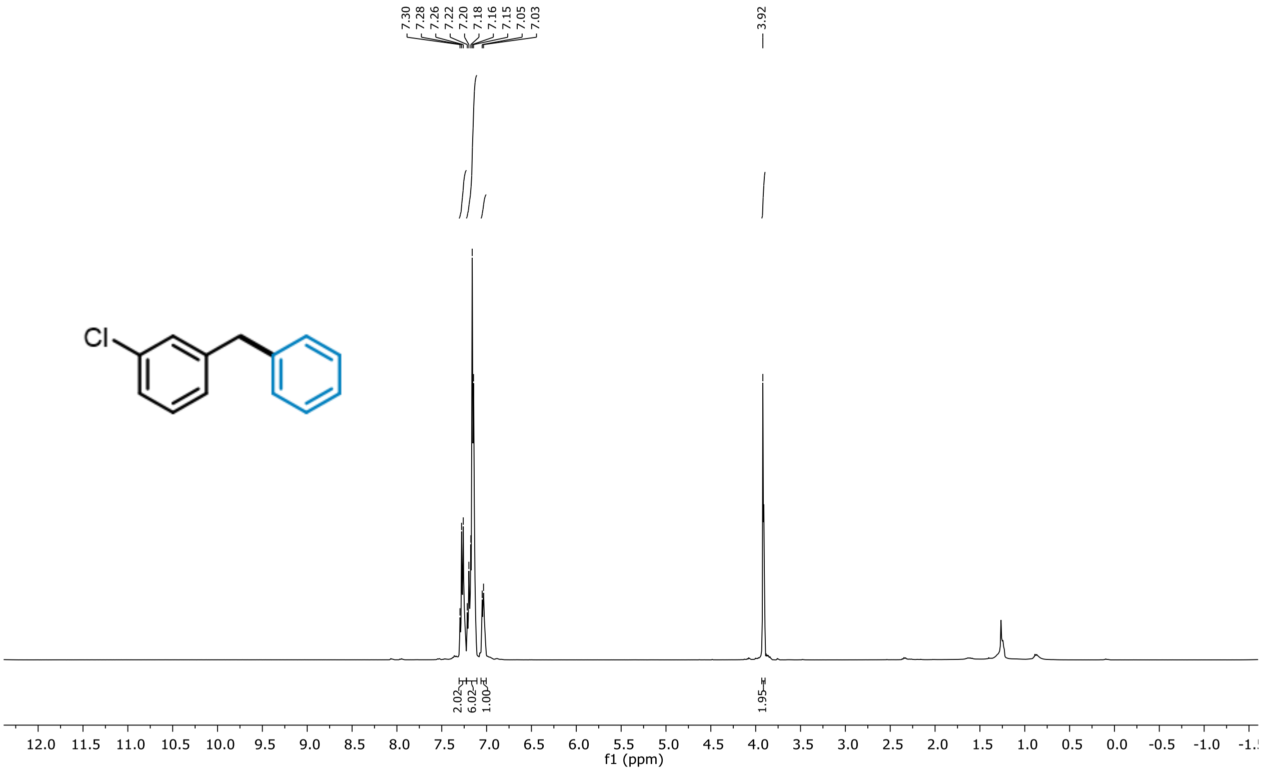

^{1}H NMR (400 MHz, $CDCl_3$) 1-benzyl-3-chlorobenzene (**2c**)

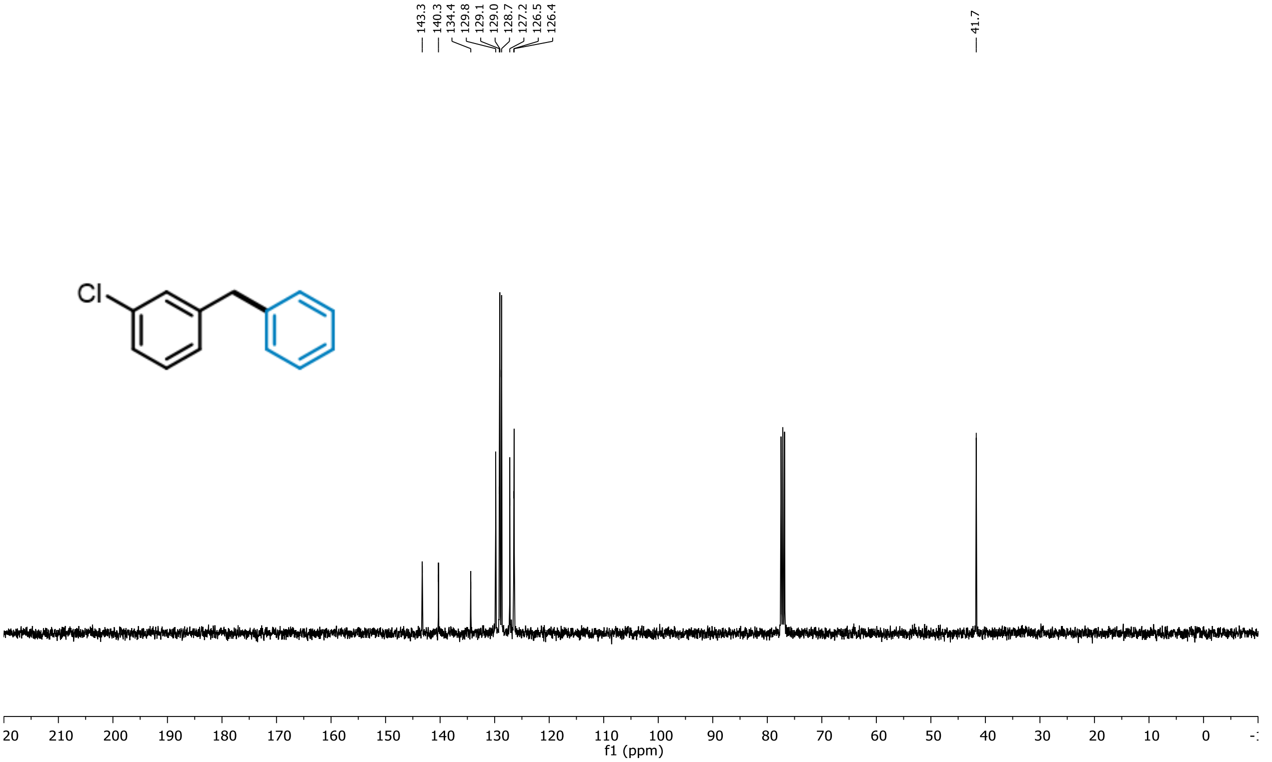

$^{13}C\{^{1}H\}$ NMR (101 MHz, $CDCl_3$) 1-benzyl-3-chlorobenzene (**2c**)

^{1}H NMR (400 MHz, $CDCl_3$) 1-benzyl-4-chlorobenzene (**2d**) matches with compound **1d**.
$^{13}C\{^{1}H\}$ NMR (101 MHz, $CDCl_3$) 1-benzyl-4-chlorobenzene (**2d**) matches with compound **1d**.

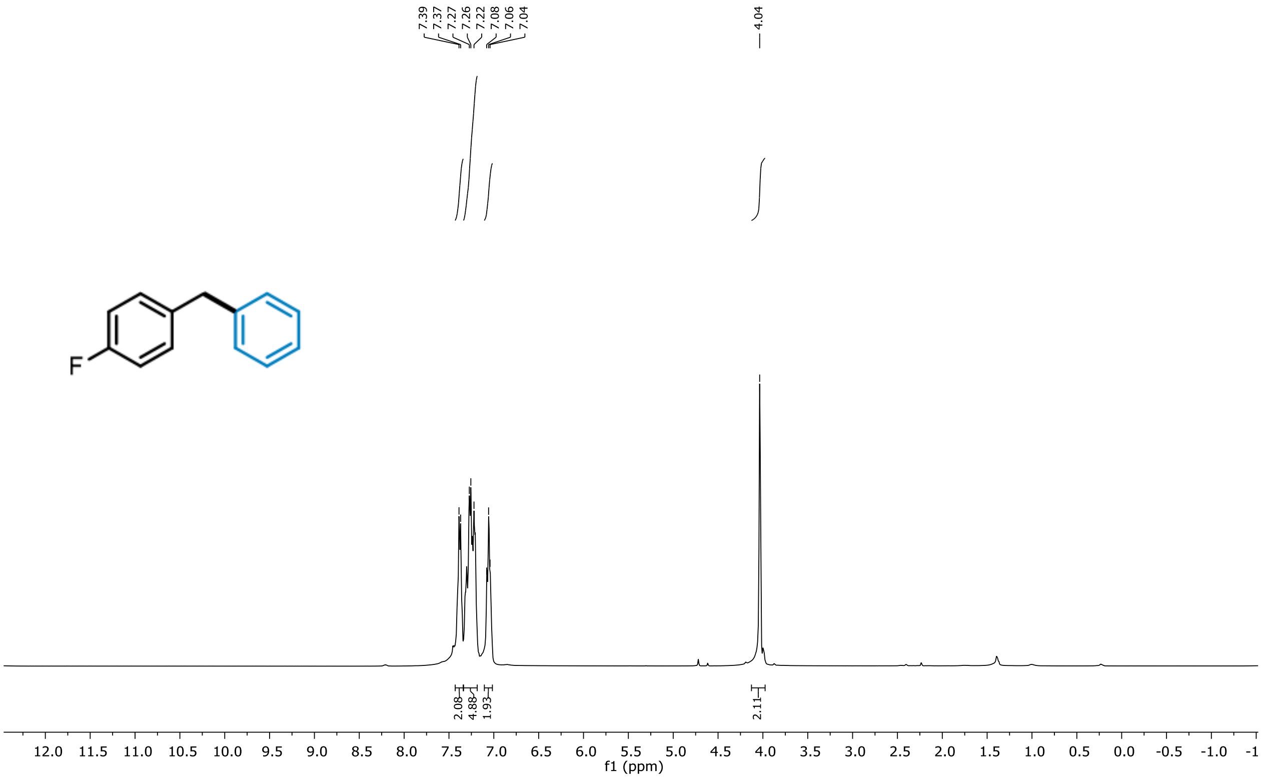

^{1}H NMR (400 MHz, $CDCl_3$) 1-benzyl-4-fluorobenzene (**2e**)

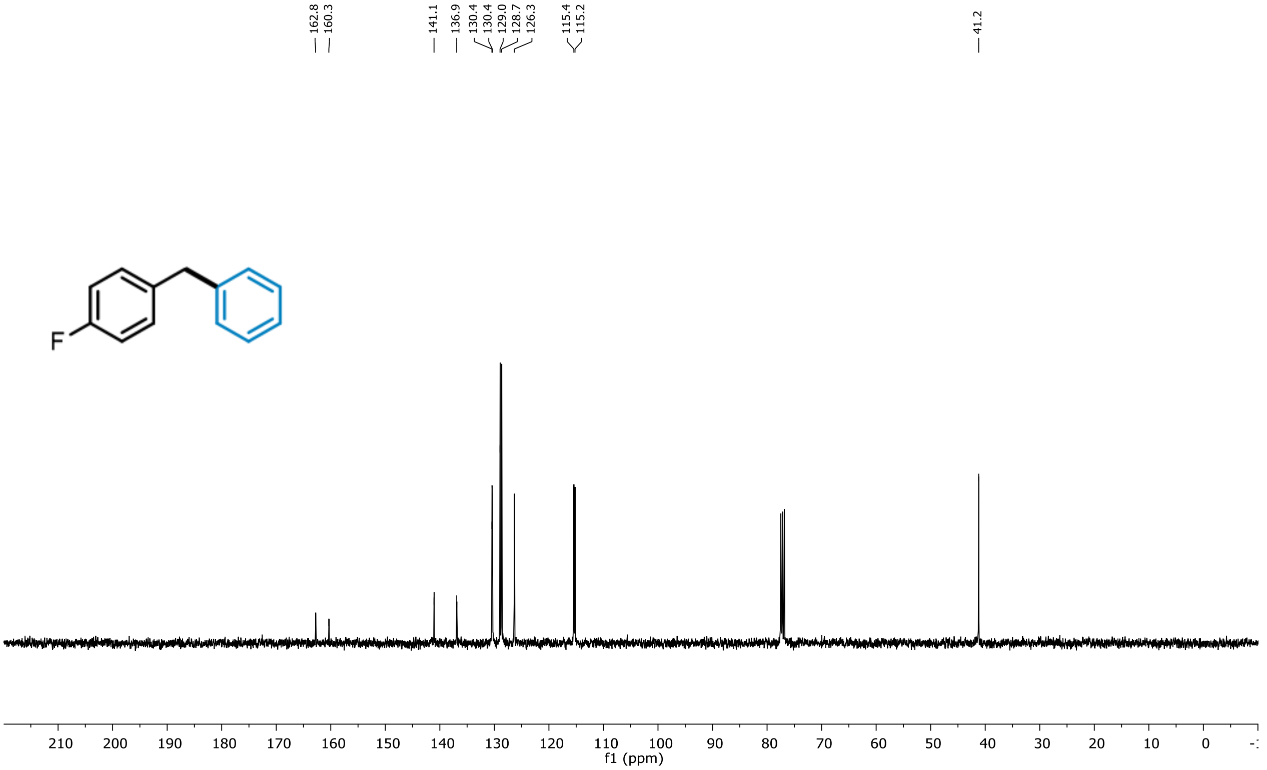

$^{13}C\{^{1}H\}$ NMR (101 MHz, $CDCl_3$) 1-benzyl-4-fluorobenzene (**2e**)

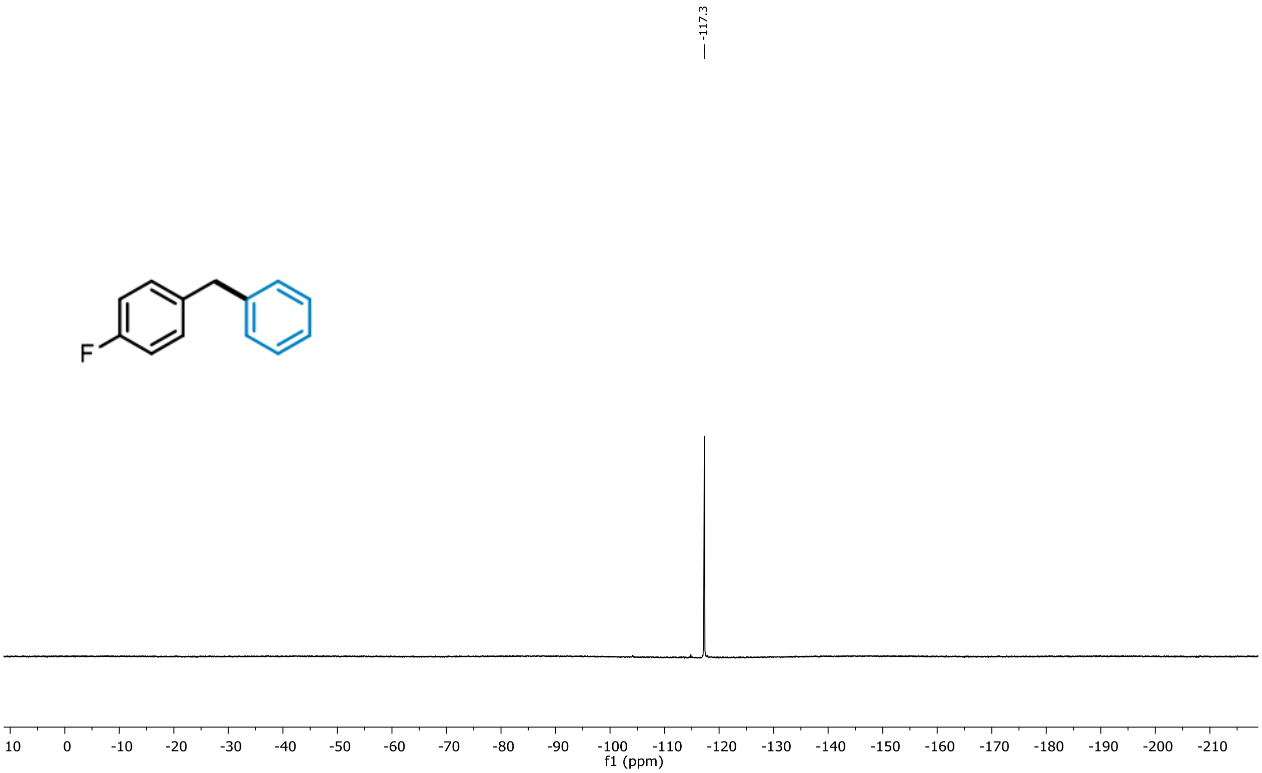

^{19}F NMR (376 MHz, $CDCl_3$) 1-benzyl-4-fluorobenzene (**2e**)

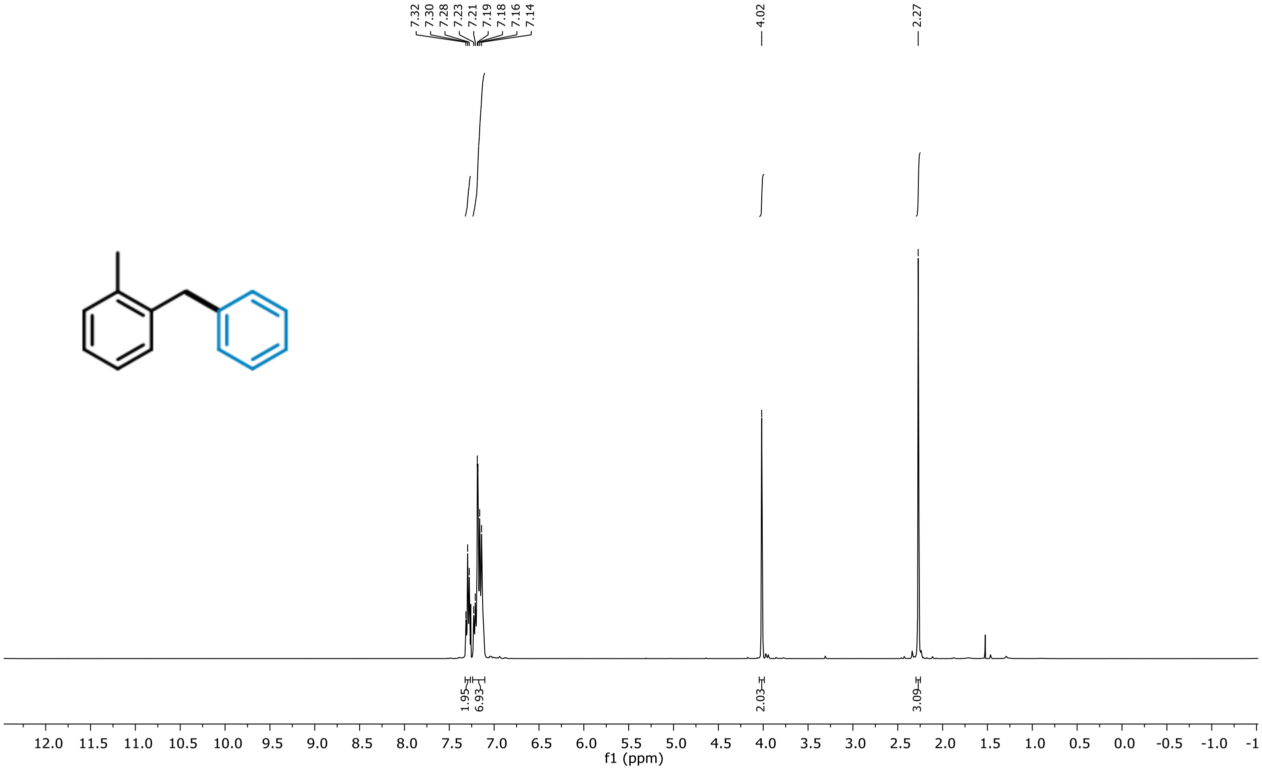

^{1}H NMR (400 MHz, $CDCl_3$) 1-benzyl-2-methylbenzene (**2f**)

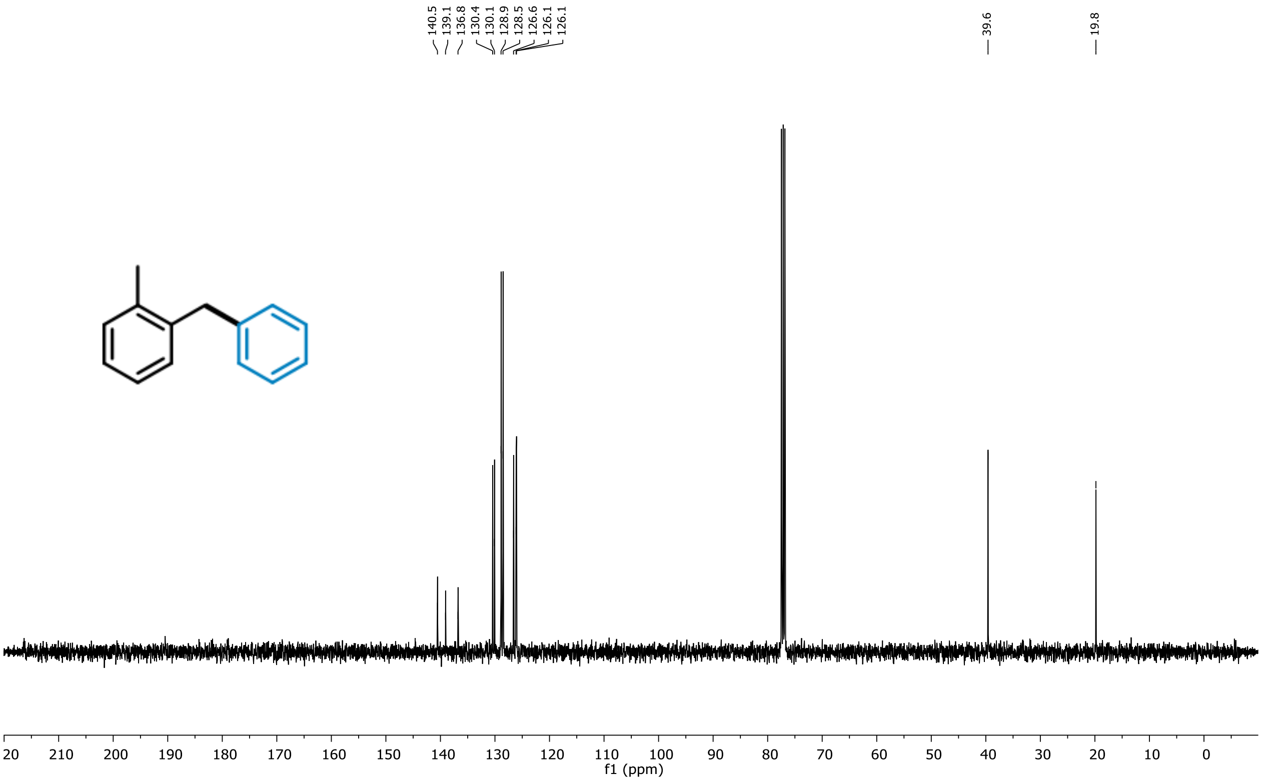

$^{13}C\{^{1}H\}$ NMR (101 MHz, $CDCl_3$) 1-benzyl-2-methylbenzene (**2f**)

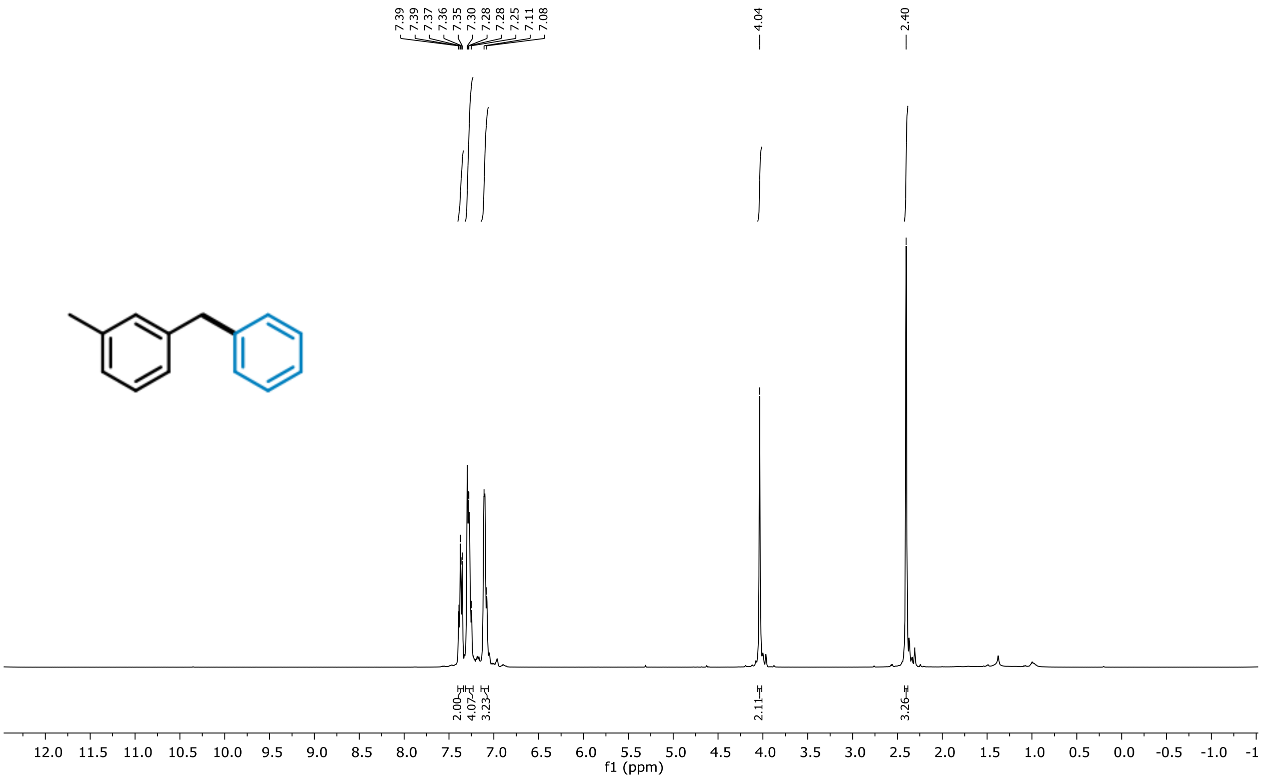

^{1}H NMR (400 MHz, $CDCl_3$) 1-benzyl-3-methylbenzene (**2g**)

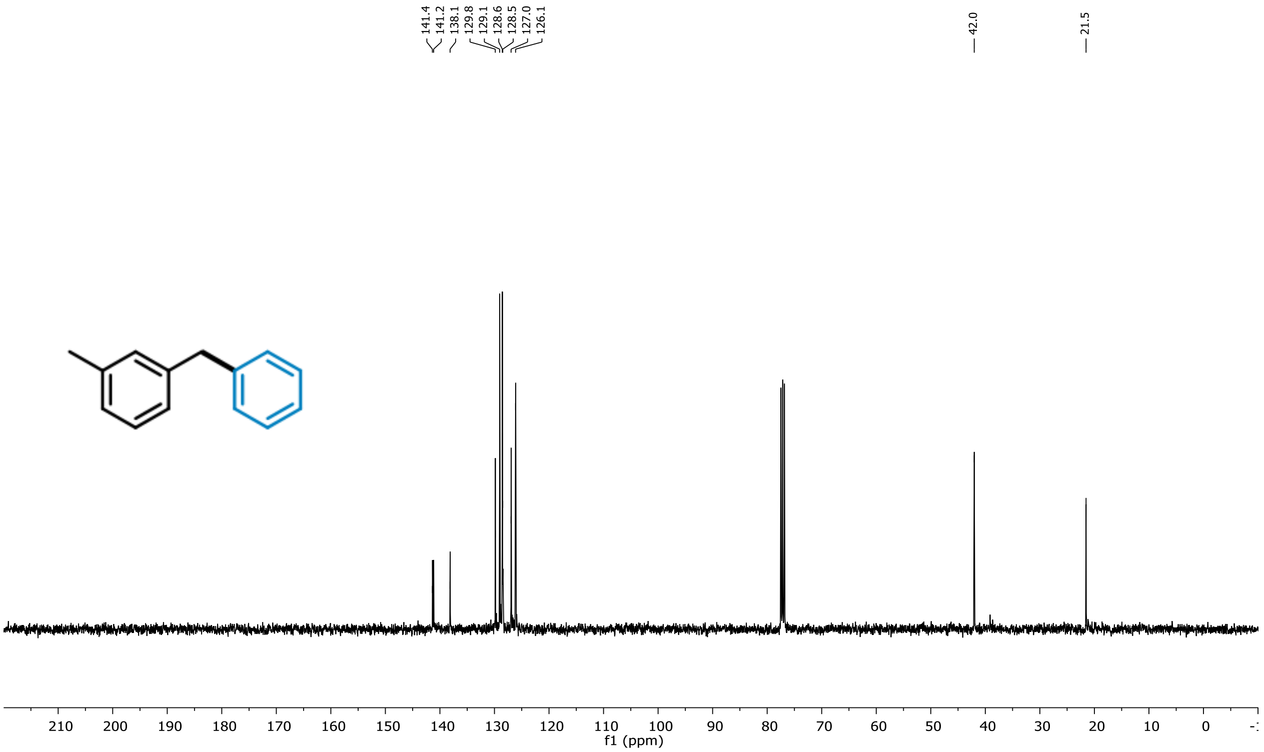

$^{13}C\{^1H\}$ NMR (101 MHz, $CDCl_3$) 1-benzyl-3-methylbenzene (**2g**)

^1H NMR (400 MHz, $CDCl_3$) 1-benzyl-4-methylbenzene (**2h**) matches with compound **1b**.
$^{13}C\{^1H\}$ NMR (101 MHz, $CDCl_3$) 1-benzyl-4-methylbenzene (**2h**) matches with compound **1b**.

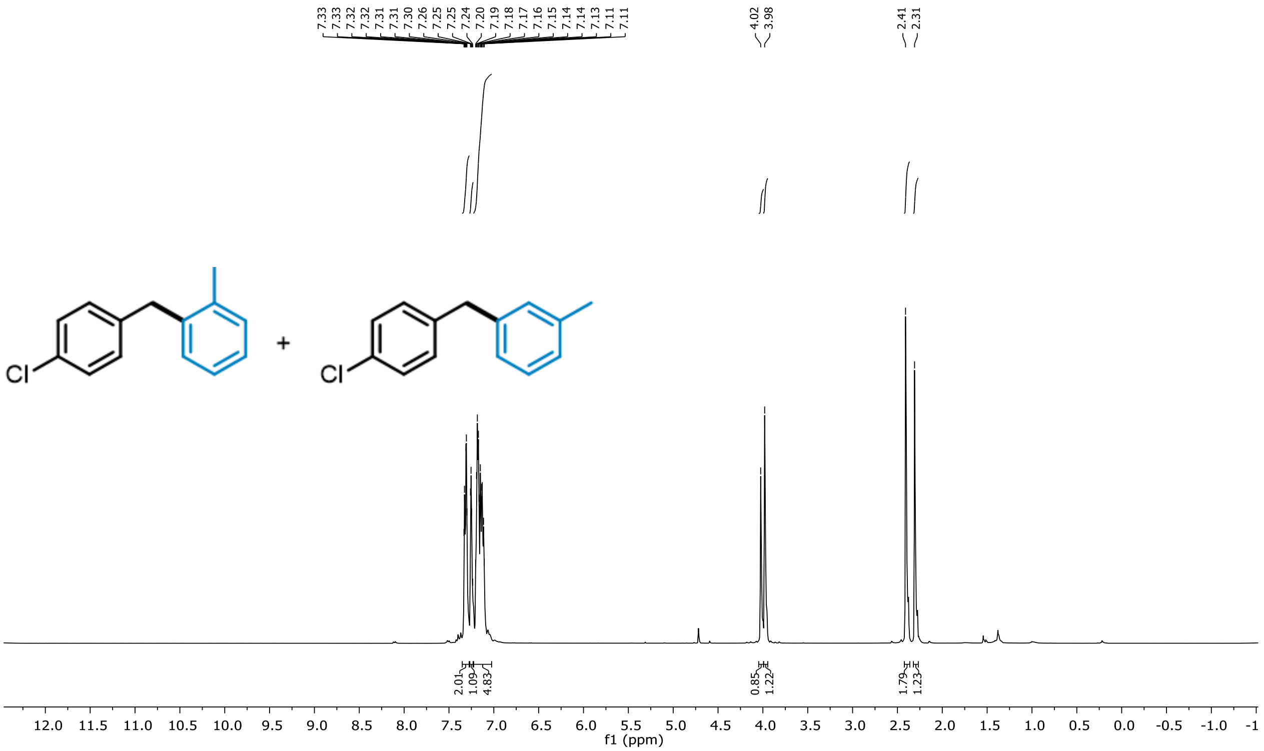

^1H NMR (400 MHz, $CDCl_3$) 1-(4-chlorobenzyl)-2-methylbenzene + 1-(4-chlorobenzyl)-3-methylbenzene (**2i**)

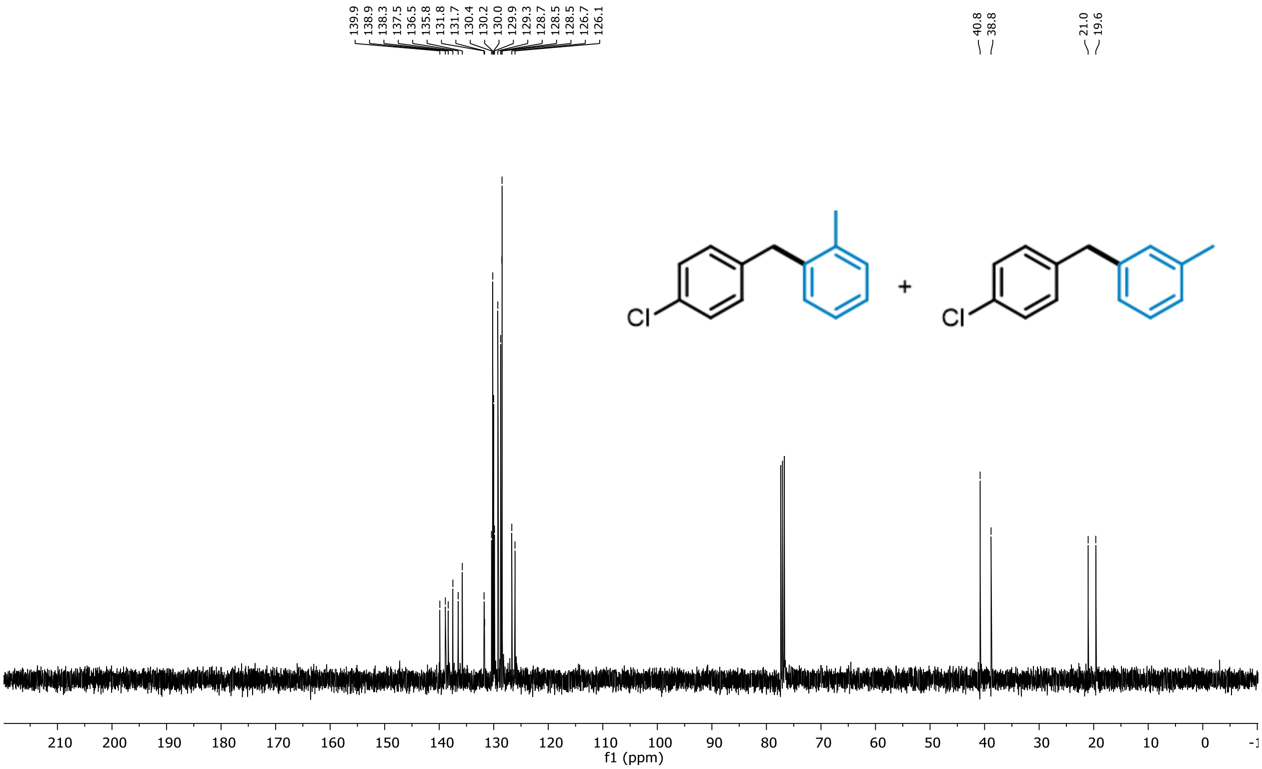

$^{13}C\{^{1}H\}$ NMR (101 MHz, $CDCl_3$) 1-(4-chlorobenzyl)-2-methylbenzene + 1-(4-chlorobenzyl)-3-methylbenzene (**2i**)

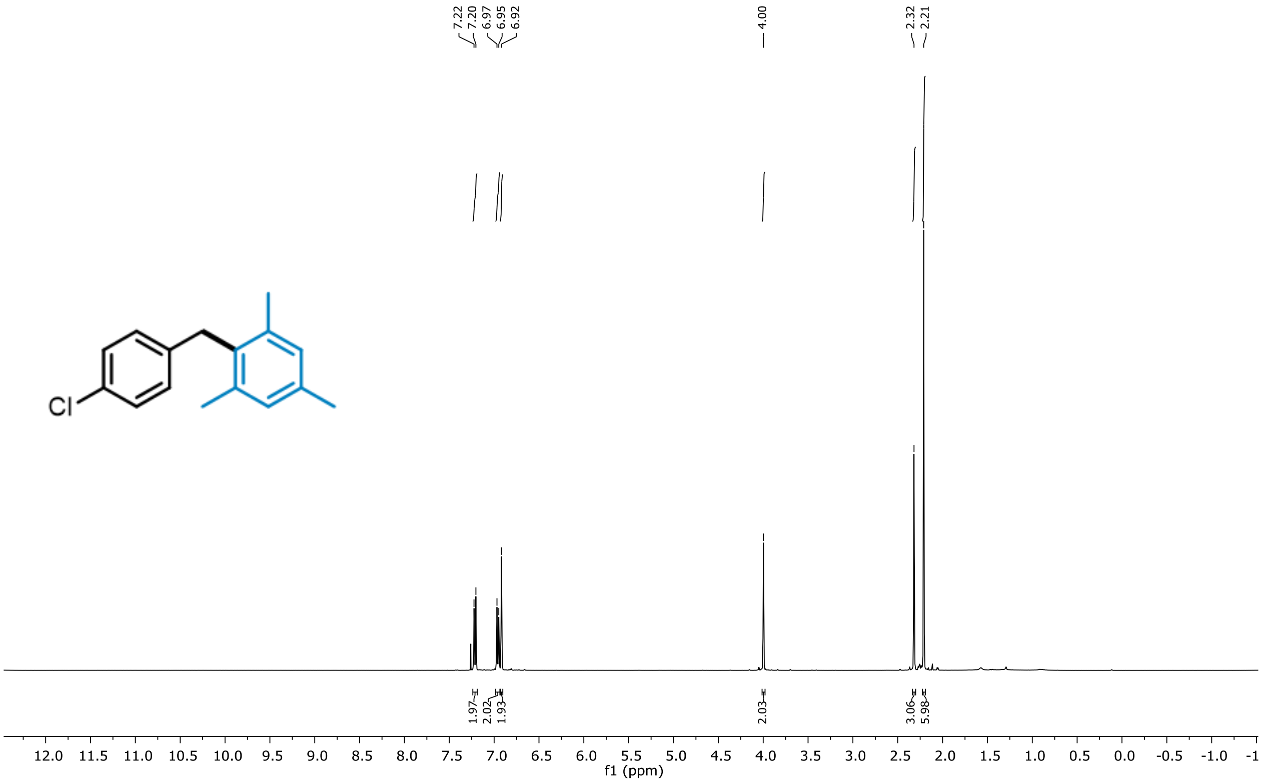

^{1}H NMR (400 MHz, $CDCl_3$) 2-(4-chlorobenzyl)-1,3,5-trimethylbenzene (**2j**)

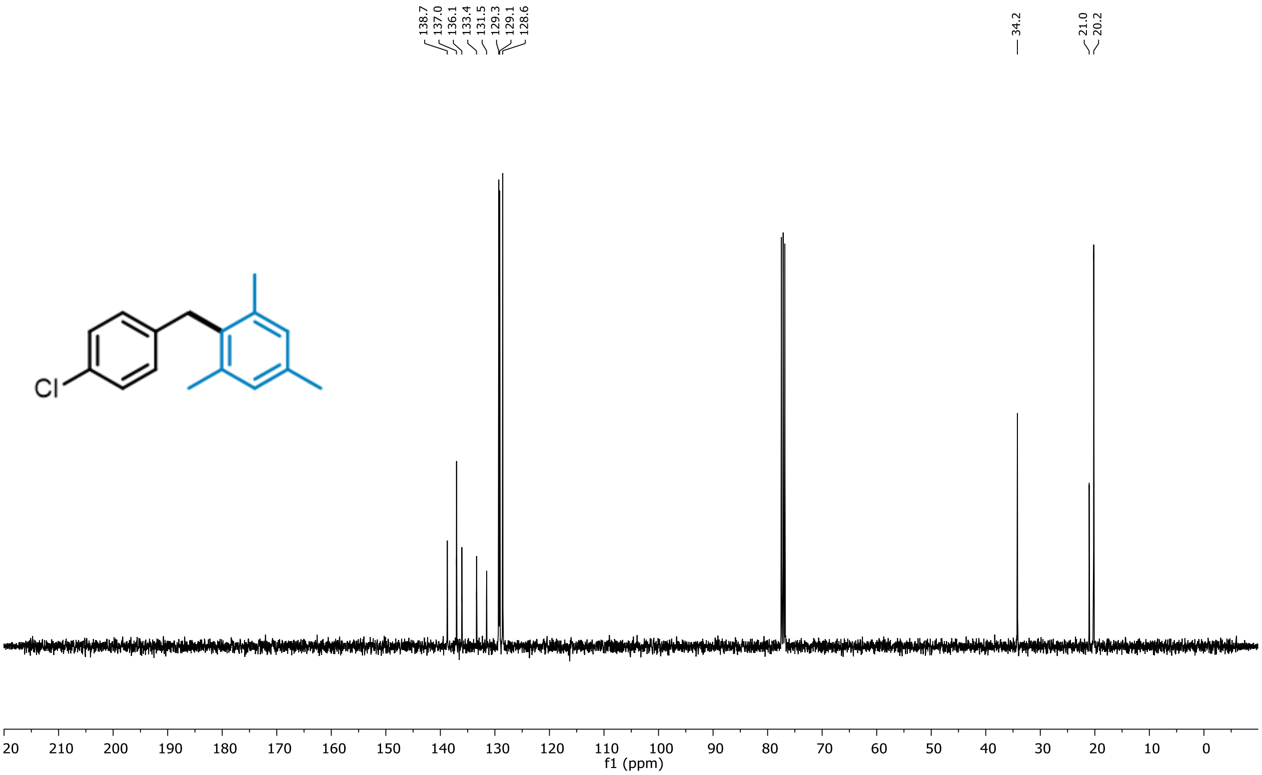

$^{13}C\{^{1}H\}$ NMR (101 MHz, $CDCl_3$) 2-(4-chlorobenzyl)-1,3,5-trimethylbenzene (**2j**)

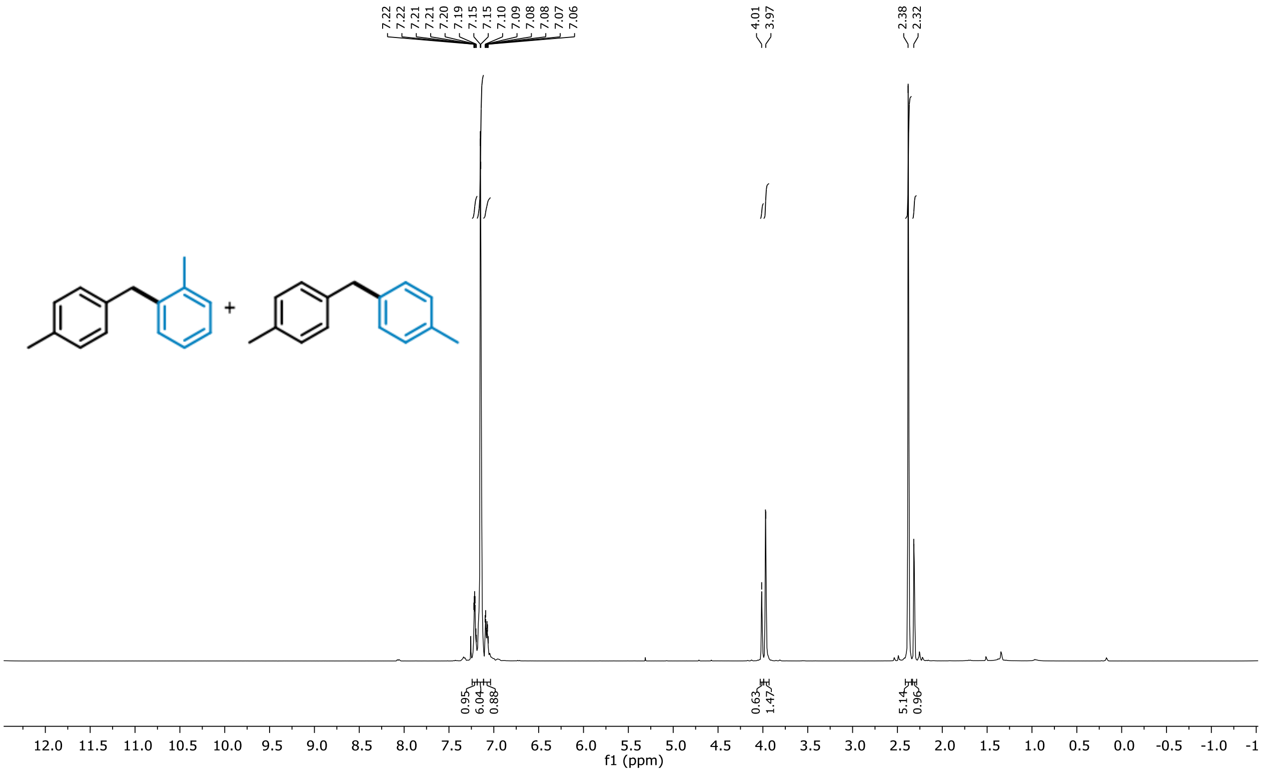

^{1}H NMR (400 MHz, $CDCl_3$) 1-methyl-2-(4-methylbenzyl)benzene + 1-methyl-3-(4-methylbenzyl)benzene (**2k**)

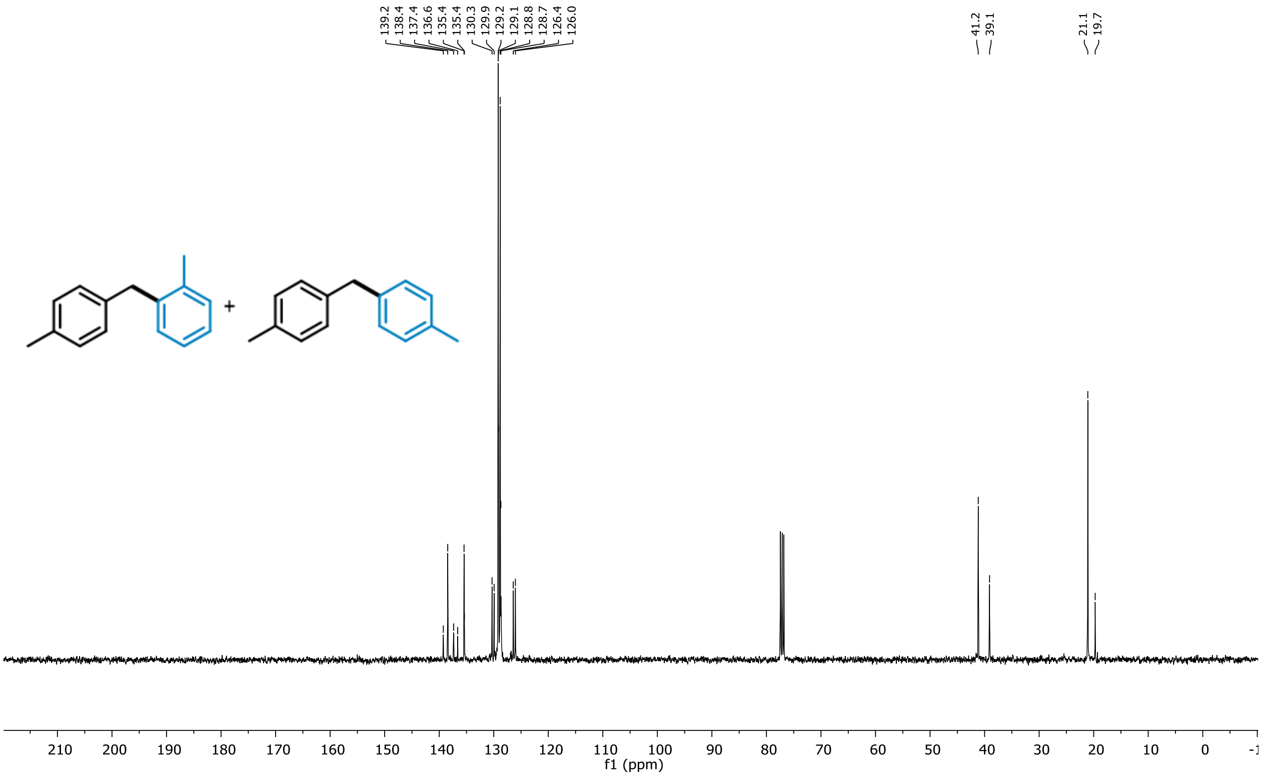

$^{13}C\{^1H\}$ NMR (101 MHz, $CDCl_3$) 1-methyl-2-(4-methylbenzyl)benzene + 1-methyl-3-(4-methylbenzyl)benzene (**2k**)

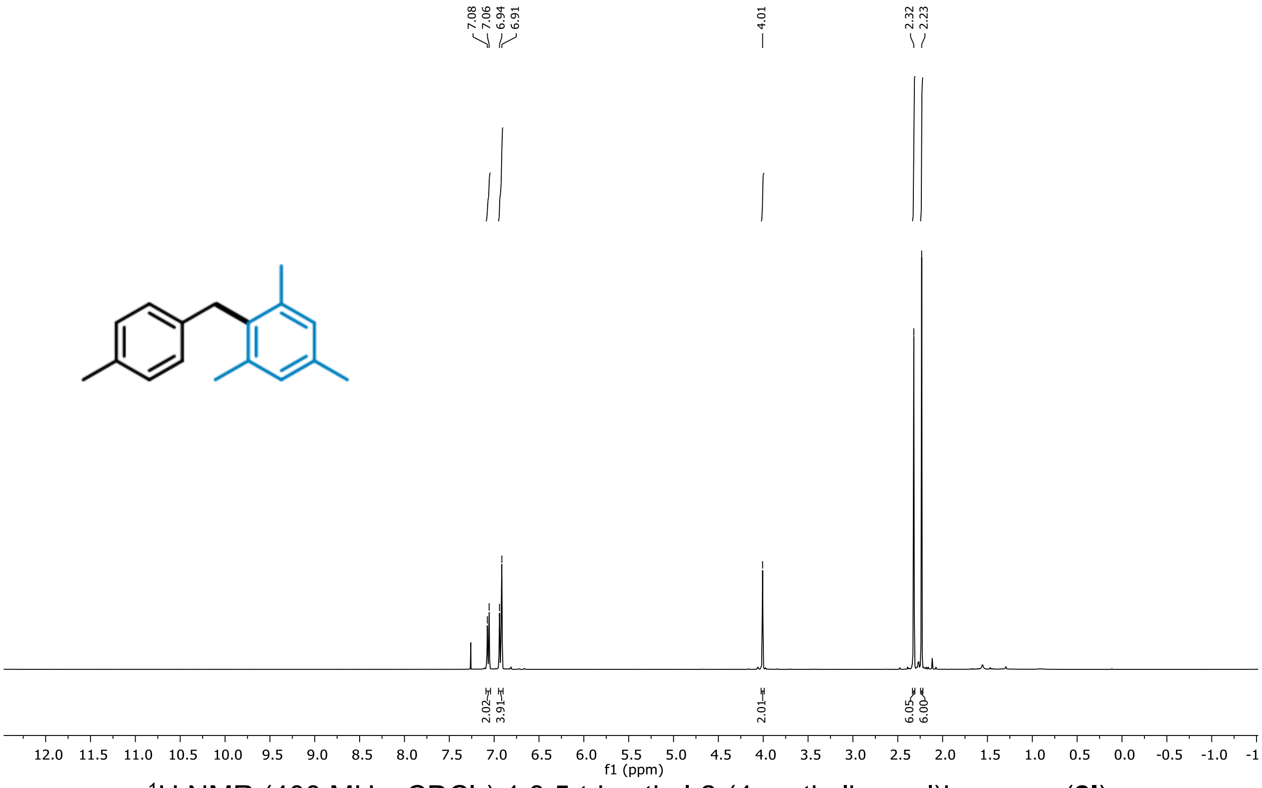

^1H NMR (400 MHz, $CDCl_3$) 1,3,5-trimethyl-2-(4-methylbenzyl)benzene (**2l**)

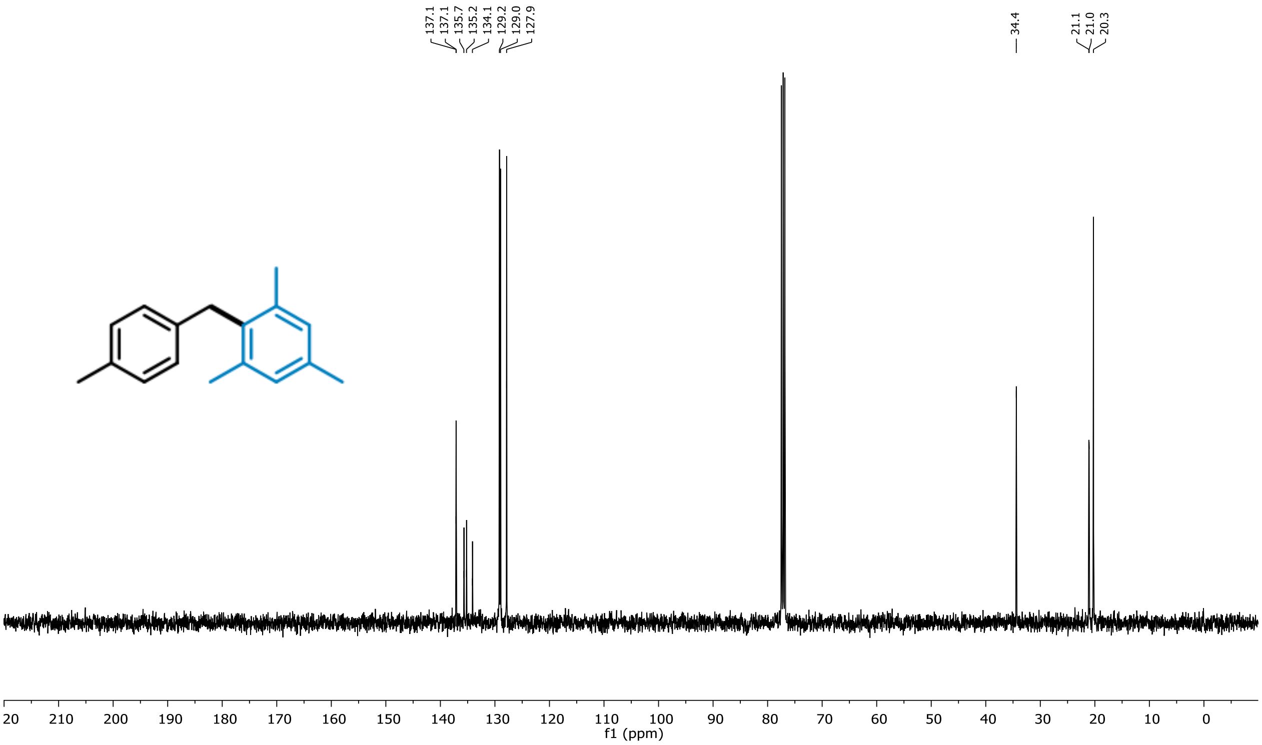

$^{13}C\{^1H\}$ NMR (101 MHz, $CDCl_3$) 1,3,5-trimethyl-2-(4-methylbenzyl)benzene (**2l**)

^1H NMR (400 MHz, $CDCl_3$) ethane-1,1-diyldibenzene (**3a**) matches with compound **1h**.
$^{13}C\{^1H\}$ NMR (101 MHz, $CDCl_3$) ethane-1,1-diyldibenzene (**3a**) matches with compound **1h**.

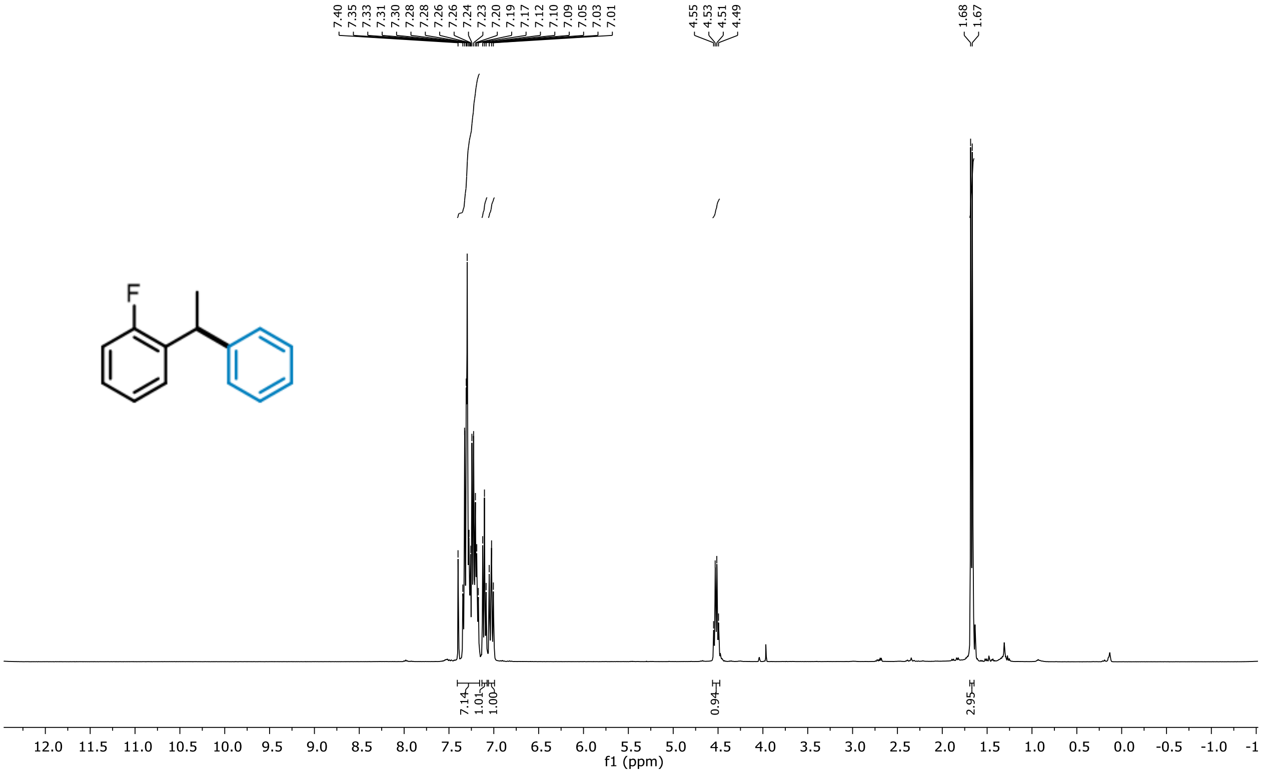

^1H NMR (400 MHz, $CDCl_3$) 1-fluoro-2-(1-phenylethyl)benzene (**3b**)

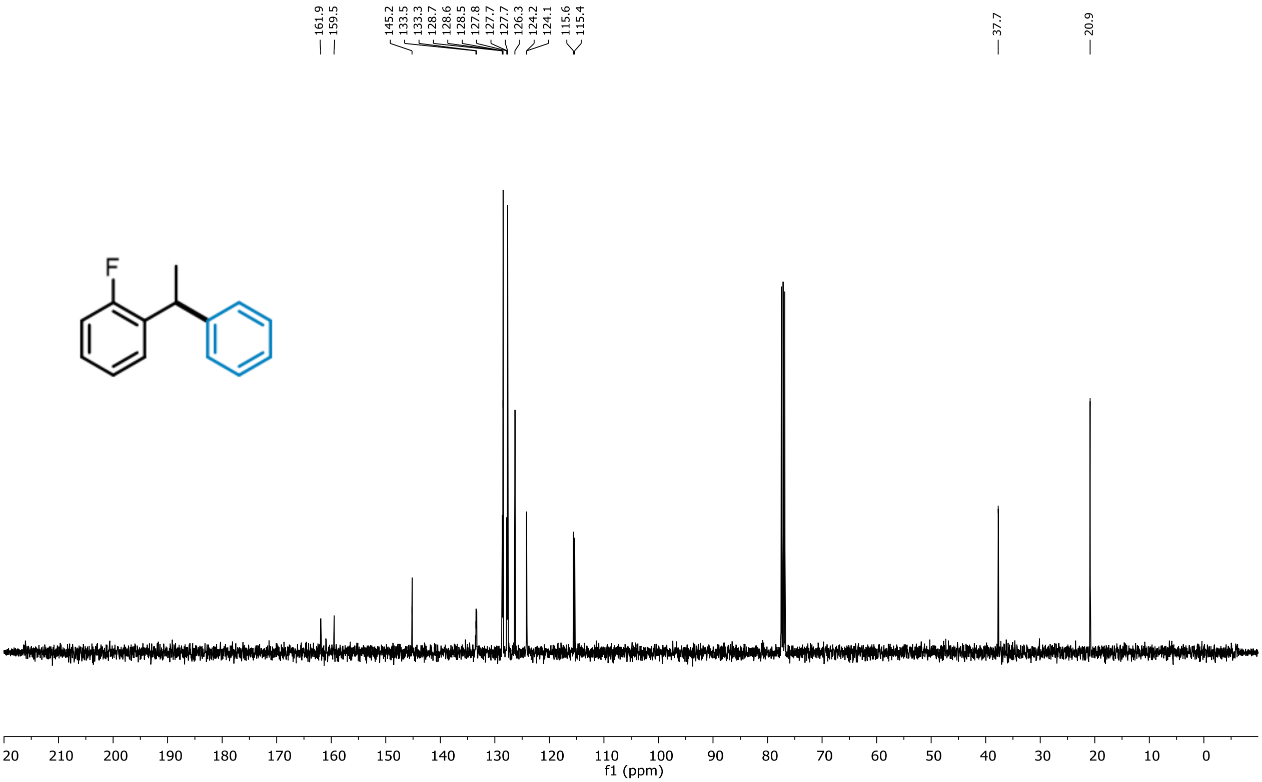

$^{13}C\{^1H\}$ NMR (101 MHz, $CDCl_3$) 1-fluoro-2-(1-phenylethyl)benzene (**3b**)

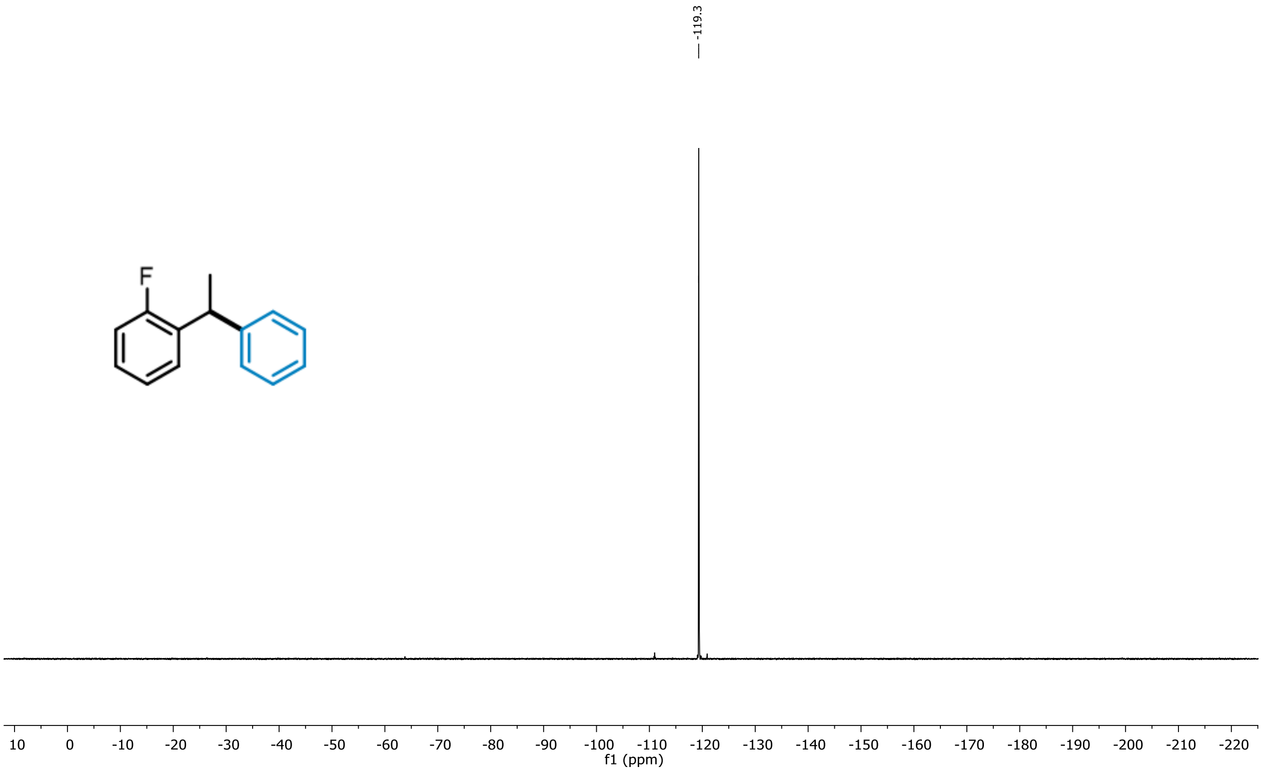

^{19}F NMR (376 MHz, $CDCl_3$) 1-fluoro-2-(1-phenylethyl)benzene (**3b**)

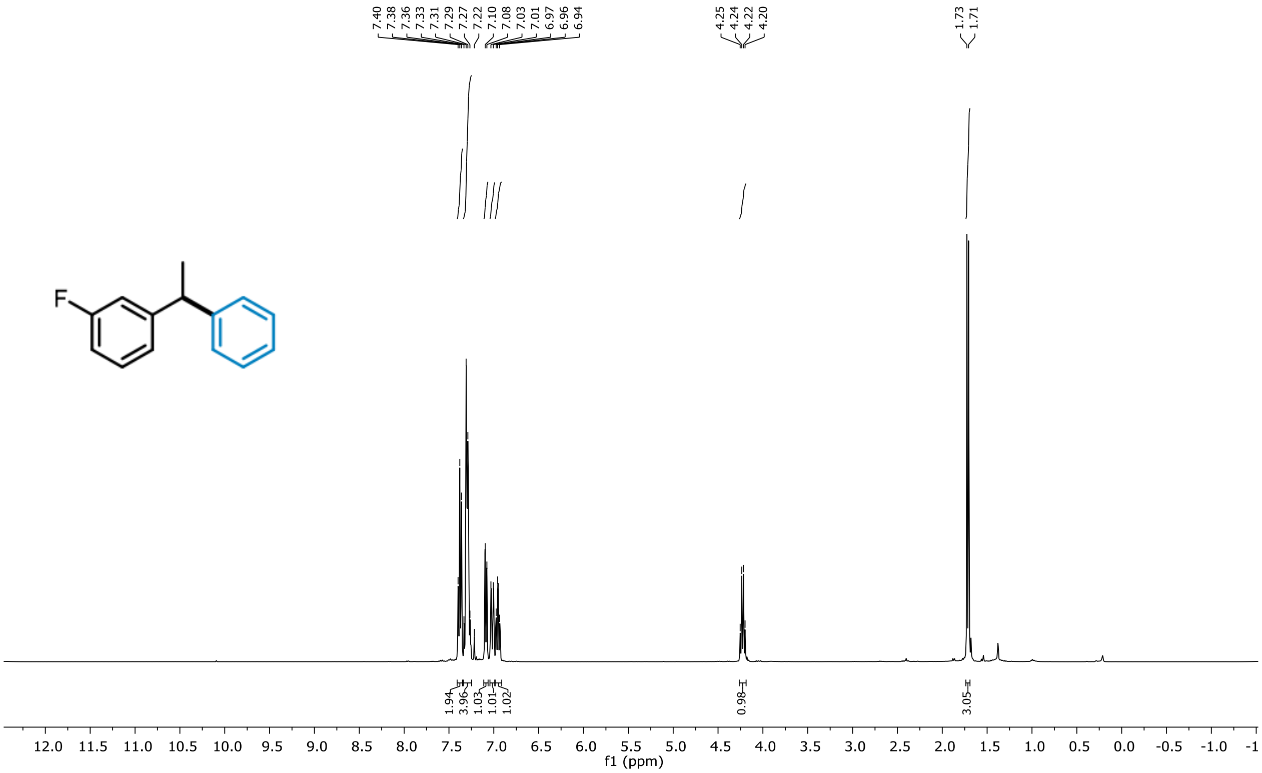

^{1}H NMR (400 MHz, $CDCl_3$) 1-fluoro-3-(1-phenylethyl)benzene (**3c**)

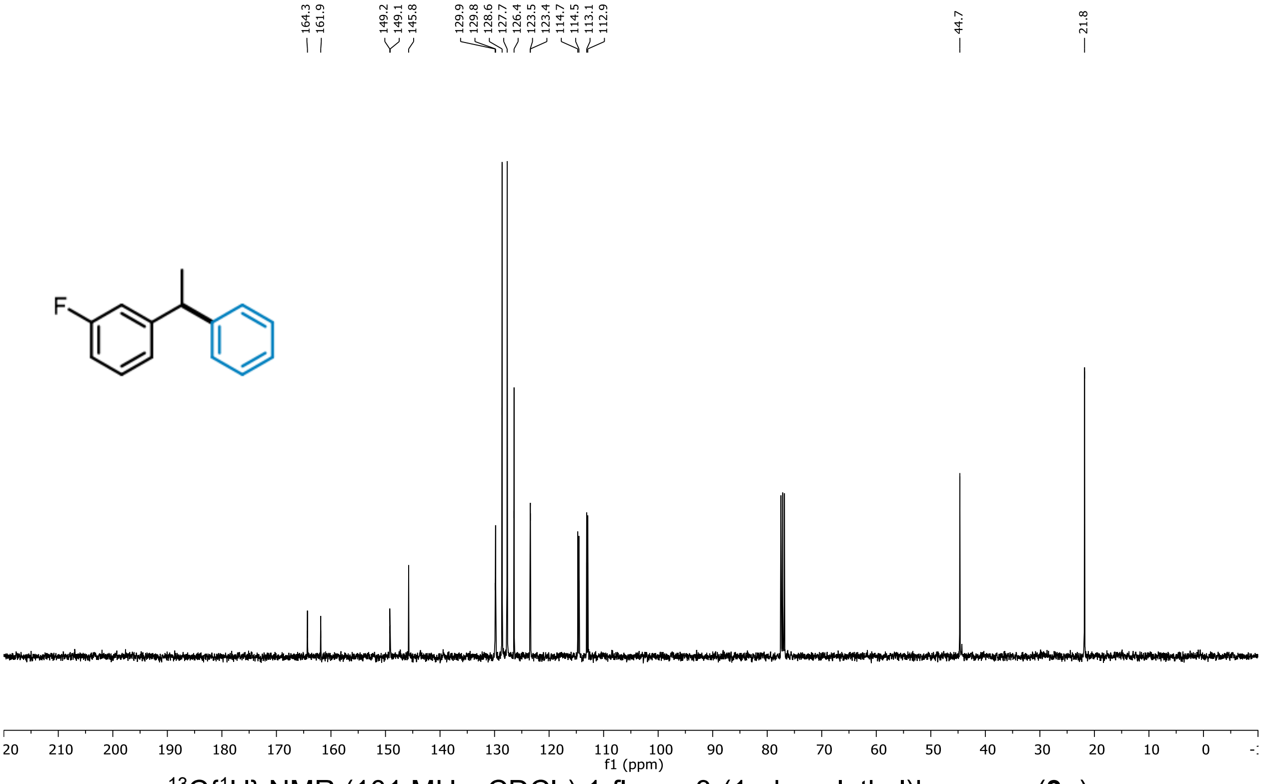

$^{13}C\{^1H\}$ NMR (101 MHz, $CDCl_3$) 1-fluoro-3-(1-phenylethyl)benzene (**3c**)

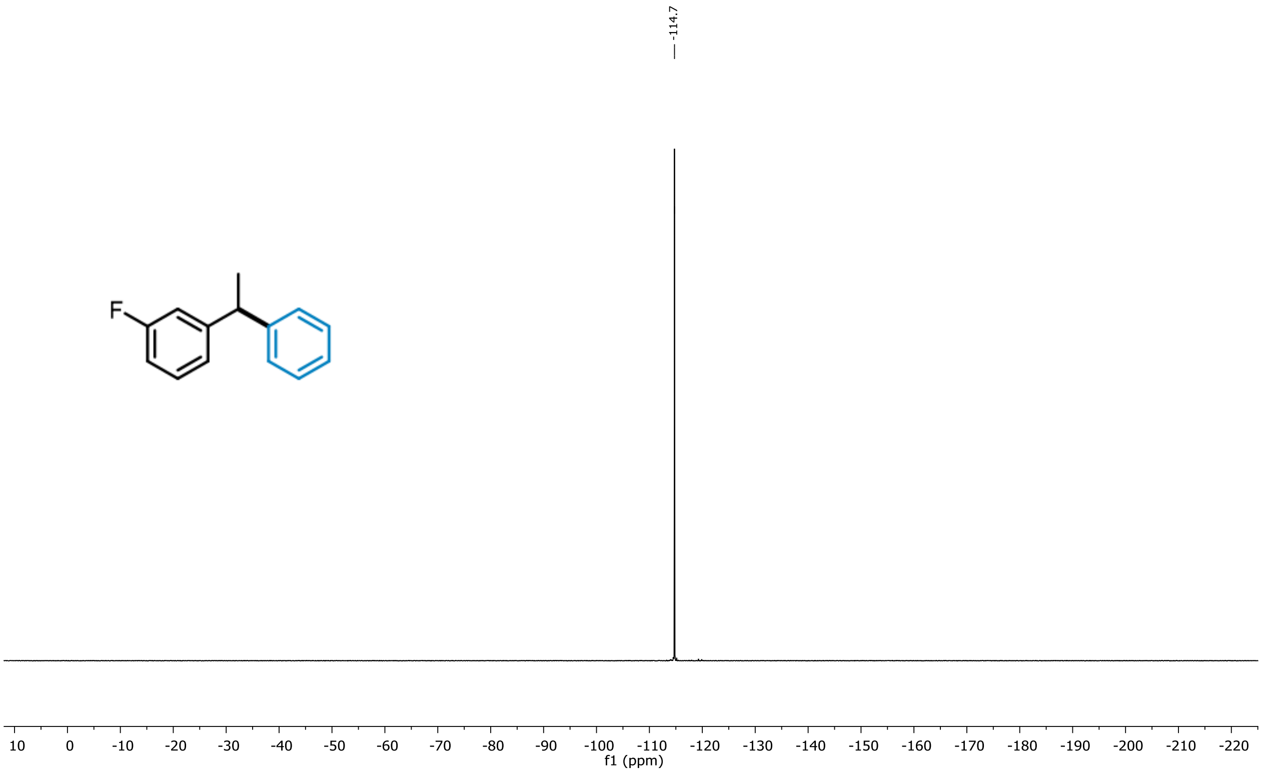

^{19}F NMR (376 MHz, $CDCl_3$) 1-fluoro-3-(1-phenylethyl)benzene (**3c**)

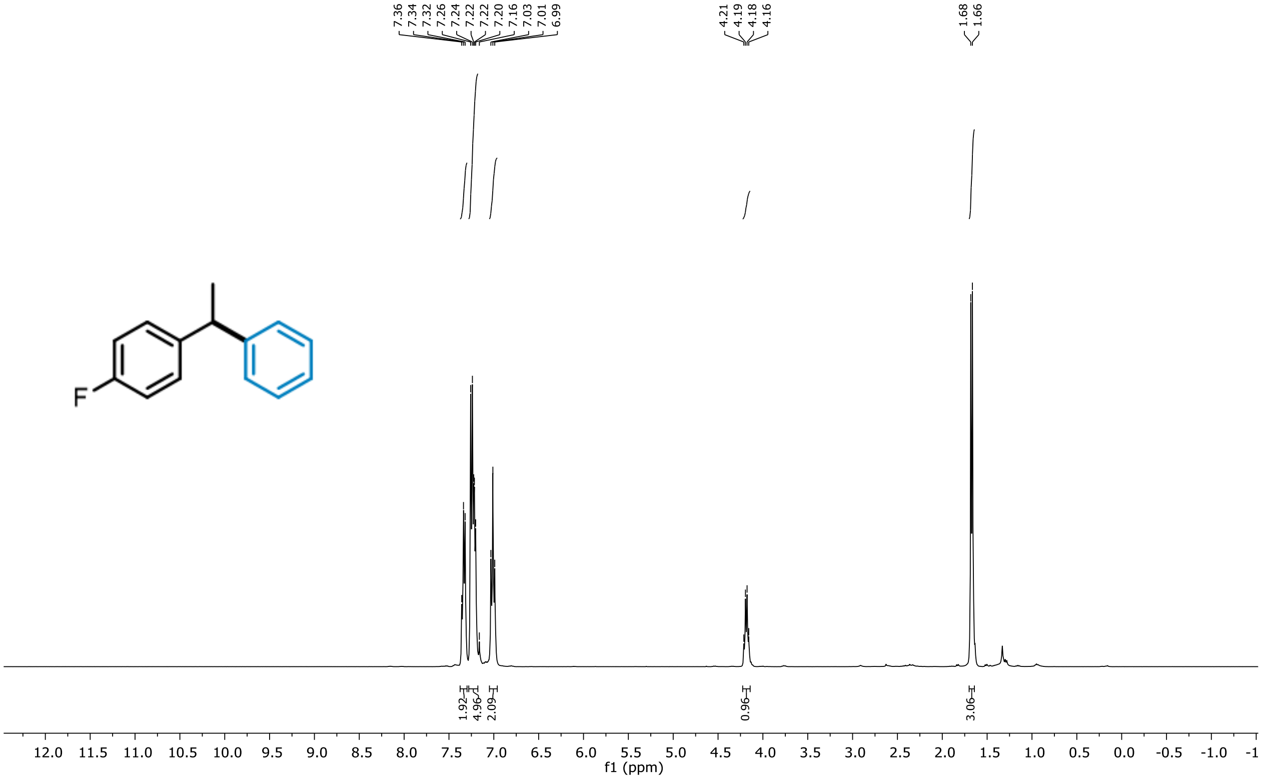

^{1}H NMR (400 MHz, $CDCl_3$) 1-fluoro-4-(1-phenylethyl)benzene (**3d**)

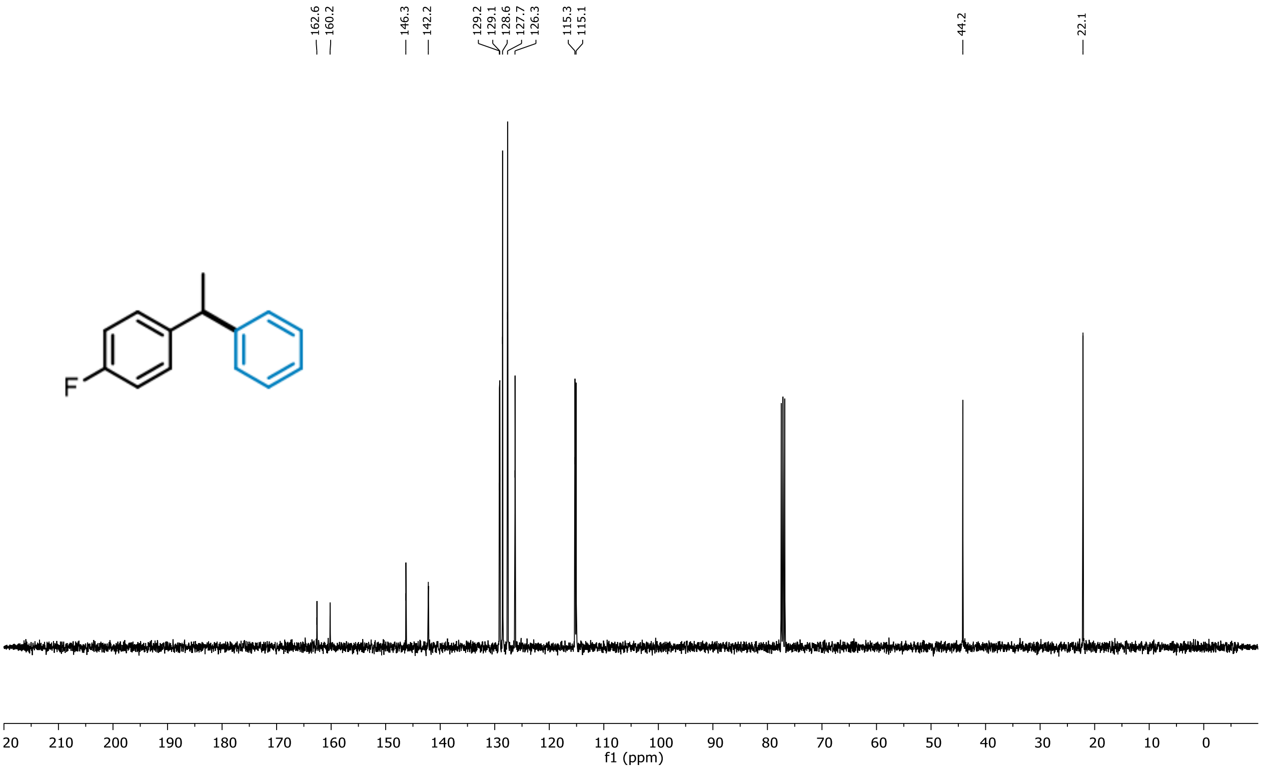

$^{13}C\{^{1}H\}$ NMR (101 MHz, $CDCl_3$) 1-fluoro-4-(1-phenylethyl)benzene (**3d**)

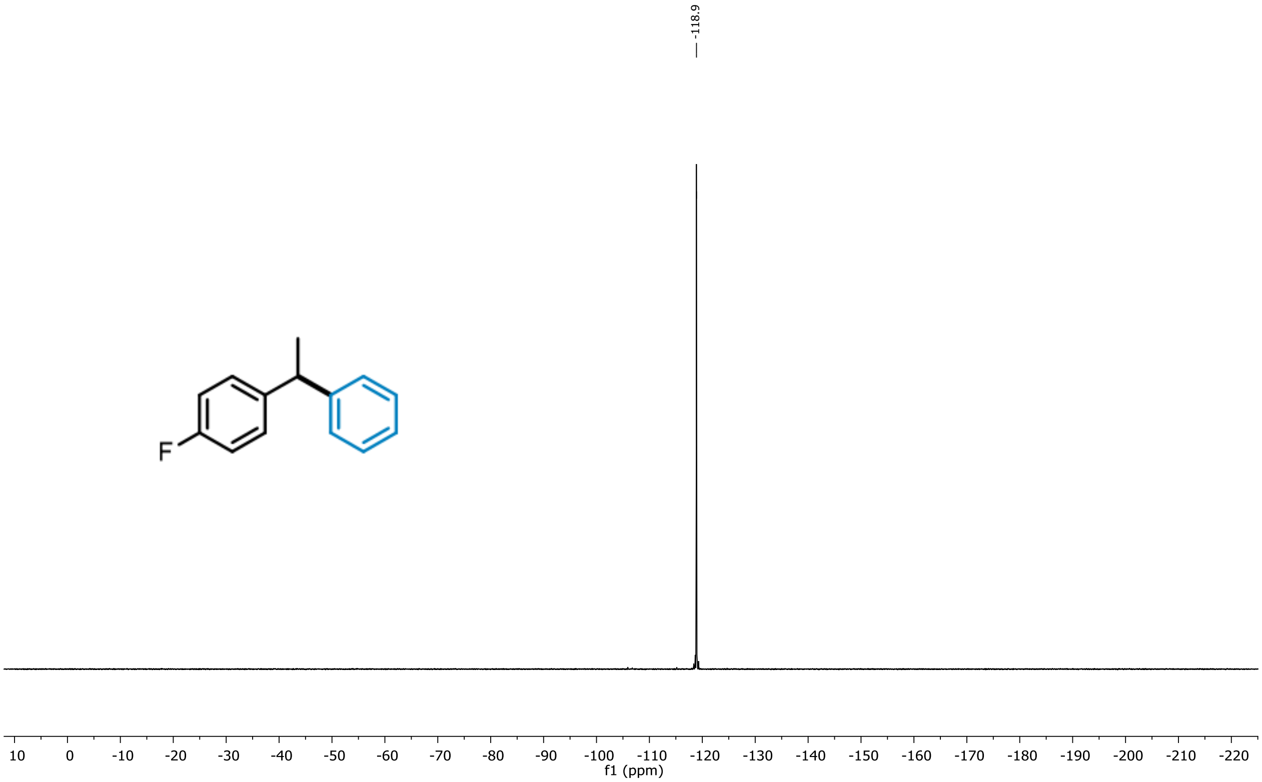

^{19}F NMR (376 MHz, $CDCl_3$) 1-fluoro-4-(1-phenylethyl)benzene (**3d**)

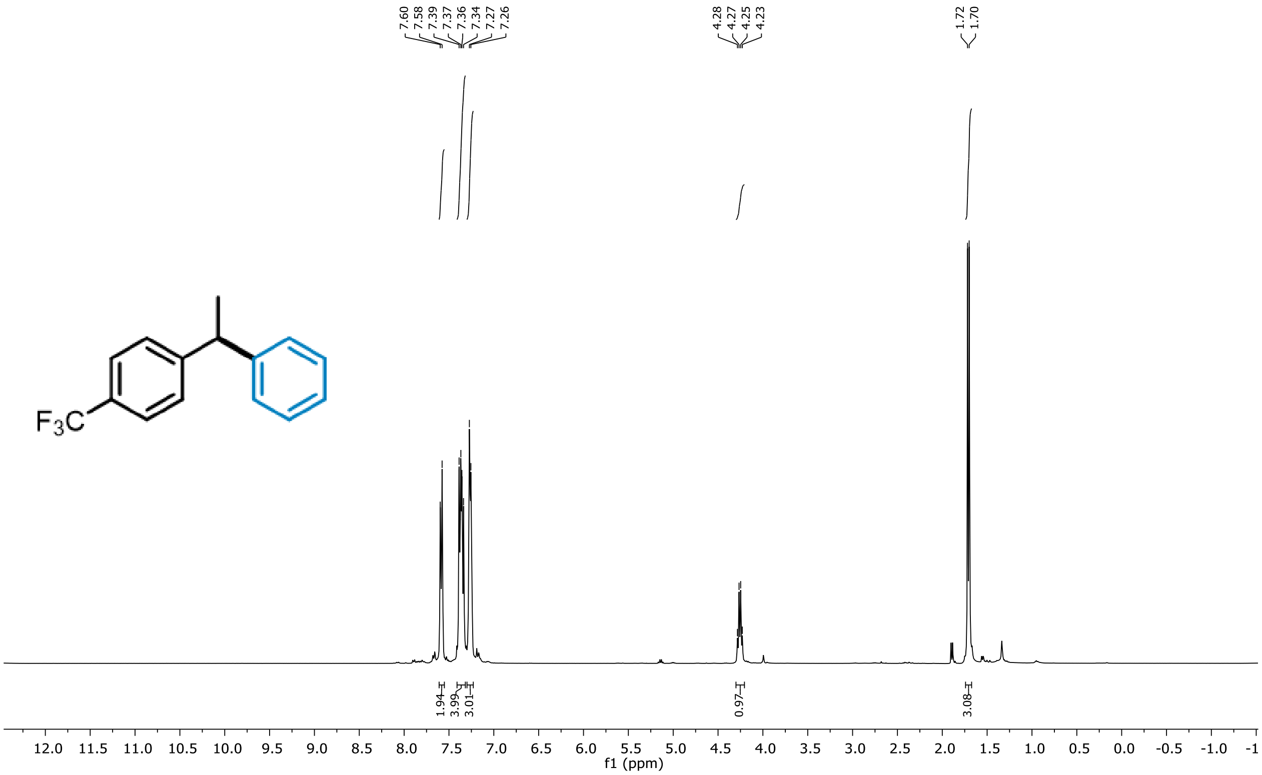

^{1}H NMR (400 MHz, $CDCl_3$) 1-(1-phenylethyl)-4-(trifluoromethyl)benzene (**3e**)

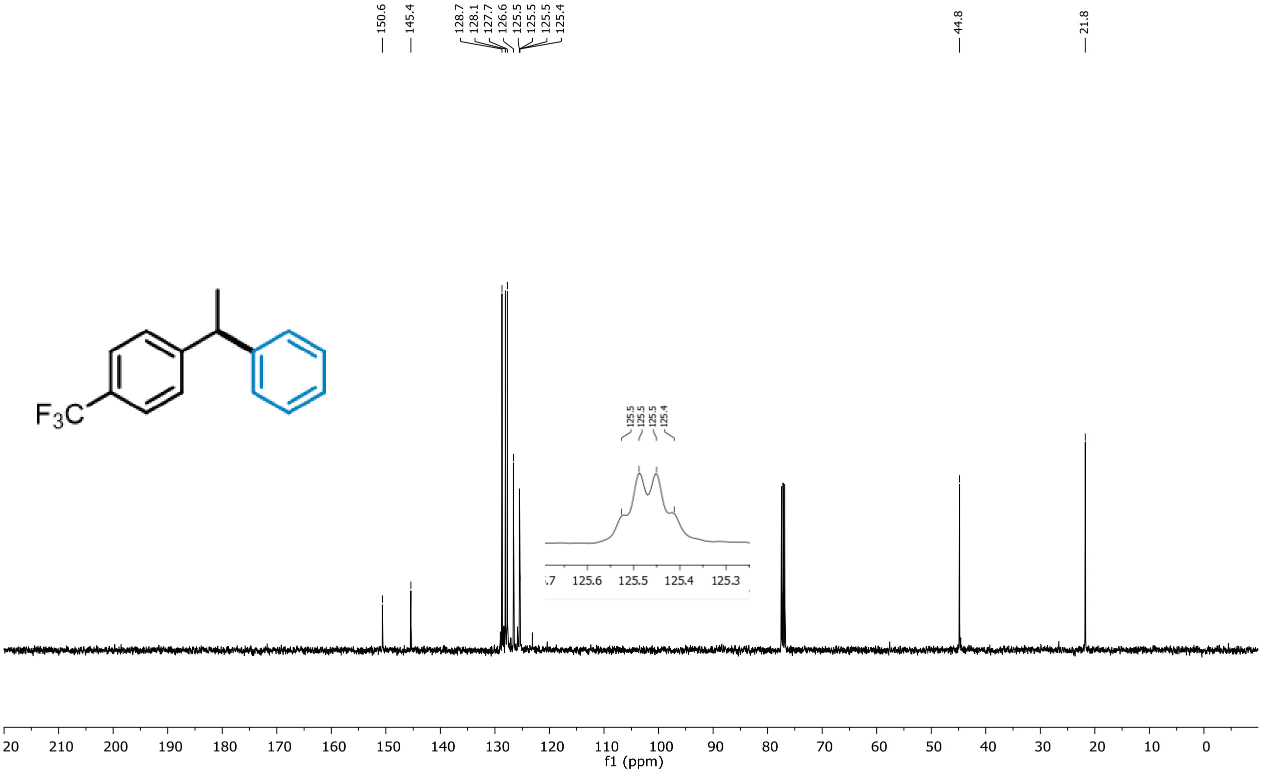

$^{13}C\{^{1}H\}$ NMR (101 MHz, $CDCl_3$) 1-(1-phenylethyl)-4-(trifluoromethyl)benzene (**3e**)

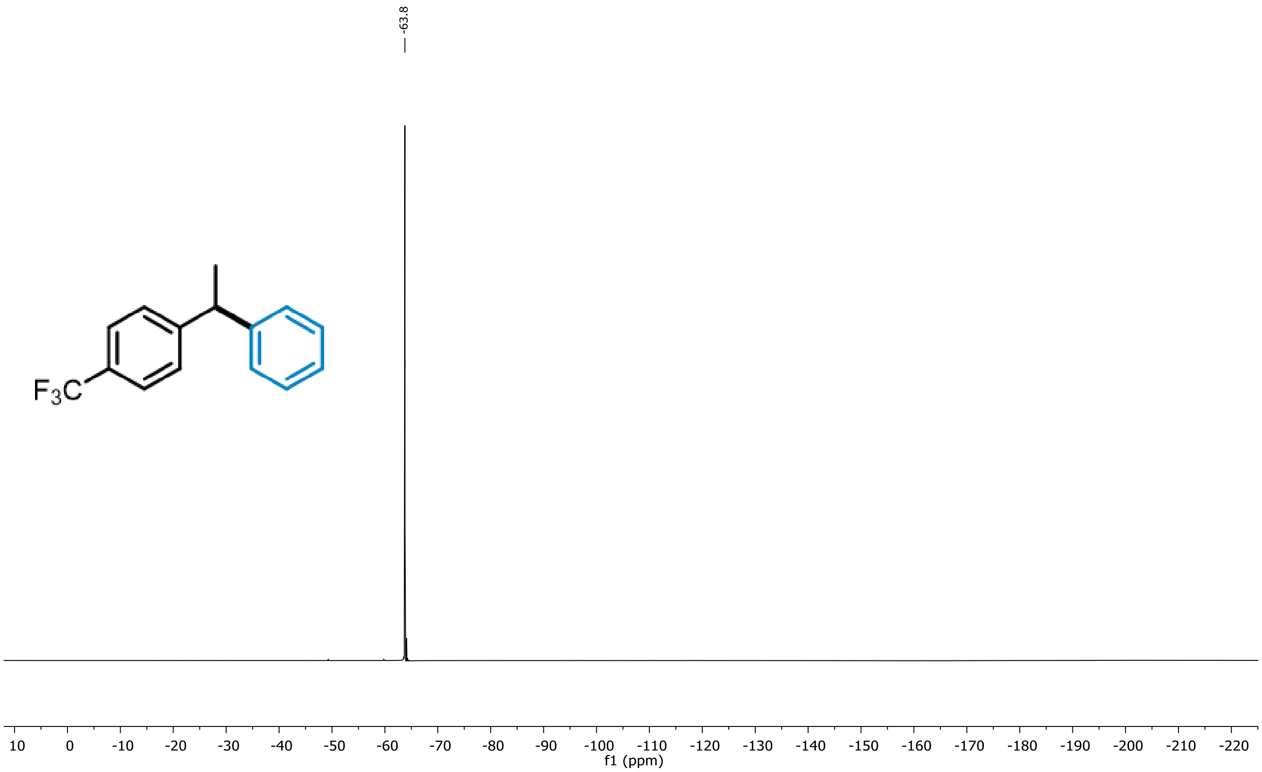

^{19}F NMR (376 MHz, $CDCl_3$) 1-(1-phenylethyl)-4-(trifluoromethyl)benzene (**3e**)

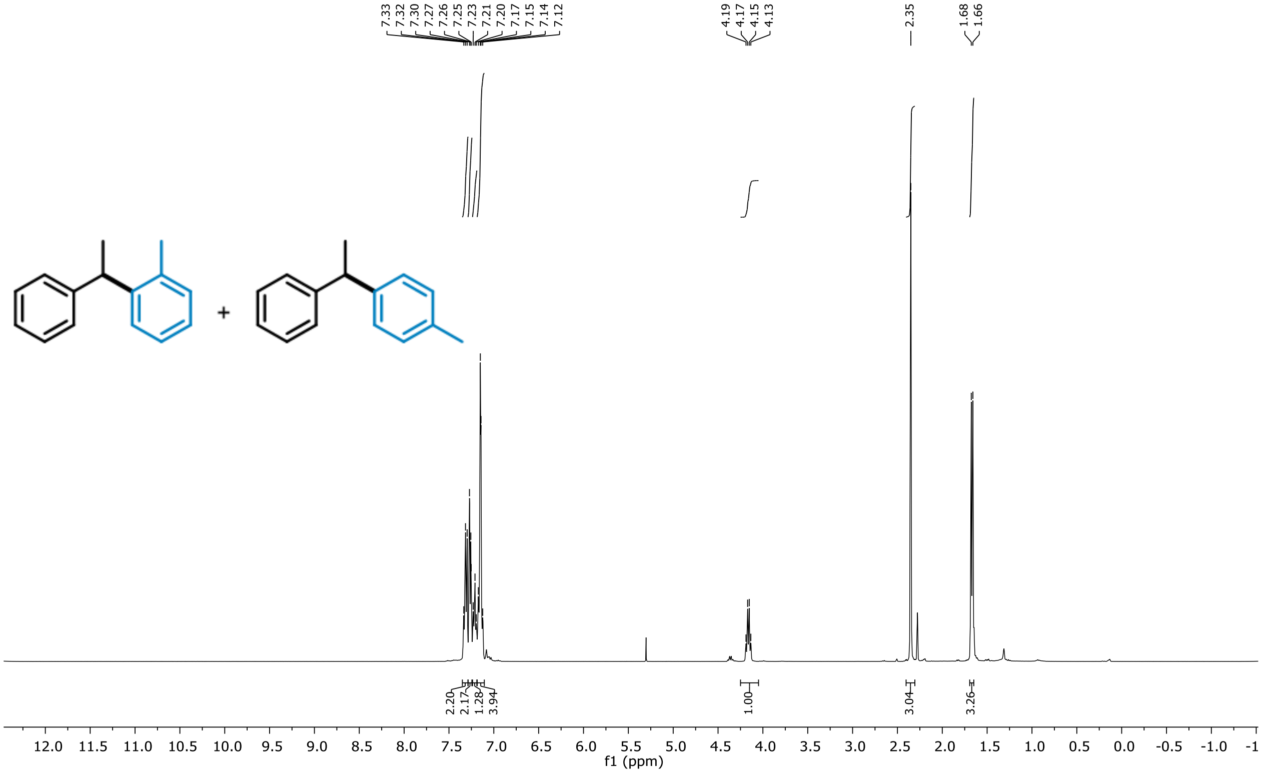

^{1}H NMR (400 MHz, $CDCl_3$) 1-methyl-2-(1-phenylethyl)benzene + 1-methyl-4-(1-phenylethyl)benzene (**3f**)

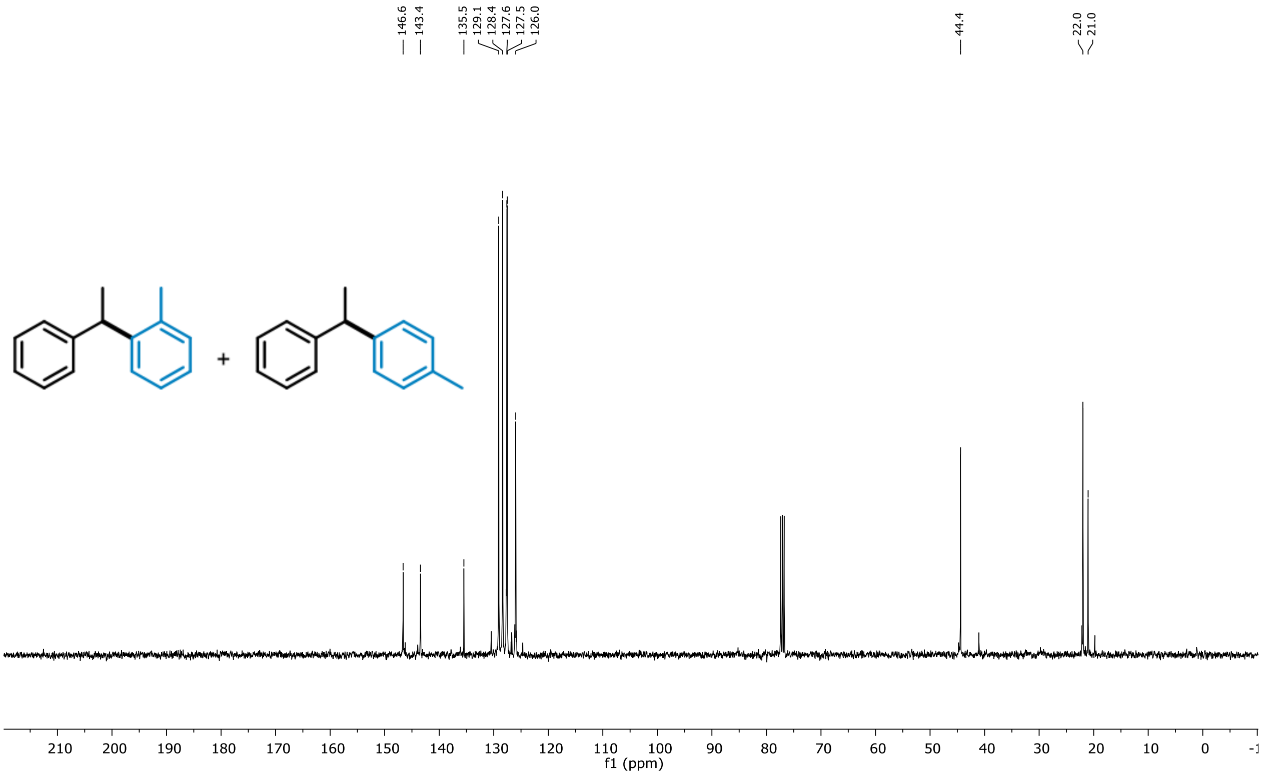

$^{13}C\{^{1}H\}$ NMR (101 MHz, $CDCl_3$) 1-methyl-2-(1-phenylethyl)benzene + 1-methyl-4-(1-phenylethyl)benzene (**3f**)

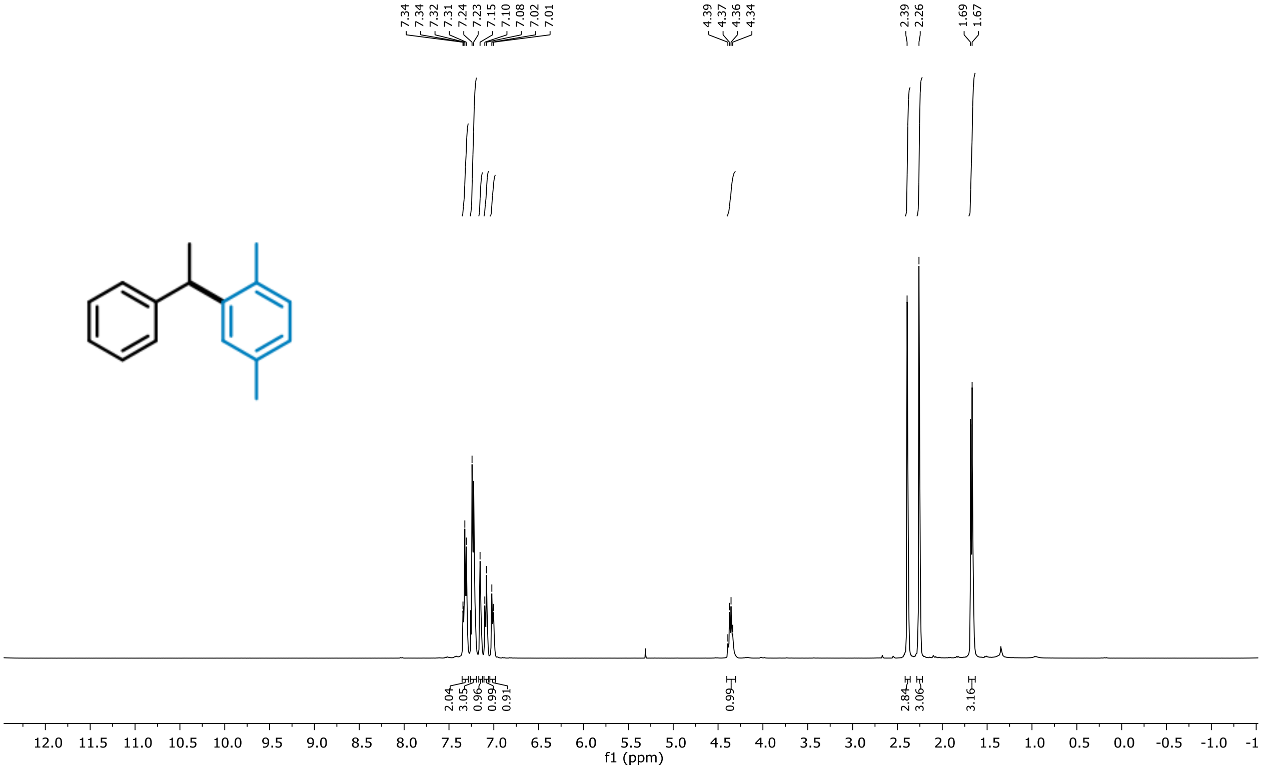

^{1}H NMR (400 MHz, $CDCl_3$) 1,4-dimethyl-2-(1-phenylethyl)benzene (**3g**)

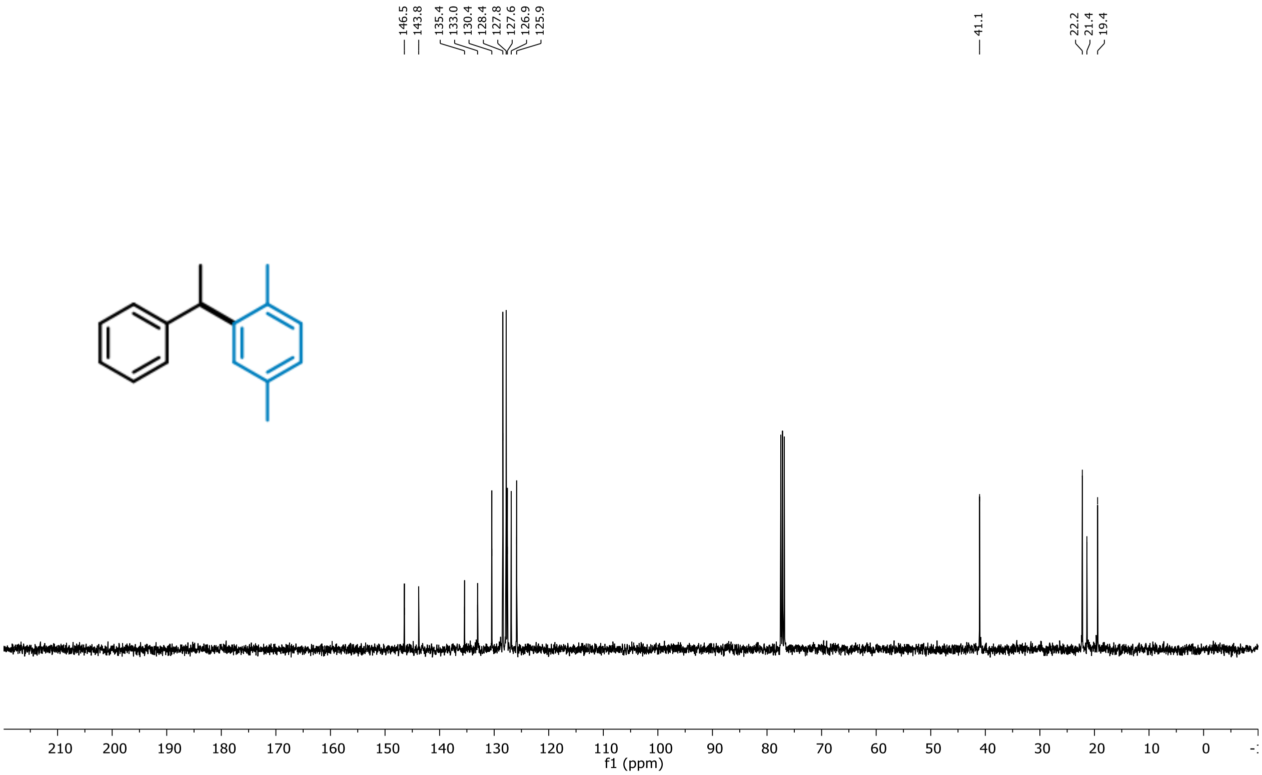

$^{13}C\{^1H\}$ NMR (101 MHz, $CDCl_3$) 1,4-dimethyl-2-(1-phenylethyl)benzene (**3g**)

^1H NMR (400 MHz, $CDCl_3$) diphenylmethane (**4a**) matches with compound **1a**.
$^{13}C\{^1H\}$ NMR (101 MHz, $CDCl_3$) diphenylmethane (**4a**) matches with compound **1a**.

^1H NMR (400 MHz, $CDCl_3$) 1-benzyl-4-methylbenzene (**4b**) matches with compound **1b**.
$^{13}C\{^1H\}$ NMR (101 MHz, $CDCl_3$) 1-benzyl-4-methylbenzene (**4b**) matches with compound **1b**.

^1H NMR (400 MHz, $CDCl_3$) 1-benzyl-4-chlorobenzene (**4c**) matches with compound **1d**.
$^{13}C\{^1H\}$ NMR (101 MHz, $CDCl_3$) 1-benzyl-4-chlorobenzene (**4c**) matches with compound **1d**.

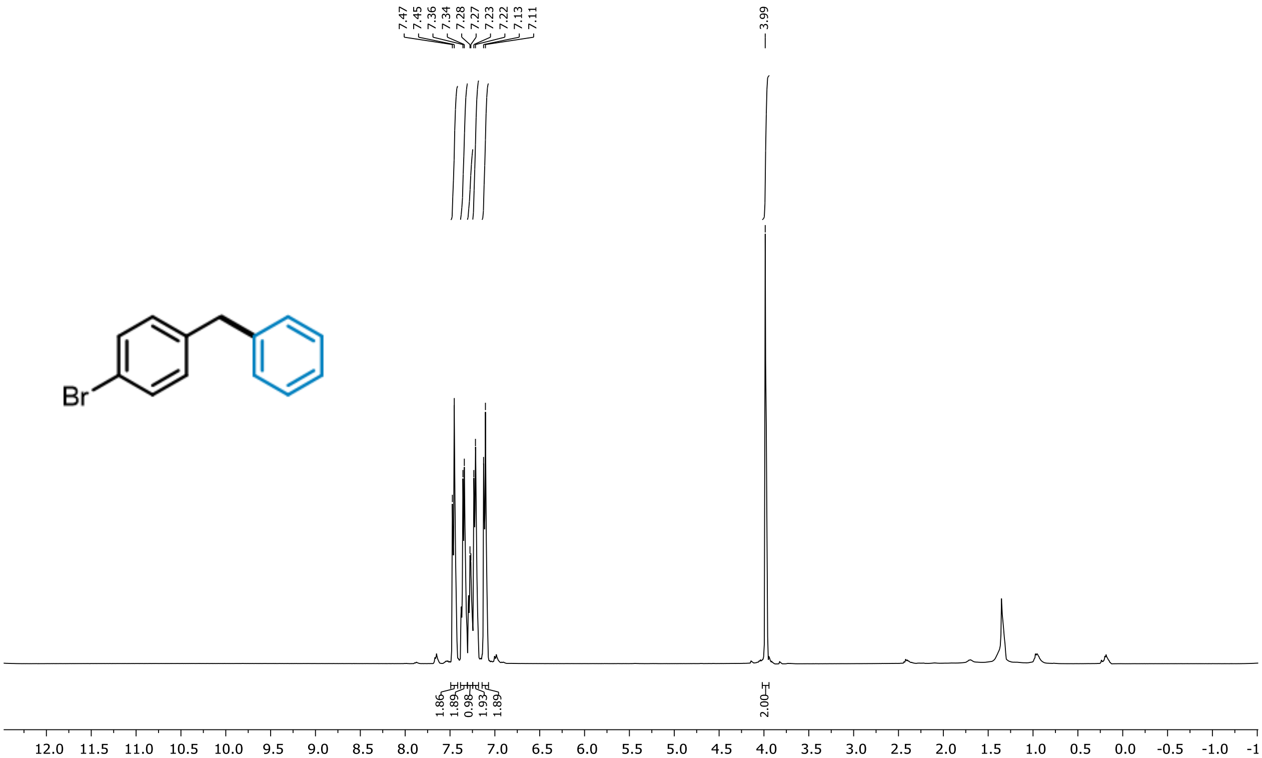

^{1}H NMR (400 MHz, $CDCl_3$) 1-benzyl-4-bromobenzene (**4d**)

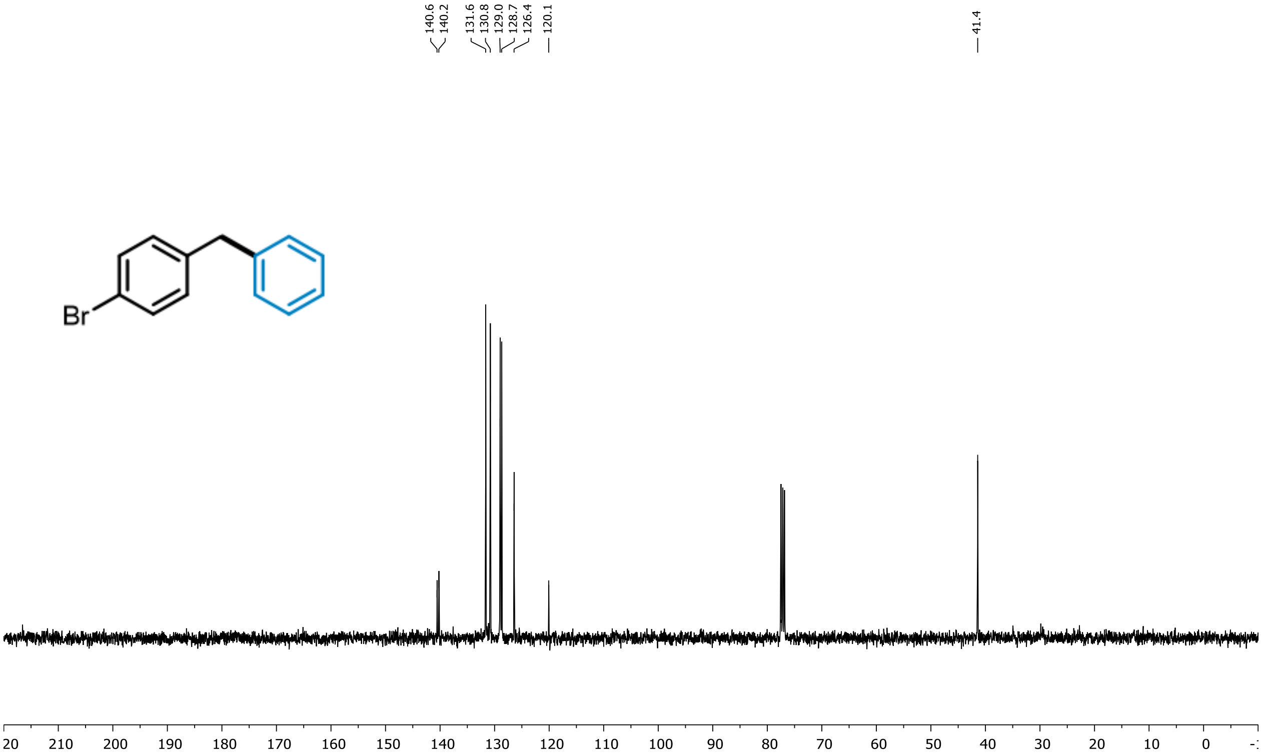

^{13}C{^{1}H} NMR (101 MHz, $CDCl_3$) 1-benzyl-4-bromobenzene (**4d**)

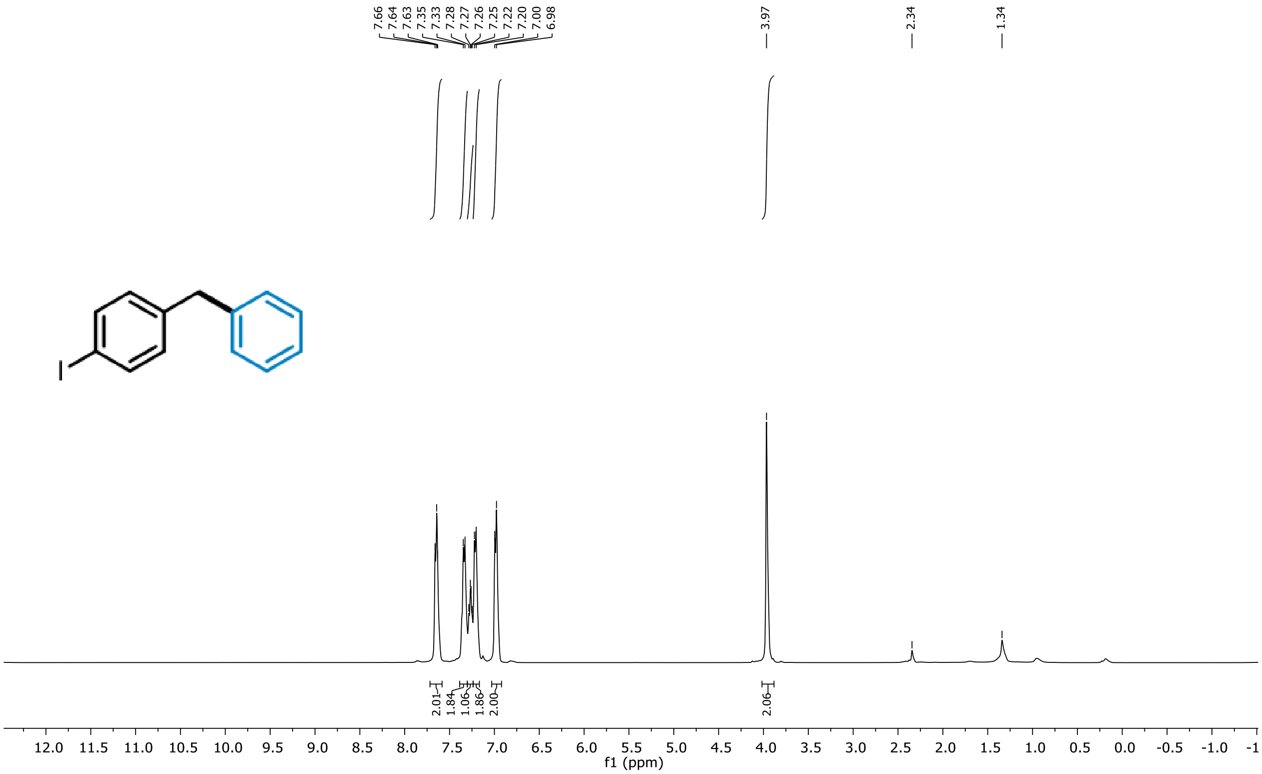

^{1}H NMR (400 MHz, $CDCl_3$) 1-benzyl-4-iodobenzene (**4e**)

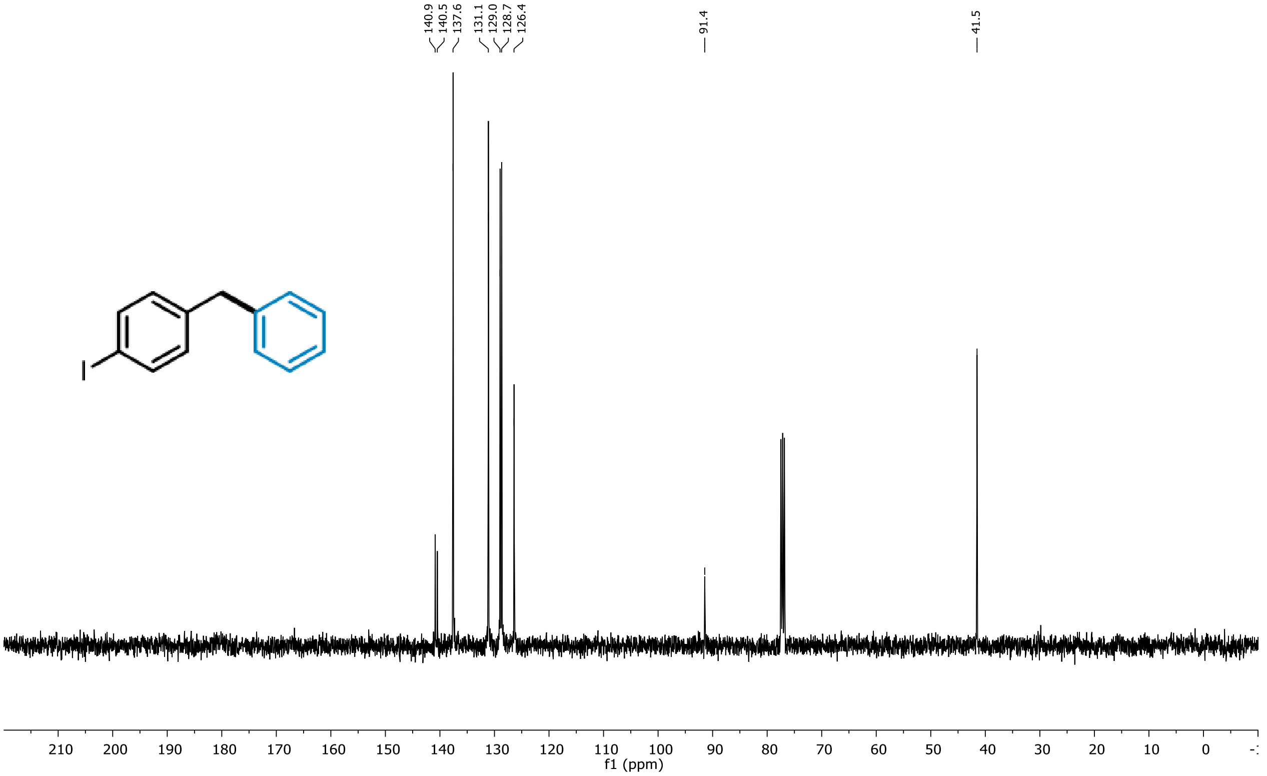

$^{13}C\{^{1}H\}$ NMR (101 MHz, $CDCl_3$) 1-benzyl-4-iodobenzene (**4e**)

^{1}H NMR (400 MHz, $CDCl_3$) 1-benzyl-2-methylbenzene + 1-benzyl-4-methylbenzene (**4f**) matches with compound **1e**.
^{13}C{^{1}H} NMR (101 MHz, $CDCl_3$) 1-benzyl-2-methylbenzene + 1-benzyl-4-methylbenzene (**4f**) matches with compound **1e**.

^{1}H NMR (400 MHz, $CDCl_3$) 2-benzyl-1,4-dimethylbenzene (**4g**) matches with compound **1g**.
^{13}C{^{1}H} NMR (101 MHz, $CDCl_3$) 2-benzyl-1,4-dimethylbenzene (**4g**) matches with compound **1g**.

^{1}H NMR (400 MHz, $CDCl_3$) diphenylmethane (**5a**) matches with compound **1a**.
^{13}C{^{1}H} NMR (101 MHz, $CDCl_3$) diphenylmethane (**5a**) matches with compound **1a**.

^{1}H NMR (400 MHz, $CDCl_3$) 1-benzyl-4-methylbenzene (**5b**) matches with compound **1b**.
^{13}C{^{1}H} NMR (101 MHz, $CDCl_3$) 1-benzyl-4-methylbenzene (**5b**) matches with compound **1b**.

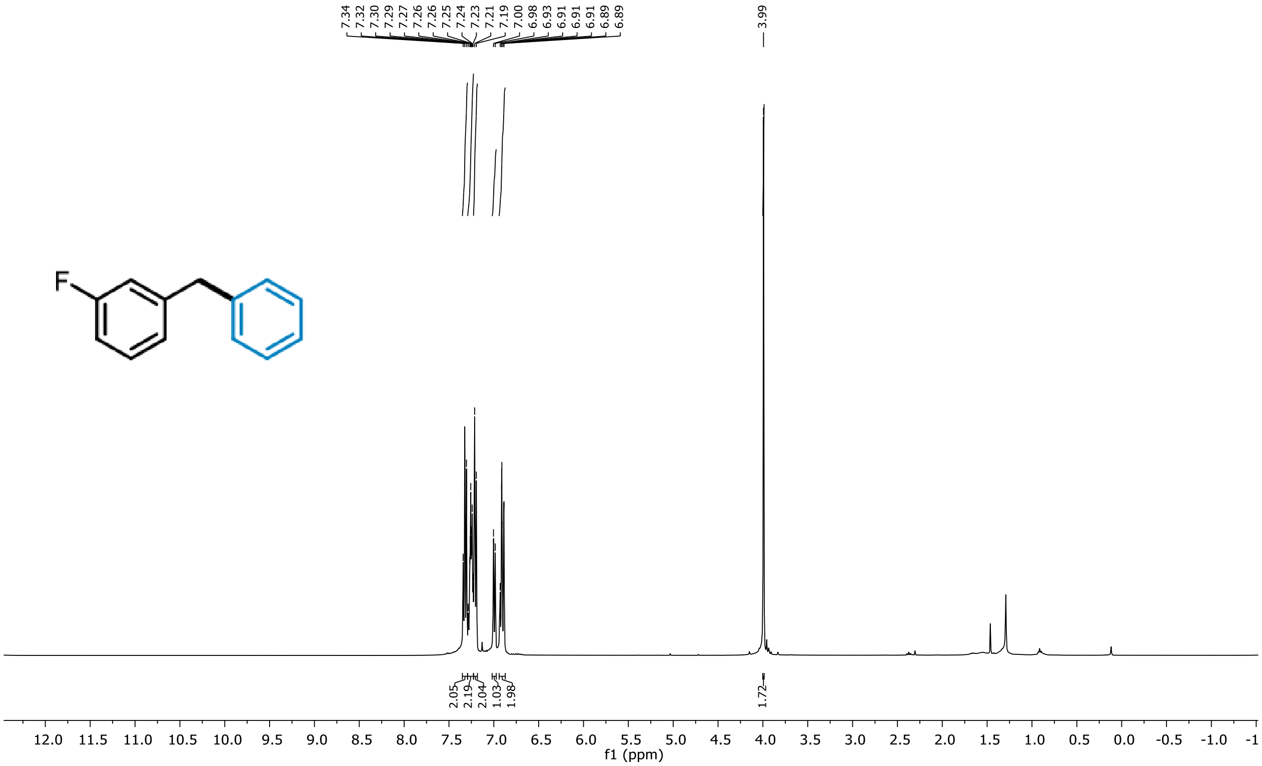

^{1}H NMR (400 MHz, $CDCl_3$) 1-benzyl-3-fluorobenzene (**5c**)

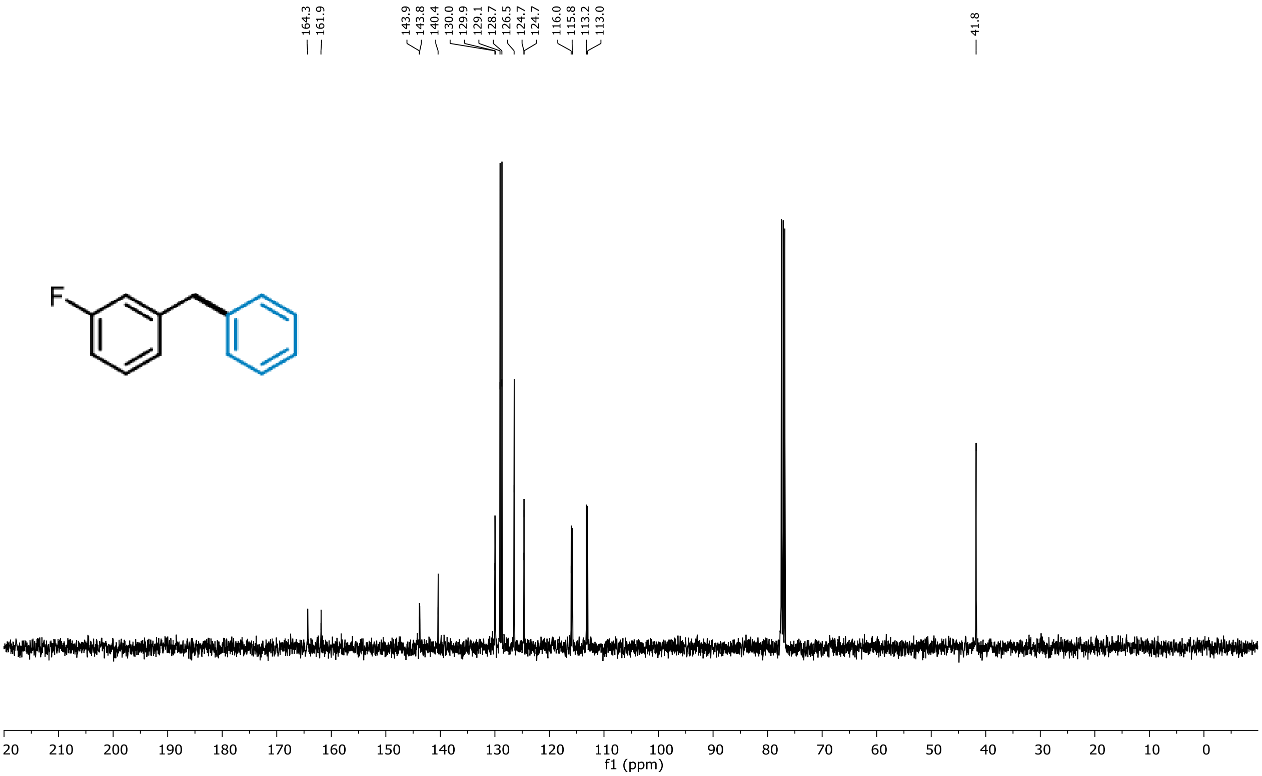

$^{13}C\{^{1}H\}$ NMR (101 MHz, $CDCl_3$) 1-benzyl-3-fluorobenzene (**5c**)

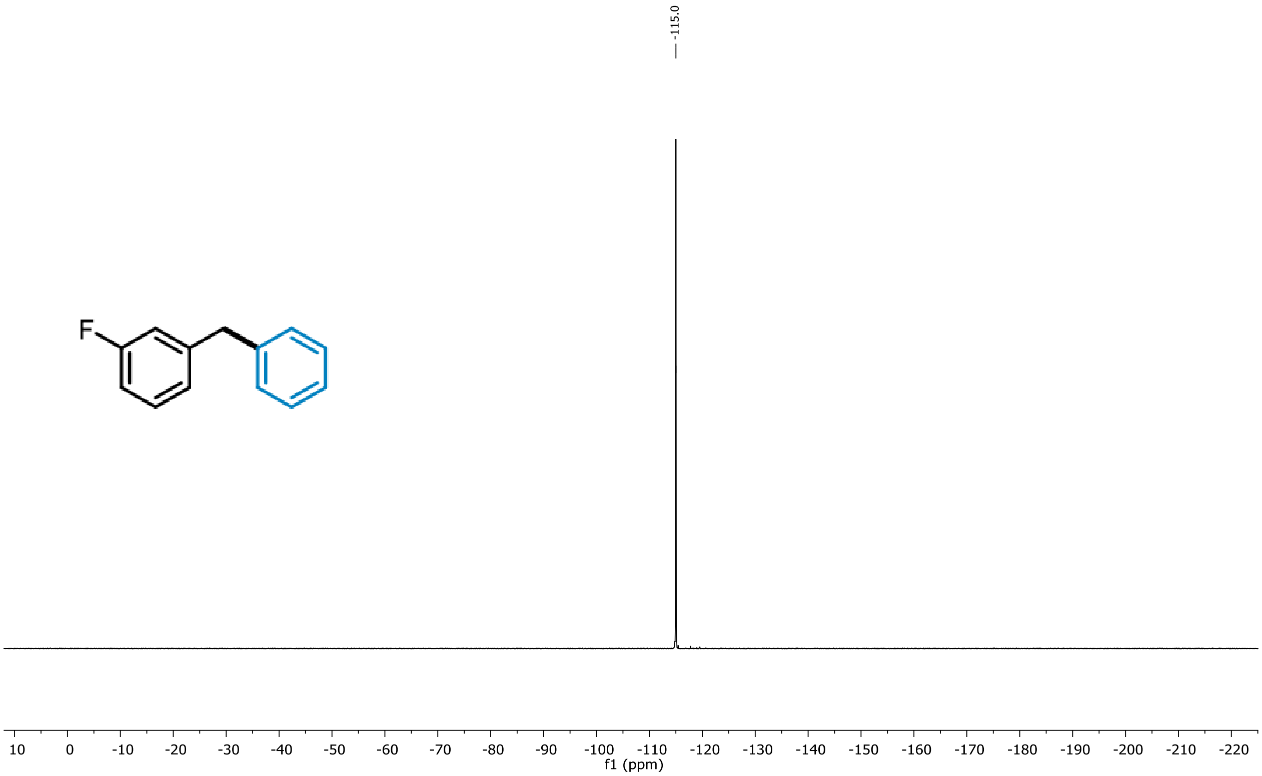

^{19}F NMR (376 MHz, $CDCl_3$) 1-benzyl-3-fluorobenzene (**5c**)
